# Natural Flavonoid-Derived Enzyme Mimics DHKNase Balance the Two-Edged Reactive Oxygen Species Function for Wound Healing and Inflammatory Bowel Disease Therapy

**DOI:** 10.34133/research.0464

**Published:** 2024-09-09

**Authors:** Guangfu Feng, Huaizu Zhang, Huipeng Liu, Xiaoyan Zhang, Hongmei Jiang, Sijie Liao, Xingyu Luo, Hao Yao, Bo Xiang, Shiyu Liu, Jiali Zhang, Jiaheng Zhang, Jun Fang

**Affiliations:** ^1^ School of Bioscience and Biotechnology, Hunan Agricultural University, Changsha, Hunan 410128, P.R. China.; ^2^ College of Life Science, Shihezi University, Shihezi, Xinjiang 832003, P.R. China.; ^3^ School of Chemistry and Chemical Engineering, Guangxi University, Nanning 530004, P.R. China.; ^4^ Changsha IMADEK Intelligent Technology Co. Ltd., Changsha, Hunan 410081, P.R. China.; ^5^ College of Chemistry, Food Laboratory of Zhongyuan, Flavour Science Research Center of Zhengzhou University, Zhengzhou University, Zhengzhou, Henan 450001, P.R. China.

## Abstract

Rational regulation of reactive oxygen species (ROS) plays a vital importance in maintaining homeostasis of living biological systems. For ROS-related pathologies, chemotherapy technology derived from metal nanomaterials currently occupies a pivotal position. However, they suffer from inherent issues such as complicated synthesis, batch-to-batch variability, high cost, and potential biological toxicity caused by metal elements. Here, we reported for the first time that dual-action 3,5-dihydroxy-1-ketonaphthalene-structured small-molecule enzyme imitator (DHKNase) exhibited 2-edged ROS regulation, catering to the execution of physiology-beneficial ROS destiny among diverse pathologies in living systems. Based on this, DHKNase is validated to enable remarkable therapeutic effects in 2 classic disease models, including the pathogen-infected wound-healing model and the dextran sulfate sodium (DSS)-caused inflammatory bowel disease (IBD). This work provides a guiding landmark for developing novel natural small-molecule enzyme imitator and significantly expands their application potential in the biomedical field.

## Introduction

Reactive oxygen species (ROS) are the types of oxygen-containing chemically reactive molecules that are acknowledged to be closely involved with physiological functions and health benefits, such as regulating cell behaviors, antibacterial activity, and tumor suppression [1–3]. Especially, ROS plays particular and 2-edged roles in living systems for balancing biological homeostasis [4–6]. Naturally, the controllable ROS stress allows the recovery of biological functions and is indispensable during subtle body repairing, such as microbe-induced wound ulcers [7–9]. Nonetheless, the uncontrolled or excessive production of ROS will result in irreversible oxidative cell damage and cause a myriad of pathologies, including inflammatory diseases [10–12]. Imbalance in the body of the ROS metabolism leads to a series of diseases, including ROS deficiency-caused genetic defect-related rheumatoid arthritis [13], chronic granulomatous [14], thyroid disease [15], bacteria defense failure [16], ROS accumulation-caused Alzheimer’s disease [17], atherosclerosis [18], nephritis [19], diabetes [20], asthma [21], rheumatoid arthritis [22], osteoporosis [23], and inflammatory bowel disease (IBD) [24,25]. Consequently, rationally regulating in vivo ROS levels through chemical or biomedical techniques remains a significant hotspot but is challenging.

Chemotherapy approaches derived from metallic nanomaterials have attracted growing interest in treating ROS-related pathologies and are being developed as nanomedicines to manifest a remarkable therapeutic effect [26–35]. However, concerns regarding biotoxicity and unavoidable adverse effects caused by the metal-contained catalytic centers limit their applicability in addressing complicated pathogenesis in vivo [36,37]. More importantly, sophisticated synthesis and inhomogeneous batch-to-batch efficacies hinder their repeatability and clinical availability [38,39]. To address the aforementioned deficiencies, natural compounds are favored as the primary class of effective pharmaceuticals for treating diverse pathologies [40–44]. For instance, on account of the beneficial antioxidant activities, high biocompatibility, low toxicity, and immune enhancement, secondary polyphenol metabolites in plants are receiving considerable focus in treating numerous diseases, including neurodegenerative disorders, cancer inhibition, hepatitis, and antiviral infection [45–47]. Nevertheless, polyphenol metabolites have not yet been reported to exhibit dual-action ROS shunt for physiology-programmed oxidative stress modulation in a living system.

**Fig. 1.**
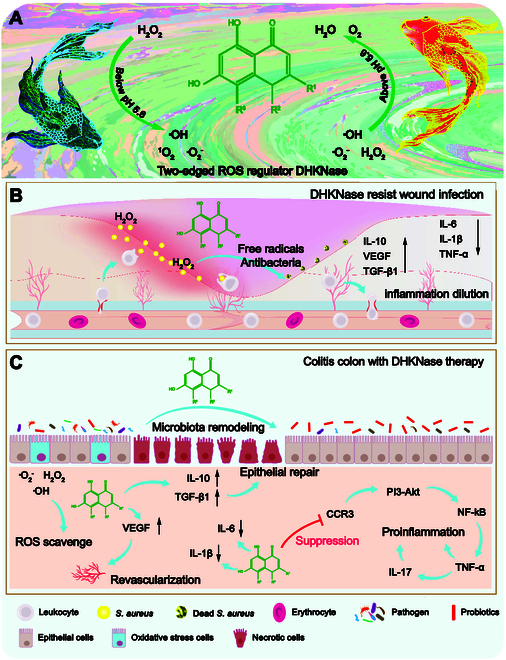
The dual action enzyme imitator DHKNase for anti-infection wound healing and anti-inflammation IBD therapy. (A) The dual-action DHKNase for free radicals’ supply or scavenge in a boundary of pH 5.6. (B) DHKNase converts free radicals to eliminate the infectious bacteria in the wound. (C) DHKNase scavenges ROS in the inflammation colon to alleviate IBD with intestinal flora improvement, micro-environment repair, and host inflammatory metabolic pathway regulation.

In this work, DHKNase is found to show 2-edged regulation of oxidative stress in living systems. Specifically, DHKNase displays unexpected peroxidase (POD)-like activity to catalyze the hydrogen peroxide (H_2_O_2_) decomposition via the hydroxyl radical (·OH)-generating pathway when pH was below 5.6. Meanwhile, the bifacial DHKNase enables endogenous ROS elimination at atmosphere of pH above 5.6 (Fig. 1A). To our best knowledge, this discloses the first case of such natural small molecules exhibiting valve-shunt control of ROS. Based on this, it is verified that DHKNase gives multipurpose therapeutic effects on the pathogen-infected superficial wound and the dextran sulfate sodium (DSS)-induced IBD colon (Fig. 1B and C). In the superficial wound, DHKNase demonstrates superior anti-infection performance due to the supplement of oxidative free radicals even with a low H_2_O_2_ concentration. By down-regulating the pro-inflammatory factors’ expression and up-regulating the anti-inflammatory factors’ expression, DHKNase effectively reduces the inflammatory response and accelerates wound healing. In the IBD colon, DHKNase scavenges the excessive ROS and remodels intestinal flora composition and gene expression, assisting in colon psychological transformation from inflammation to normalcy. By down-regulating the pathogenic bacteria abundance and up-regulating the probiotics abundance, the intestinal microorganisms’ composition is reestablished to resemble that of a robust intestinal community. Transcriptomic analysis confirms the elimination of host colon inflammation and the recovery of healthy metabolism, in which the down-regulation of the CCR3 gene is examined as most likely to be the therapeutic mechanism. Given the remarkable efficiency in infectious wound healing and IBD therapy, DHKNase is anticipated to have great potential as the precursor for anti-infection and anti-inflammation drug synthesis in more extensive ROS-related biomedical applications.

## Results

### Two-edged ROS regulation by DHKNase-*6*

**Fig. 2.**
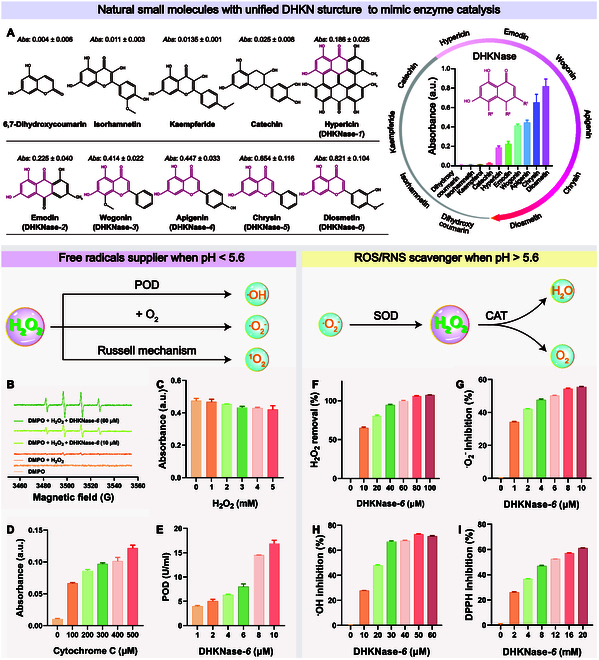
DHKNase acted as the ROS supplier in an acidic atmosphere or ROS/RNS scavenger in a neutral atmosphere. (A) Natural DHKN-structured compounds for enzyme imitation in an acidic atmosphere. The chemical formula of natural compounds and the red-marked unified DHKN structure were shown on the left, and the right side demonstrated their capacity to decompose hydrogen peroxide and oxidize TMB. (B to D) ESR and free radical-trapping experiments identified the liberation of singlet oxygen (^1^O_2_), ·OH, as well as the superoxide anion (·O_2_^−^) during the catalysis, respectively. 5,5-Dimethyl-1-pyrolin-N-oxide (DMPO) exhibited elevated resonance signal in the presence of ·OH. The absorption of 9,10-diphenylanthracene (DPA) was suppressed by ^1^O_2_, and the absorption of cytochrome c increased when there was ·O_2_^−^ in the solution. (E) POD-like activity of DHKNase*-6* tested by the assay kit. (F to I) H_2_O_2_, ·O_2_^−^, ·OH, and 2, 2-diphenyl-1-picrylhydrazyl (DPPH) removal by DHKNase*-6* in PBS buffer (pH 7.4). The removal of H_2_O_2_ was tested using a color rendering method based on ammonium molybdate. The ·O_2_^−^ removal test was performed using a superoxide suppression kit. The Cu^2+^-based Fenton reaction was used to provide ·OH in the solution. Results were from 3 independent experiments. Graphs were shown as mean ± SEM.

The multiple enzyme-like abilities and dual-action ROS shunt characteristics of DHKNase were evaluated utilizing typical substrates of natural enzymes. To identify the enzyme-mimicking property of DHKNase, 3,3′,5,5′-tetramethylbenzidine (TMB) was adopted for colorimetric indication. When free radicals are present in the solution, the TMB changes from colorless to blue. Significantly improved absorption of oxidized TMB (oxTMB; 652 nm) was observed after the compounds with a 3,5-dihydroxy-1-ketonaphthalene (DHKN) moiety were added to the solution. In contrast, compounds without or with impaired DHKN structure exhibited negligible absorption disturbance (Fig. 2A), in which the most noticeable intensity change was obtained after the addition of DHKNase-*6* (diosmetin). Meanwhile, it was proved that the acidic atmosphere, DHKNase*-6*, and H_2_O_2_ were indispensable for TMB oxidation, and the pH-responsive threshold for DHKNase*-6* to mimic catalysis was defined as pH 5.6 (Figs. S1 and S2). For the pH-responsive threshold, DHKNase-*6* exercises its ability to mimic enzyme catalysis by supplying reactive radicals to the solution in an environment with a pH below this limit. However, DHKNase-*6* exhibits a strong radical clearing effect while the pH exceeds the threshold, defined as the pH-dependent valve-shunt control of ROS. DHKNase-*6* demonstrated better tolerance toward temperature, salinity, and pH in comparison to the endogenous enzyme horseradish peroxidase (HRP) (Fig. S3) while maintaining 83% of catalytic activity even after storage at 4 °C for 417 d (Fig. S4). Meanwhile, the possibility of impurity metal ion-mediated Fenton reaction to trigger TMB oxidation was excluded (Table S1 and Figs. S5 and S6).

To verify the supplement of free radicals by DHKNase*-6* in an atmosphere of pH below 5.6, typical scavengers were selected and employed (Fig. S7A). It was observed that the absorption intensity of oxTMB was reduced after the addition of the ·OH scavengers (Fig. S7B). Electron spin resonance (ESR), p-phthalic acid (PTA) fluorescence, 8-hydroxyquinoline, and isopropyl alcohol experiments also proved the production of ·OH during the reaction (Fig. 2B and Fig. S7C to E). Except for ·OH, ^1^O_2_ and ·O_2_^−^ were also classified in the solution (Fig. 2C and D and Fig. S7F). The POD-like capability of DHKNase*-6* was further identified by the enzyme-substrate experiment with no disturbance to the environment pH value (Fig. 2E and Fig. S8). As shown in Fig. S9 and Table S2, the standard Michaelis–Menten plots confirmed the excellent affinity of DHKNase-*6* toward H_2_O_2_. Notably, the efficient depletion of H_2_O_2_ during the reaction could afford DHKNase*-6* the sensing potential of ambient H_2_O_2_ concentration (Fig. S10).

Furthermore, the ROS/RNS scavenging capabilities of DHKNase-*6* were verified while the environment pH exceeded 5.6. ROS, including H_2_O_2_, ·O_2_^−^, and ·OH, were efficiently removed following DHKNase-*6* incubation, as depicted in Fig. 2F to H. The kit-certified catalase (CAT)- and superoxide dismutase (SOD)-like activities could explain that DHKNase-*6* removed a large amount of ROS at a small concentration (Fig. S11). Unexpectedly, it was also obtained that DHKNase-*6* could remove reactive nitrogen species (RNS) (Fig. 2I and Fig. S12). Together, DHKNase-*6* exhibited pH-dependent characteristics of multiple enzyme imitation for 2-edged ROS regulation, either ROS supplier or scavenger.

### Anti-infection and accelerated healing in the superficial wound

Alternative strategies for treating pathogenic bacterial infections have attracted growing attention due to antibiotic abuse-triggered multidrug resistance. It was reported that the epidermal layer of the skin exhibits an acidic atmosphere [48–51]. Meanwhile, spontaneously encouraged by the outstanding free radical-supplying capability of DHKNase-*6* in an atmosphere of pH below 5.6, the dynamic therapy for infectious wounds was investigated. First, the ability of DHKNase-*6* to eliminate pathogenic microorganisms was tested (Fig. S13A). Images of pathogenic bacteria growth on plates (Figs. S13B and S14) and in suspension (Fig. S15) revealed the broad-spectrum and efficient antibacterial property of DHKNase-*6,* in which the used H_2_O_2_ concentration was not hazardous to the bacteria. Meanwhile, bacteria DNA damage was observed after the DHKNase-*6* (+) treatment (Figs. S16 and S17). Scan electron microscopy (SEM) photographs evidenced the severe membrane defect of *Staphylococcus aureus*, as well as *Escherichia coli*, accounting for the DNA efflux and damage in a radical-abundant atmosphere (Fig. S18).

**Fig. 3.**
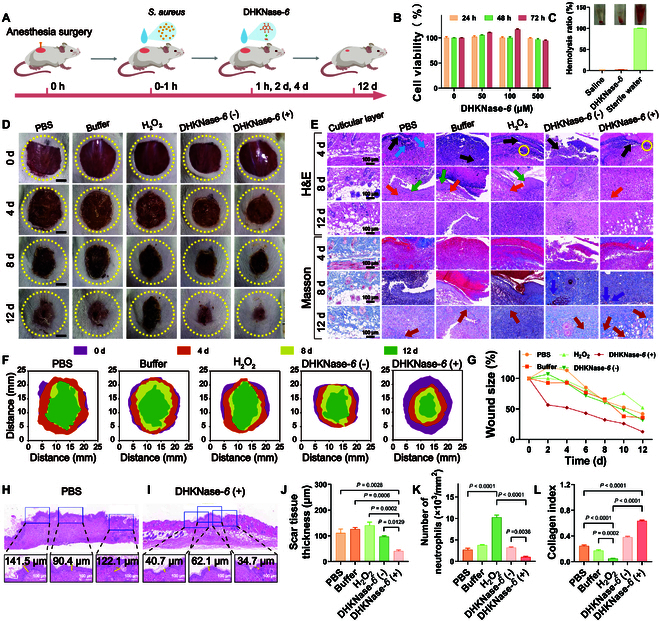
Acceleration of the wound healing by DHKNase*-6* under the stress of pathogenic bacteria infection. (A) Cartoon illustration of the wound healing experiment. Briefly, a wound of *D* ≈ 18.0 mm on the mice back was created with anesthesia and surgical procedures. *S. aureus* was dripped onto the wound with 1.0 × 10^7^ colony-forming units per milliliter. After 1-h infection, formulations were sprinkled on the wounds for a total of 3 treatments every other day. (B and C) Three-day *Caco-2* cell viability (*n* = 3) and hemolysis (*n* = 3) experiment indicated the admirable biocompatibility of DHKNase*-6* (*n*, numbers of independent experimental sets; mean ± SEM). After incubation with DHKNase*-6*, the survival of the *Caco-2* cells was evaluated by a methylthiazolyldiphenyl-tetrazolium bromide (MTT) kit. Mouse orbital blood cells were used for the hemolysis experiment. (D) Daily wound (*n* = 3) photograph revealed the accelerated wound closure after DHKNase-*6* (+) treatment (*n*, numbers of mice). DHKNase-*6* (−): DHKNase-*6* + buffer; DHKNase-*6* (+): DHKNase-*6* + buffer + H_2_O_2_. DHKNase-*6* (−) and DHKNase-*6* (+) were set to prove that the elimination of *S. aureus* veritably came from the highly toxic free radicals converted by DHKNase-*6* regarding the normal physiological wound microenvironment in the initial infection, but not the acidic environment resulting from the accumulation of acidic pus in the late infection. Scale bar, 5 mm. (E) H&E images (*n* = 3) of the mice wound tissues, in which the blue, black, purple, orange, green, and red arrows and yellow circle represented the inflammatory cell infiltrations, local hyperemia, regenerative blood vessels, dermal fibroblasts, wound tears, collagen fibers, and disintegration of skin and collagen, respectively (*n*, numbers of mice). Scale bar, 100 μm. Wound tissues were collected with surgical blades after the mice were sacrificed, then rinsed with PBS solution, and preserved in 4% paraformaldehyde solution before staining. (F) Top-view stack of the daily wounds from (D), demonstrating the lowest open wound area after DHKNase-*6* (+) treatment. The wound shapes were depicted based on the unhealed part of the wounds. (G) Accelerated wound closure (*n* = 3) with DHKNase-*6* (+) treatment (*n*, numbers of mice; mean ± SEM). (H to J) Evaluation of scar thickness through H&E staining results of the superficial wounds (*n* = 3) on the 12th day, showing the lowest scar thickness after DHKNase*-6* (+) treatment (*n*, numbers of biological independent samples; mean ± SEM). Scale bar, 100 μm. (K and L) Decreased neutrophil cell infiltration and increased collagen deposition (*n* = 3) in the wounds after DHKNase-*6* (+) treatment on the 12th day (*n*, numbers of biological independent samples; mean ± SEM). ImageJ software was used to count the neutrophil cells and calculate the collagen deposition index. One-way analysis of variance (ANOVA) test.

Next, the utility of DHKNase-*6* in accelerating infectious wound healing was thoroughly investigated (Fig. 3A). Male BALB/c mice were surgically inflicted with superficial incisions under anesthesia, and *S. aureus* suspensions were then applied to stimulate infection and inflammation wounds. As shown in Fig. S19, there was a notable rise in inflammatory cell infiltration, local edema, wound tear, as well as skin and collagen degradation at the wound site, indicating the successful establishment of the wound infection and inflammation. The high cell viability (Fig. 3B), low blood cell fragmentation efficacy (Fig. 3C), and no visible mice weight loss (Fig. S20) indicated that DHKNase-*6* possessed admirable biocompatibility and displayed no health side effects on mice. To aid in evaluating the healing process, the wound photographs and the corresponding histology-staining images were recorded (Fig. 3D and E). It was observed that the introduction of DHKNase-*6* (+) led to the fastest wound closure on day 12 due to the suitable working conditions for DHKNase-*6* (Fig. 3F and G and Fig. S21). Furthermore, multiple-dose administration had also been shown to have no effect on the acidic microenvironment of the skin wound (Fig. S22).

To further evaluate the wound infection, the bacterial count on the wound was investigated. As depicted in Fig. S23, DHKNase-*6* (+) treatment significantly reduced the bacteria colony, indicating effective resistance to the *S. aureus* infection. Evaluation of hematoxylin and eosin (H&E), for cell morphology reference, and Masson’s trichrome colored slice, for collagenous fiber analysis, revealed that on the fourth day, all groups exhibited profuse inflammatory cell infiltration (blue arrow), the disintegration of epidermis and collagen fibers (yellow circle), and local hyperemia (black arrow). Tissues from the DHKNase*-6* (+) group had been well epithelialized on the eighth day with neonatal blood vessels (purple arrow) and dermal fibroblasts (orange arrow). However, after the same healing interval, the wound tears (green arrow) were still noticed in the phosphate-buffered saline (PBS), buffer, and H_2_O_2_ groups. On the 12th day, the minimal thickness of scar tissue following DHKNase-*6* (+) treatment was also observed (Fig. 3H to J and Fig. S24), attributing to the reduced persistence of tissue inflammation, avoiding the imbalance of collagen synthesis, degradation, and generation of abnormal mucopolysaccharides [52]. Cell counting data revealed a minimal number of neutrophils in the DHKNase-*6* (+) group, indicating the factual alleviation of inflammatory response after DHKNase-*6* (+) treatment (Fig. 3K). It is worth noting that there was a large increase of neutrophil cells in the H_2_O_2_ group, which might be because the non-antibacterial low-concentration H_2_O_2_ promoted an earlier transition of the wound inflammatory state. In contrast to other groups, the DHKNase-*6* (+) group had completed better regeneration of dermis tissue and deposited more robust collagen fibers (red arrow) that were regular, orderly, and structurally intact with the epidermis. As depicted in Fig. 3L, the DHKNase-*6* (+) group exhibited the most significant amount of collagen deposition, implying a more effective wound healing. Furthermore, the healthy physiology of the kidney, spleen, liver, lungs, and heart further demonstrated that the DHKNase-*6* treatment brought no discernible adverse effects to the host mice (Fig. S25).

**Fig. 4.**
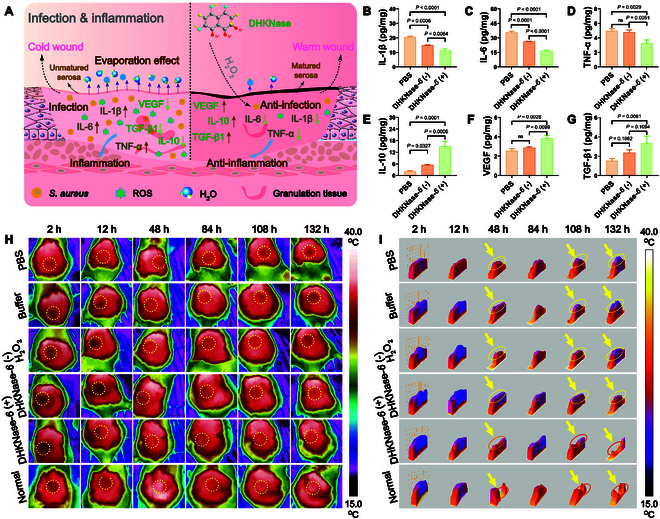
DHKNase*-6* regulated the cytokine expression and helped temperature recovery in a bacteria-infected wound. (A) Brief illustration of DHKNase*-6*-mediated pathogenic bacteria elimination, wound temperature retention, and cytokine regulation. (B to G) The DHKNase*-6* (+) treatment down-regulated the IL-1β, IL-*6*, and TNF-α expression but up-regulated the level of IL-10 and growth factors VEGF and TGF-β1 (*n* = 3) in the *S. aureus*-infected mice wound (*n*, numbers of mice; mean ± SEM). After 3 days of the first treatment, wound tissues were collected. The tissue was homogenized, and the supernatant was retained for the ELISA tests. (H and I) Thermography of the wound temperature and the temperature fluctuation (*n* = 3) that covered the center of the wound as well as the edges, showing that the temperature recovery of the wound after DHKNase-*6* (+) treatment was more similar to that of the uninfected wound (*n*, numbers of mice; Normal, the surgical wounds without *S. aureus* infection). ImageJ software was used to evaluate the wound temperature fluctuation. One-way ANOVA test.

To further explore the mechanism of DHKNase*-6* in suppressing the inflammatory response and promoting wound healing (Fig. 4A), inflammation- and tissue healing-related chemokines had been examined. As a proof of concept, wound tissues involving cytokines that impacted the wound physiology on the third day were harvested and measured with enzyme-linked immunosorbent assay (ELISA) assays. With DHKNase-*6* (+) treatment, the levels of inflammation-promoted cytokines interleukin-1β (IL-1β), IL-6, and tumor necrosis factor-α (TNF-α) were down-regulated, while the level of inflammation-deterrent factor IL-10 was up-regulated, demonstrating a decreased inflammatory tendency (Fig. 4B to E). Alternatively, the elevated levels of the growth-related vascular endothelial growth factor (VEGF) and transforming growth factor-β1 (TGF-β1) promoted angiogenesis and cell proliferation at the wound site, thereby facilitating the removal and replacement of damaged cells (Fig. 4F and G). Accordingly, wounds treated with DHKNase*-6* (+) gained accelerated inflammation diminishment and tissue healing. Besides, it was acknowledged that wound hypothermia significantly delays the healing process [53,54]. Specifically, bacterial infection allows persistent erosion and water evaporation in the wound, leading to a huge temperature drop, and eradicating this pathology would considerably help temperature recovery and accelerate wound healing. To verify the abbreviated temperature recovery at the wound site by DHKNase*-6* (+) treatment, infrared photographs of the superficial lesions were recorded (Fig. 4H). In the first 2 h, the wound temperature declined 3.0 to 4.0 °C for all groups due to a lack of full-layer epidermis, which caused a significant amount of heat loss and thermal insulation failure (Fig. 4I). After the next 48 h, the DHKNase-*6* (+)-treated wound showed a temperature drop of 1.16 °C, while the other groups continued to suffer temperature drops greater than 1.96 °C. As the wounds went through 108 h of healing, a similar temperature value was observed between the DHKNase*-6* (+)-treated and uninfected lesions, suggesting robust wound physiology after DHKNase*-6* (+) treatment (Fig. S26). Overall, DHKNase*-6* promoted the healing process in bacterial-infected superficial wounds by eliminating the pathogen, down-regulating pro-inflammatory chemokines, up-regulating anti-inflammatory cytokines, and preventing wound hypothermia.

### Effective IBD therapy by DHKNase-*6*

**Fig. 5.**
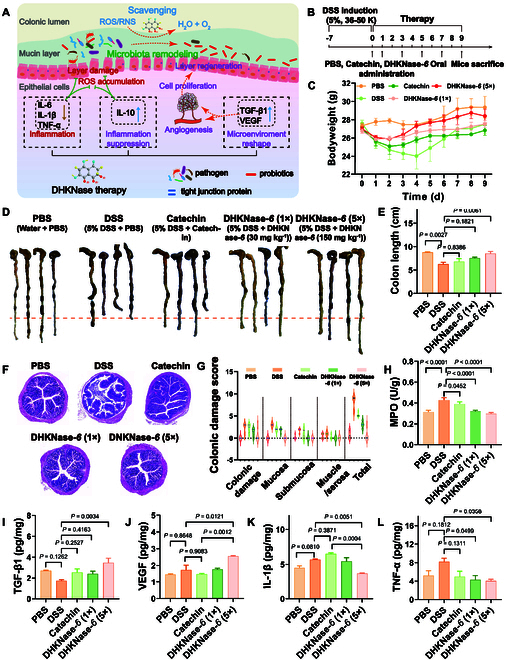
DHKNase*-6* relieved colonic inflammation in male BALB/c mice. (A) DHKNase*-6* remodeled microbiota, scavenged ROS, and regulated cytokines and epithelial cell proliferation in the DSS-induced mice colitis colon. (B) After acclimatization, male BALB/c mice were employed for 7-d feeding of 5% (w/v) DSS water (36 to 50 K) to set up the colon inflammation. Gastric gavage was performed on 0, 1, 3, 5, and 7 d with PBS, catechin, and DHKNase*-6*. The mice without DSS induction and gavage with PBS were used as the negative control (PBS), and mice with DSS induction and gavage with PBS or catechin were used as the positive control (DSS, catechin). Two dosages of DHKNase*-6* (1×, 30 mg kg^−1^; 5×, 150 mg kg^−1^) were set up to demonstrate that the therapeutic effect was indeed derived from DHKNase*-6*. Samples were collected after mice sacrifice on the ninth day and well preserved for subsequent investigations. (C) Interrupted weight loss (*n* = 4) for the DHKNase*-6*-treated mice (*n*, numbers of mice; mean ± SEM). (D and E) Better recovered colon length (*n* = 4) after DHKNase*-6* treatment (*n*, numbers of mice; mean ± SEM). After 9-d therapy, the mice colons were collected and photographed. (F) Physiology of the mice colon (*n* = 4) after treatment (*n*, numbers of mice). Tissues were stained with H&E. (G) Damage index (*n* = 4) of colon tissue from the results of (F) (*n*, numbers of biologically independent samples). The damage index was evaluated based on an established method [55]. (H to L) DHKNase*-6* down-regulated the level of IBD characteristic marker MPO, up-regulated the level of growth factors TGF-β1 and VEGF, and down-regulated the IL-1β and TNF-α expression (*n* = 4) in the mice colon (*n*, numbers of biologically independent samples; mean ± SEM). After the mice sacrifice, the colon segments were collected and homogenized immediately, following the centrifuge to remove sediment, and the supernatants were prepared for the ELISA studies. One-way ANOVA test.

After achieving an admirable performance in curing infected wounds as a ROS supplier, we next tried to excavate the application of the dual-action DHKNase-*6* in ROS accumulation disease as a ROS scavenger. IBD, a kind of gastrointestinal tract disorders that couples with prolonged inflammation in the small intestine (Crohn’s disease) or colon [ulcerative colitis (UC)], threatens patients’ life quality, raising the risk of colorectal cancer in UC [55,56]. In particular, excessive accumulation of ROS was one of the leading causes of clinical acute colitis [57–59]. In addition, studies have shown that the pathogenesis of IBD leads to a decrease of the pH value from 7.4 to 5.9 in the colon microenvironment [60–63]. Accordingly, IBD therapy by DHKNase-*6* could be anticipated through scavenging the excessive ROS in the intestine (Fig. 5A). The in vivo ROS scavenging ability of DHKNase*-6* was studied before it was applied to mice. Results showed the ROS response 2′, 7′-dichlorodihydrofluorescein diacetate (DCFH-DA) fluorescence intensity in *Caco-2* cells had been distinctly increased after exogenous ROS incubation and dramatically decreased after the addition of DHKNase*-6* (Fig. S27), implying that DHKNase*-6* could effectively defend cells from high levels of endogenous ROS.

After that, mice were given 5% (w/v) DSS-containing drinking water for 7 d to trigger a colitis colon. Significant weight decrease, severe hematochezia, and intestinal epithelium structure loss of mice following DSS induction were observed (Fig. S28), indicating the successful establishment of mice IBD. Direct pH measurement of intestinal tissues showed pH values greater than 7 even after 7 d of DSS induction (Fig. S29), guaranteeing a suitable working environment for DHKNase*-6* to neutralize ROS. Subsequently, mice were treated with PBS, catechin, and DHKNase*-6* on the predetermined days, with the collection of colon samples 2 d after the final treatment (Fig. 5B). Catechin, a widely studied natural substance for the effective treatment of IBD and showing no bifacial enzyme ability as depicted in Fig. 2A, was employed to provide a therapeutic contrast. After 9 d of therapy, DHKNase-*6* predominantly interfered with weight loss, colon length shortness, and colon damage index increase, while the other formulations exhibited less anti-IBD outcomes. In addition, it was observed that the multi-dose administration of DHKNase*-6* did not cause the accumulation of acidic groups in the colon microenvironment to impair the ROS scavenging function of DHKNase*-6* (Fig. S30). As depicted in Fig. 5C, during the first 2 d of treatment, all mice except those in the PBS group showed a sustained weight loss, while those in the DSS group declined to 89.5%. After 2 d of remedy, mice treated with DHKNase-*6* began to regain weight. On the fourth day, the mice’s body weight in the DHKNase-*6* (5×) group recovered to reach 101.2%, while the body mass of mice within the DSS cohort still continued to decrease to 87%. After 9 d of treatment, colon length in the DSS group still presented a severe reduction to 6.35 cm on average, whereas it was recovered to 8.2 cm on average after DHKNase-*6* (5×) cure (Fig. 5D and E), demonstrating the significant therapy efficacy of DHKNase-*6* for IBD. There was observed intact colonic epithelial structure of regular finger-shaped crypt and goblet cells after DHKNase-*6* (5×) therapy, while the colon in the other treatment groups still showed pathological symptoms such as epithelial structure loss and ulceration (Fig. 5F). The similar colonic damage score compared with the PBS group further indicated the recovery of the colon from a pathological to a physiological state after DHKNase-*6* therapy (Fig. 5G). The IBD-related myeloperoxidase (MPO) and SOD activity had been studied to have a significant rise and were down-regulated by DHKNase-*6* (Fig. 5H and Fig. S31A). On the contrary, the CAT enzyme, which helps the host to clear the excessive accumulation of ROS in the intestine, was up-regulated by DHKNase-*6* treatment (Fig. S31B), demonstrating that DHKNase-*6* significantly increased the antioxidant and ROS scavenging capacity of the intestine in IBD mice. Additionally, ELISA analysis also confirmed that DHKNase-*6* could enhance the levels of growth factors TGF-β1, VEGF, and inflammation-deterrent cytokine IL-10, but decreased the inflammation-propulsive factors expression of IL-1β, IL-6, as well as the TNF-α (Fig. 5I to L and Fig. S32), facilitating the intestinal tissue and blood vessel regeneration and inhibition of inflammation. Beyond that, tail vein injection of high doses of DHKNase-*6* (300 mg kg^−1^) presented no overt toxicodynamics to the major organs of mice, proving the possibility of systemic administration for DHKNase-*6* (Fig. S33). Collectively, DHKNase-*6* showed superior efficacy for colitis therapy attributing to the enhanced ROS clearance, tissue regeneration, and inflammation suppression.

### Regulation of microbiome dysbiosis in the IBD colon

**Fig. 6.**
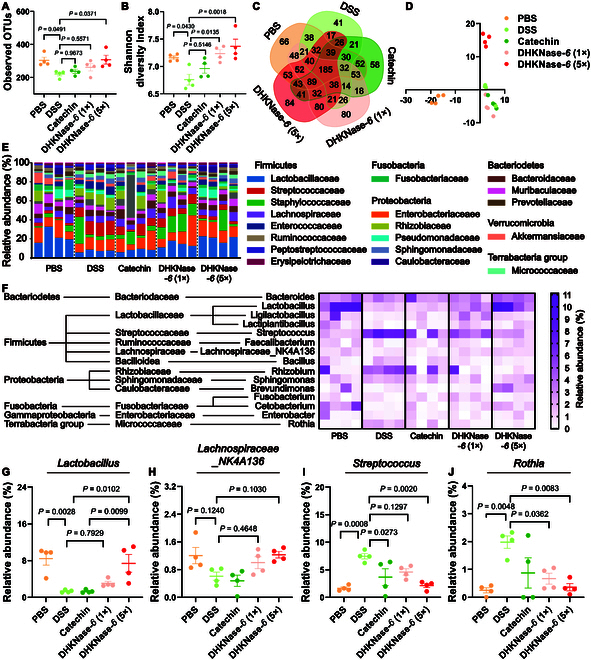
DHKNase*-6* regulated the microbiome community in colitis mice colon. (A to D) DHKNase-*6* improved the relative abundance of the colon (*n* = 4) microbiota concluded from the operational taxonomic units (OTU), Shannon diversity, Venn diagram, and partial least squares discriminant analysis (PLS-DA) maps (*n*, numbers of mice; mean ± SEM). Each dot represented a treatment mouse. On the ninth day after the first treatment, mice were sacrificed and feces were collected to store in an −80 °C refrigerator, dry-ice transported to the testing company for RNA extraction and analysis. (E) At the phylum level, DHKNase-*6* mainly regulated the relative abundance of *Firmicutes*, *Proteobacteria*, and *Fusobacteria* (*n* = 4). Percentage of total sequences was used to describe the taxonomy. Each bar represented a treatment mouse (*n*, numbers of mice). (F) DHKNase-*6* disturbed the *Lactobacillaceae*, *Lachnospiraceae*, *Streptococcaceae*, and *Rhizobiaceae* (*n* = 4) microbiome abundance at the family level. Each column represented a treatment mouse. The abundance was shown as a relative percentage (*n*, numbers of mice). (G to J) DHKNase-*6* regulated the *Lactobacillus*, *Lachnospiraceae_NK4A136*, *Streptococcus*, and *Rothia* (*n* = 4) community in the IBD mice colon. Each dot represented a treatment mouse (*n*, numbers of mice). Data analysis was performed on BMKCloud. One-way ANOVA test.

Increasing evidence indicates that IBD development is associated with the community of the intestinal microbiota, with microbial imbalance potentially leading to metabolic disturbances, mucosal injury, and worsening inflammation. To verify whether DHKNase-*6* could regulate intestinal microbiota dysbiosis, the bowel microbiota composition was investigated with sequencing analysis of RNA 16*S* ribosomal gene in the V3 + V4 regions. As depicted in Fig. 6A and B, both the intestinal flora diversity and abundance in the mice colon were drastically reduced following DSS administration, indicating the direct relevance of microbiota to colitis development. Significant microbiome abundance loss was still observed in the catechin-treated colon, while the most enriched diversity and abundance were observed after the DHKNase-*6* remedy (Fig. 6C and D). At the phylum level, DHKNase*-6* showed the most apparent regulation in *Firmicutes*, *Proteobacteria*, *Fusobacteria*, and *Terrabacteria* groups (Fig. 6E). At the same time, DHKNase-*6* displayed prior regulation on *Lactobacillaceae*, *Lachnospiraceae*, *Streptococcaceae*, and *Rhizobiaceae* (Fig. 6F). Specifically, the abundance of bacteria that assist in anti-inflammation and IBD alleviation including acetate-producing *Cetobacterium* [64], lactate-producing *Lactobacillus* [65] and *Ligilactobacillus* [66], butyrate-producing *Lachnospiraceae_NK4A136* [67], and lipid metabolism-associated *Brevundimonas* [68] was significantly up-regulated by DHKNase-*6* cure (Fig. 6G and H and Fig. S34A to C). In contrast, the abundance of inflammation- and DNA damage-associated *Streptococcus* [69], *Rothia* [70], and *Rhizobium* [71] was down-regulated after DHKNase*-6* (5×) treatment, supporting the resolution of intestinal inflammation due to the reduction of pathogenic bacteria (Fig. 6I and J and Fig. S34D). Together, DHKNase*-6* exhibited modulation of intestinal microbiota dysbiosis by either up-regulating the probiotics abundance or down-regulating the pathogenic bacteria abundance.

### DHKNase-*6* recovered gene expression pattern from inflammation to normalcy

**Fig. 7.**
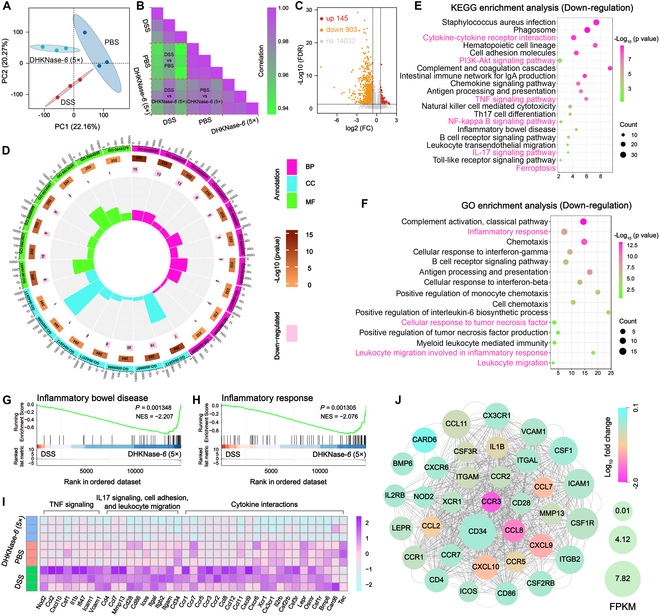
DHKNase*-6* (5×) altered the gene expression in the host BALB/c mice colon. (A and B) The PCA and correlation matrix map (*n* = 3) indicated the expression of genes from inflammation to health in the IBD colon after DHKNase*-6* (5×) treatment. Each dot represented an experimental mouse (*n*, numbers of mice). On the ninth day, mice colon tissues were collected and rinsed off the contents with PBS solution, saved at −80 °C before extraction and analysis. (C and D) DHKNase*-6* (5×) regulated a total of 1,048 DEG expressions (*n* = 3) mainly in BP, CC, and MF aspects (*n*, numbers of mice; mean), in which the cellular pathway expression of chemokine receptor binding (GO:0042379), chemokine receptor activity (GO:0004950), major histocompatibility complex (MHC) class II protein complex (GO:0042613), MHC class II protein complex binding (GO:0023026), leukocyte migration involved in inflammatory response (GO:0002523), collagen type III trimer (GO:0005586), positive regulation of IL-6 biosynthetic process (GO:0045410), C–C chemokine receptor activity (GO:0016493), positive regulation of monocyte chemotaxis (GO:0090026), chemotaxis (GO:0006935), and complement activation of classical pathway (GO:0006958) were significantly down-regulated in DHKNase*-6* (5×) group mice. From the outside to the inside, it is the following: classification (the same color is the same classification), the total gene numbers of the corresponding metabolism with −log_10_ values determined by background color, the numbers of up-regulated genes, and rich factor. (E and F) Differentially recorded Kyoto Encyclopedia of Genes and Genomes (KEGG) and GO metabolic pathways in the mice colon (*n* = 3) after cure compared to the DSS group; each row represented a BP (*n*, numbers of mice). (G and H) DHKNase*-6* (5×) down-regulated the IBD and inflammatory response in the host mice (*n* = 3) colon (*n*, numbers of mice). (I) The significantly down-regulated genes refer to the TNF and IL-17 signaling, cell adhesion, leukocyte migration, and cytokine interactions in the IBD mice gut (*n* = 3) after DHKNase*-6* (5×) therapy. Each row represented a treatment mouse (*n*, numbers of mice). (J) Protein–protein interaction network revealed that the potential target gene CCR3 of DHKNase*-6* (5×) treatment in reversing the inflammatory physiology inside the IBD colon (*n* = 3), for which the CCR3 gene expression was enormously down-regulated by DHKNase*-6* (5×) treatment (*n*, numbers of mice). Cytoscape software (3.9.1) was used to draw the protein–protein interaction network. Data analysis was performed on BMKCloud.

The transition of gene expression from an inflamed state to a normal state within the host’s gut can serve as compelling evidence for the resolution of inflammation. To further disclose the DHKNase*-6*-related anti-inflammation mechanism relative to gene regulation, colons were harvested for transcriptomics analysis. By reducing the transcription and expression of inflammatory signaling pathways in the host intestine, DHKNase*-6* validated the resolution of inflammation physiology. It exhibited the significant different gene expression between the DHKNase*-6* and DSS group in unguided principal components analysis (PCA) (Fig. 7A), ascertaining the virtually regulated gene expression in the colitis colon by DHKNase*-6*. DSS treatment significantly altered gene expression in the gut (Fig. 7B and Figs. S35 and S36), while it was regulated more closely to the healthy colon after DHKNase-*6* therapy (Fig. S37). For the diverse gene expressions, 1,048 differentially expressed genes (DEGs) were observed between the DSS and DHKNase*-6* group, of which 903 genes were down-regulated after DHKNase*-6* administration (Fig. 7C). It was further identified that the DEGs after DSS induction were mainly focused on complement activation, inflammatory response, cytokine expression, and leukocyte migration according to the gene ontology (GO) annotation in aspects of biological process (BP), cellular component (CC), and molecular function (MF) (Fig. 7D and Fig. S38), which exacerbated the inflammatory response in the host intestine. On the contrary, the inflammation response signaling was down-regulated to approach normalcy after DHKNase-*6* therapy (Fig. 7E and Fig. S39). Typically, the cytokine–cytokine receptor interaction, phosphatidylinositol 3-kinase (PI3K)–Akt, tumor necrosis factor (TNF), nuclear factor κB (NF-κB), and IL-17 signaling pathways and ferroptosis were drastically inhibited by DHKNase-6. In contrast to that, the inflammatory response, cellular response to TNF, leukocyte migration involved in inflammatory response, and leukocyte migration were up-regulated following the DSS induction, yet distinctly down-regulated after DHKNase*-6* administration (Fig. 7F and Fig. S40).

In addition, gene signatures by the gene set enrichment analysis (GSEA) indicated that a series of BPs including IBD, inflammatory response, chemotaxis, and chemokine signaling pathways were clearly down-regulated by the DHKNase*-6* therapy (Fig. 7G and H and Fig. S41A and B). Apart from the IBD and chemokine signaling pathways, immune and defense response gene transcription were found to be up-regulated after DSS feeding (Fig. S41C to F), which could be related to the pathogenic bacteria proliferation and relevant autoimmune initiation triggered by the disturbed intestinal flora community following the colitis emergence. Especially, the transcription level of the crucial genes that referred to the cytokine–cytokine receptor interaction pathway, IL-17 signaling, and TNF signaling in the colons were efficiently down-regulated to resemble more closely to the healthy one beyond dispute after DHKNase-*6* cure (Fig. 7I and Figs. S42 to S45). In addition, the potential target gene for DHKNase-*6* to shut colitis was screened as CCR3, which was ultimately most down-regulated after DHKNase-*6* treatment (Fig. 7J and Fig. S46).

Once CCR3 is activated by CCL11, it initializes the downstream PI3K-Akt [72] and NF-κB signaling [73], promoting the up-regulation of IL-1β, IL-6, and TNF-α gene expression [74], facilitating the IL-17 [75] and TNF signaling pathway. The next activation of AP-1 [76] and cEBPβ [77] leads to the up-regulation of CCL2 [78], CXCL10 [79], and CSF1 [80], resulting in the recruitment and activation of more leukocyte cells, exacerbating inflammatory physiology. Consequently, DHKNase-*6* suppresses the CCR3 and avoids inflammatory signaling pathways, relieves oxidative stress in the intestinal microenvironment, and assists the return to normalcy.

Together, these results suggested that DHKNase*-6* cured the colitis by down-regulating the transcription levels of inflammatory physiologies including leukocyte migration, chemotaxis, and pro-inflammation cytokines, in which the down-regulation of the CCR3 gene was of vital importance in anti-inflammation therapy.

## Discussion

In this work, we reported that the proton-dependent DHKNase-*6* was simultaneously equipped with ROS generation and elimination capability for realizing ROS-related disease treatment, including fast infectious wound healing and IBD therapy. It had been identified that multiple enzyme-mimicking DHKNase-*6* could generate highly toxic ROS such as ·O_2_^−^, ^1^O_2_, and ·OH in an atmosphere of pH below 5.6 and was employed to eliminate the pathogenic bacteria for disrupting the bacterial membrane. Also, DHKNase-*6* could significantly lessen the inflammatory response by suppressing pro-inflammatory cytokine expression and neutrophil infiltration, up-regulating anti-inflammatory and growth factors, enhancing early angiogenesis, stimulating collagen deposition, promoting the formation of the dermis, as well as avoiding wound hypothermia, thereby speeding up wound healing.

Alternatively, it has been certified that DHKNase-*6* exhibited adequate ROS scavenging in conditions of pH above 5.6 for effective IBD treatment. The recovered body weight, colon length, colon morphology, and cytokine expression in the IBD mice were both observed following DHKNase-*6* remedy with up-regulated abundance of probiotics including *Cetobacterium*, *Lactobacillus Ligilactobacillus*, and *Lachnospiraceae_NK4A136* and the down-regulated abundance of pathogenic bacteria including *Streptococcus*, *Rothia*, *Brevundimonas,* and *Rhizobium*. In addition, the significantly reduced inflammation and immune response gene expression in the host intestine were also identified after DHKNase-*6* therapy. The transcriptomic analysis confirmed that DHKNase-*6* administration significantly reduced the host immune defense and inflammatory response. More specifically, the recovery of normal metabolism was recognized to be highly related to the CCR3 gene transcription suppression, in which the DHKNase-*6* treatment drastically down-regulates the transcription of the CCR3 gene and the subsequent inflammatory signalings. Correspondingly, the recovered microbiome composition and gene transcription in turn promoted the closure and cure of IBD.

In summary, we reported for the first time that DHKNase-*6* derived from natural flavonoids exhibited double-side ROS regulation for applicative disease therapy. The discovery of natural small-molecule enzyme imitators with this type of DHKN structure has effectively broadened the scope of enzyme substitution research in comparison to nano-artificial enzymes. Considering the benefits of wide sources, good modifiability, excellent biocompatibility, and avoidance of batch-to-batch variability, those natural compounds are expected to be directly used as precursors for downstream drug synthesis and production for treatment of more ROS-related diseases in the future. This work provides a distinguished guiding landmark for developing biomimetic enzymes in the field of natural small molecules with admirable biomedical application potential.

## Materials and Methods

### Statistical analysis

All statistical results were shown as mean ± standard error. Statistical analysis of experimental results was carried out by blind counter. One-way analysis of variance (ANOVA) and Tukey’s post-test were used to analyze the statistical significance of differences between groups.

#### Materials

Unless otherwise stated, all chemicals were bought from commercial suppliers and were used without further purification. Using ultrapure water (resistance >18 MΩ cm) to prepare the experimental solutions. An ultrapure water system from Hunan Kertone Water Treatment Co. was employed. The UV-vis investigations were measured by a UV-vis spectrophotometer (Shimadzu UV-3600Plus, Japan). A thermal imaging camera of FLIR 3C type was purchased from FLIR. The pH measurements were carried out on a Bante 210 pH meter. The DNA extraction assay of the bacterial genome was obtained from Solarbio Co., Ltd. (Beijing, China). Catalase assay kit (A007-1-1), Peroxidase assay kit (A084-2-1), Myeloperoxidase assay kit (A044-1-1), Total Superoxide Dismutase assay kit (A001-1-1), Inhibition and produce superoxide anion assay kit (A052-1-1), and MTT cell proliferation and cytotoxicity assay kit (G020-1-1) were purchased from Nanjing Jiancheng Co., Ltd. (Nanjing, China), and were used directly according to the instructions. Mouse IL-6 (EK206), IL-10 (EK210), IL-1β (EK201B), TNF-alpha (EK282), TGF-β1 (EK981), and VEGF (EK283) ELISA kits were purchased from Multisciences Co., Ltd. (Hangzhou, China), and were used directly according to the instructions. Agarose was obtained from Tsingke Co., Ltd. (Beijing, China). DNA Ladder (TSJ105-100) was obtained from Tsingke Biotech Co., Ltd. (Beijing, China). Fetal bovine serum (FBS) and Minimum Essential Medium α (MEN-α) were obtained from Gibco. Sodium acetate, TMB, Chrysin (98%), Diosmetin (98%), Isorhamnetin (90%), Catechin (99.9%), 6,7-Dihydroxycoumarin (98%), Hypericin (99.9%), Kaempferol (99.9%), 8-HQ, p-Phthalic acid (PTA), β-carotene, Cytochrome C, Calcium chloride, Ammonium molybdate, Propidium iodide (PI), and Sodium citrate were obtained from Macklin Co. Ltd. (Shanghai, China). 9, 10-Diphenylanthracene (DPA) was obtained from Aladin Ltd. (Shanghai, China). DSS (36-50 kDa) was purchased from Shenzhen Regent Biochemical Technology Co., Ltd. (Shenzhen, China). Hoechse33342 and Wogonin (98%) were obtained from Solarbio Co., Ltd. (Beijing, China). Superoxide dismutase (SOD), Emodin (98%), and D-AA were obtained from Shanghai Yuanye Co., Ltd. (Shanghai, China). Dimethyl sulfoxide (DMSO), Copper chloride, Paraformaldehyde, and 2', 7'-Dichlorodihydrofluorescein diacetate (DCFH-DA) were purchased from Sigma-Aldrich (Shanghai). Apigenin (98%) and thiourea (TU) were purchased from Energy Chemical Co., Ltd. (Shanghai, China). Magnesium sulfate, Potassium chloride, Manganese chloride, Sodium chloride, and Isopropanol were obtained from Sinopharm Chemical Reagent Co., Ltd. (Shanghai, China).

#### Detection of ^·^OH, ^1^O_2_, and ^·^O_2_^-^

The liberation of ·OH from hydrogen peroxide (H_2_O_2_) decomposition by DHKNase-*6* was investigated as follows: 1) Measuring the change of the electron spin resonance intensity of the hydroxyl radical scavenger DMPO in aqueous solution; 2) Monitoring the disturbance of the oxidized TMB (oxTMB) absorption after adding the hydroxyl radical scavenger 8-HQ, TU, AA, p-phthalic acid (PTA), and isopropanol. After DMPO is combined with the hydroxyl radical, the peak of its electron spin resonance is greatly enhanced due to the increase in the number of unpaired electrons. AA, isopropanol, and TU will preferentially react with the hydroxyl radicals, resulting in a concentration decrease of oxTMB in the solution and leading to an absorption decline at 652 nm. For the electron spin resonance (ESR) experiment, 50.0 mM DMPO was employed in the reaction solution, and immediately monitored with an ESR system (BURUKE EMXPLUS). To detect the presence of ^1^O_2_, the ^1^O_2_-sensitive reagent DPA and β-carotene were added to the reaction solution, and the solutions were monitored at 355 nm and 652 nm after the reaction, respectively. In addition, the ^·^O_2_^-^-trapping reagent Cytochrome C set to a predetermined gradient concentration was adopted to detect the presence of ^·^O_2_^-^ groups. The final working concentrations were 60.0 μM, 0.3 mM, and 5.0 mM for the DHKNase-*6*, TMB, and H_2_O_2_, respectively. The agents were mixed with HAc-NaAc buffer (0.26 M) and then the mixture was gently shaken and dark bathed in water at 35 °C for 1.5 hours. The absorption peak of the solution was recorded with a UV-vis spectrophotometer (UV 3600, SHIMADZU).

#### Peroxidase-like catalytic activity of DHKNase-*6*

The color change of the solution was photographed at room temperature after 2.0 h of reaction. The steady-state kinetic assay of the DHKNase-6 toward TMB was carried out at 35 °C. The working concentrations of DHKNase-6, H_2_O_2_, and TMB were 60.0 μM, 4.0/5.0 mM, and 0.2/0.3 mM respectively. The reaction time was set to 2.0 h. The 0.26 M of NaAc-HAc (pH 4.0) solution was used as a buffer solution. The UV-vis spectrophotometer was employed to monitor the density of the solutions at 652 nm. The concentration (c) of oxTMB in the solution after the reaction was calculated with the Ramburger equation: Abs=*k*·*b*·*c*— (1) [1];

Where the Abs represents the measured absorbance value of the solution, *ĸ* represents the molar absorbance coefficient of oxTMB at 652 nm (39,000 L M^-1^ cm^-1^), and *c* represents the concentration of oxTMB.Subsequently, the enzyme activity was calculated with the equation: *U*·*t*=*c* — (2);

Where the *U* represents the enzyme activity, which was defined as converting 1μmol per minute, and t represents the time, the results were then used for plotting.

#### ICP-MS

ICP-MS. Accurately weigh about 0.1 g DHKNase-*6* in a 50 ml PTFE digestion tube to dissolve with 5 ml concentrated nitric acid and 1 ml hydrofluoric acid. The solution was poured into a stainless-steel reactor, heating at 190 °C for 10 hours. Then customized the mixture with deionized water to a volume of 25 ml. The standard concentration curve was pointed at 0, 0.5, 1.0, 2.0, and 5.0 mg L^-1^ using national standard material. Subsequently, the standard solution calibration curve was measured by the ICP-OES instrument, and the test results were calculated.

#### Hydrogen peroxide removal

The residual H_2_O_2_ content in the solution was measured by molybdenic acid chromogenic strategy. Briefly, DHKNase-6 was first added to the solution containing H_2_O_2_ and reacted for half an hour at 35 °C. The final reaction concentrations for DHKNase-6 were 10, 20, 40, 60, 80, 100 μM, and 10 mM for H_2_O_2_, respectively. For reactions under acidic conditions, HAc-NaAc buffer (0.26 M, pH 4.0) was selected, while phosphate buffer (10 mM, pH 7.4) was selected for experiments under neutral conditions. After the reaction, the solution was mixed with an equal volume of ammonium molybdenum sulfate (2.4 M) solution and shaken evenly through a vortex oscillator. Then, absorption at 330 nm was measured by a UV spectrophotometer within half an hour.

#### ^·^O_2_^-^ scavenging

The ^·^O_2_^-^ was produced by xanthine oxidase in the process of oxidizing xanthine, which subsequently oxidized hydroxylamine in solution to nitrite, and then nitrite reacted with the color developer for color development. When there was ^·^O_2_^-^ scavenger in the solution, the degree of oxidation of hydroxylamine to nitrite reduced due to the removal of the ^·^O_2_^-^, thereby reducing the degree of color development of the solution. The ^·^O_2_^-^-scavenging capacity of DHKNase-*6* was studied using the Inhibition and production superoxide anion assay kit. All procedures were performed according to the instructions and repeated three times.

#### ^·^OH removal

The Fenton reaction was adopted to liberate ^·^OH, which further oxidized TMB to render a blue color in the solution. When there was ^·^OH scavenger in the solution, the amount of oxTMB produced in the solution decreased. In the experiment, TMB and DHKNase-*6* were added to the HAc-NaAc buffer (0.26 M, pH 6.0) containing Cu^2+^, then H_2_O_2_ was quickly added after shaking evenly, and the absorbance value was measured after 0.5 h incubation at 35 ℃. The working concentrations for Cu^2+^, TMB, and H_2_O_2_ were 1.2 mM, 0.3 mM, and 3.0 mM, respectively.

#### RNS removal

DPPH had been reported as a stable nitrogen radical with stable odd electron nitrogen atoms in its structure, which was commonly used in RNS scavenging studies. In the experiment, DHKNase-*6* was incubated with DPPH, and then water bath-reacted at 35 °C for 0.5 h, and then the absorption value was measured using an ultraviolet spectrophotometer. The monitoring wavelength range was 420 - 620 nm. The final working concentration for DPPH was 114 mM, and 0, 2, 4, 8, 12, 16, and 20 mM for DHKNase-*6*, respectively.

#### Temperature, salt, and pH tolerance

To evaluate DHKNase-*6*’s resistance to extreme environments, the natural catalytic enzyme HRP was selected for comparison. For temperature resistance, DHKNase-*6* and HRP were incubated at different temperatures for half an hour. After the temperature recovered to room temperature, DHKNase-*6* and HRP were added to the TMB-oxTMB colorific system. For salt resistance, DHKNase-*6* and HRP were mixed with different concentrations of Na^+^-containing solutions and incubated at room temperature for half an hour, followed by the catalysis experiment. For pH resistance, DHKNase-*6* and HRP were incubated for 0.5 h at room temperature in different concentrations of HAc-NaAc buffer; then, the pH value was adjusted to be the same as the reaction buffer and added to the reaction solution. The final working concentrations for DHKNase-*6* and HRP were 60 μM and 2 U mL^-1^, respectively.

#### Michaelis–Menten kinetic study

DHKNase-*6*, H_2_O_2_ and TMB were mixed in HAc-NaAc buffer and bathed at 35 °C for 1.5 h. The concentration of DHKNase-*6* was fixed at 60 μM while the concentration of one of the substrates, H_2_O_2_ or TMB was varied, the solution was monitored at 652 nm after the reaction. Calculations of velocity and enzyme activity were conducted as: The reaction velocity was calculated with the equation: *v*∙*t*=*c* — (3);

Where *t* represents the time, and c represents the concentration of oxTMB. The calculated reaction rate *v* and TMB concentration were plotted in the rectangular coordinate system.The kinetic parameters were derived by fitting a linear plot using the double-reciprocal equation: 1/*v*=*K_m_*/(*V_max_*[*S*])+1/*V_max_* — (4);

Here, *v* denotes the corresponding reaction rate, *V_max_* is the maximum reaction rate, [*S*] stands for the substrate concentration, and *K_m_* signifies the Michaelis-Menten constant.

#### Preparation of bacterial solutions

Preparation of Bacterial Solutions. A single colony of Gram-negative *Escherichia coli *(*E. coli*) or Gram-positive *Staphylococcus aureus* (*S. aureus*) cultivated on LB agar was inoculated into 100 mL of LB broth and incubated with shaking at 35 °C for 24 hours. Following this, the bacteria were collected through centrifugation (6000 rpm, 5 min), and then rinsed three times. After discarding the supernatant, the bacteria were re-suspended in a PBS solution and diluted to an optical density of 1.0 at 600 nm (OD_600_ = 1.0).

#### In vitro antibacterial ability of DHKNase-*6*

A colony counting strategy determining the alive CFU numbers was employed to evaluate the anti-bacterial effect of the DHKNase-*6*. For all the agents, the *S. aureus* suspension had an OD_600_ value of 1.0, the *E. coli* suspension had an OD_600_ value of 0.1, the NaAc-HAc buffer for *S. aureus* was 1 mM, and for *E. coli *was 4 mM at pH 4.0, the PBS solution occupied a concentration of 10 mM and a pH of 7.4. The final working concentrations of DHKNase-*6* and H_2_O_2_ were 10 μM and 400 μM. The solution was dark bathed at 35 °C for 0.5 h, and then the mixture was spread on the agar LB culture plate in a UV ultra-clean workbench. After 18 hours of culture, photographs and colony counts were taken. All experiments were repeated three times.

#### Bacterial DNA degradation

Bacterial disruption exposes DNA to an environment containing high-level free radicals, resulting in DNA degradation. Bacterial DNA was extracted and subjected to gel electrophoresis after the treatment. There were three groups: bacteria + H_2_O_2_ + buffer, bacteria + H_2_O_2_ + buffer + DHKNase-*6* (60 μM), bacteria + H_2_O_2_ + buffer + DHKNase-*6* (300 μM). Operations were as follows: Firstly, bacteria were mixed with H_2_O_2_ and DHKNase-*6* in NaAc-HAc buffer (pH 4.0, 0.26 M) and left to incubate at 35 °C for 5 hours. Secondly, the suspensions were centrifuged at 3000 rpm to remove the supernatant and then washed with PBS solution twice. The resulting bacterial pellet was performed with the Bacterial DNA Extraction Kit for DNA extraction according to the instructions. Thirdly, the DNA-containing extract was premixed with Loading buffer, and subsequently spotted in an SDS gel and electrophoresis for half an hour at a fixed voltage of 120 V. Finally, the gel after electrophoresis was developed with a gel-imaging system (ChampGel 5000 plus) and photographed. The final working concentration for H_2_O_2_ was 10 mM and for bacteria suspensions were OD_600_ = 0.4, respectively.

#### Fluorescence staining of live and dead bacteria

Two antibacterial groups were investigated: (a) PBS and (b) DHKNase-*6* + H_2_O_2_ + buffer. Briefly, 1.0 ml cultured bacteria (OD_600_ = 1.0) were centrifuged at 5,000 rpm for 5.0 minutes, then washed three times. The precipitate was subsequently mixed with 200 μL saline, then add the PBS (10 mM, pH = 7.4)/DHKNase-*6*, H_2_O_2_, and NaAc-HAc buffer (1 mM, pH 4.0). The working concentrations for DHKNase-*6* and H_2_O_2_ were 10 μM and 400 μM, respectively. The solution was thoroughly mixed and then incubated in the dark at 35 °C for half an hour, then underwent a 5-min centrifugation at 5,000 rpm, and the precipitate was collected and rinsed three times. The precipitate was next diluted with Hochest 33342 (200 μg ml^-1^) and Propidium Iodide (PI) (200 μg ml^-1^), dark bathed in the ice water for 0.5 h, then washed three times with saline, eventually diluted to a volume of 500 μl. Fluorescence images were captured using a microscope equipped with a fluorescence module.

#### Bacteria morphology

Bacteria morphology. After 0.5 h incubation with DHKNase-*6* and H_2_O_2_ at HAc-NaAc buffer (for *S. aureus*: 1 mM, pH 4.0; for *E. coli*: 4 mM, pH 4.0), the bacteria were deposited onto silicon wafers and immobilized using 4% paraformaldehyde for a duration of four hours at 4 °C. Subsequently, the bacteria underwent gradient ethanol dehydration of 30%,50%,70%,80%,90%, and 100%, with each step lasting 10 minutes. Additional freeze-drying operations were performed to adequately remove intracellular water while preserving intact cell morphology. The dried bacteria were coated with a layer of gold and subsequently visualized using field emission scanning electron microscopy (JSM-IT700HR, Jeol) with an energy spectrum instrument (Xplore 30, Oxford).

#### Bacterial infection on the wound surface

All animal research conducted in this study was approved by the Ethics Committee for Animal Experiments at HUNAN SJALABORATORY ANIMAL CO., LTD (Approval No. SCXK 2021-0002). The number of bacterial residues at the wound center tissue was recorded on LB agar medium. The dish was sealed and cultured at 35 °C for 36 hours. After cultivation, the colony growth was photographed.Wound temperature. Infrared imaging of the wound temperature was taken with an infrared camera every 12 hours. Grasped the mouse’s tail while taking pictures to maintain it in a natural forward position. The camera was suspended 5 cm above the wound and parallel to the wound site. Made sure the wound site was in the center of the camera and took pictures later. The infrared imaging results were analyzed using FLIR Thermal Studio Suite software ©2020.

#### Cytotoxicity

The *Caco-2* cell line was utilized to assess cell viability. The cells were cultured in standard MEM-α medium supplemented with 10% fetal bovine serum (FBS). Then the biotoxicity was estimated by a standard MTT assay. Initially, the cells were seeded in a 96-well plate at a density of approximately 5,000 cells per well, with six parallels for each group. Following a 24-hour incubation in a humidified incubator set at 37 °C with 5% CO_2_, and the wells were washed with PBS (0.01 M, pH = 7.4). Subsequently, the DHKNase-*6* solutions in different concentrations (0, 50, 100, and 500 μM) were added and co-incubated for 24 hours. Following that, MTT was introduced into each well, and the plate was maintained in the incubator for an additional 1.5 hours. Ultimately, the assessment of cell viability was conducted by measuring the absorbance at 450 nm using a microplate reader (BioTek Epoch). The cells without DHKNase-*6* solutions were set as the control, and the cell-free medium with MTT served as the background. Cell viability was determined relative to the cell growth in the control group.

#### Hemolysis assay

The hemolysis test was as follows: after the mice were anesthetized, blood was collected from the orbital venous sinus. The blood was immediately anticoagulated with 3% sodium citrate in a ratio of 4:1 and then centrifuged at 3,000 rpm for five minutes. Subsequently, the precipitate was washed three times and collected. The obtained blood cell solution was mixed with saline in a volume ratio of 4:5. Subsequently, 0.2 ml blood cell was mixed with 5 ml DHKNase-*6* (400 μM), saline, and deionized water at 37 °C for 30 minutes. After another one-hour incubation at 37 °C, the solutions were centrifuged for 5 minutes at a speed of 3,000 rpm. Then the solutions’ absorbance at 545 nm was recorded.

#### Intracellular ROS clearance

The ability of DHKNase-*6* to scavenge ROS within *Caco-2* cells had been studied by exogenous H_2_O_2_-induced endogenous ROS levels rise. The cells were cultured in a standard MEM-α medium supplemented with 10% fetal bovine serum (FBS). Cells were inoculated and adhered to overnight. Then, the culture medium was removed, and the culture plate was washed twice with PBS solution to remove unadherent cells. Next, DCFH-DA-containing serum-free medium was added to incubate cells for half an hour, followed by two rinses with PBS solution. Another half-hour incubation with H_2_O_2_ and DHKNase-*6* in a serum-contained medium was then conducted. The intracellular green fluorescence was monitored at a microscope with a fluorescence module. Cells with only H_2_O_2_ treatment were used as the positive control, while the cells with only serum-contained medium treatment were used as the negative control. The final working concentrations for DHKNase-*6*, H_2_O_2_, and DCFH-DA were 0.5 mM, 1 mM, and 10 mM, respectively.

#### In vivo anti-infection

Male BALB/c mice (6 weeks, 22-25 g) were obtained from HUNAN SJALABORATORY ANIMAL CO., LTD and divided into five groups: PBS (10 mM, pH = 7.4); NaAc-HAc buffer (1 mM, pH = 4.0); H_2_O_2_ (200 μM); DHKNase-*6* (-) (200 μM); and DHKNase-*6* (+) with each group consisted of three mice. After anesthesia, a wound approximately 18.0 mm in diameter was surgically created on the backs of the mice. Subsequently, the wounds were infected with the *S. aureus* suspension with a concentration of 1.0 × 10^7^ CFU mL^−1^. After 1 h infection, therapy formulations were applied to the wound, for which the group settings were the same as depicted in “In vitro antibacterial ability of DHKNase-*6*”, for a total of three treatments every other day. The wounds were infrared and visual light photographed every another 12 hours. Wound size over time was analyzed with the software Image J. In addition, the mice’s body weight was also daily recorded. After the dedication of the mice, wound tissues were collected for analysis. All mice received treatment in accordance with the guidelines set by the Institutional Animal Care and Use Committee. Mouse body tissues that were not used for experiments were discarded in compliance with the approved protocol. Prior to histological analysis, skin tissues were fixed using a 4% paraformaldehyde solution. Histological analysis was performed through H&E staining, while collagen formation assessment was conducted using Masson’s trichrome staining. The wound tissues’ inflammatory factors IL-1β and TNF-α were evaluated using enzyme-linked immunosorbent assay kits strictly following the instructions in the manual.

#### DSS-induced acute colitis

DSS-induced acute colitis. Male BALB/c mice, aged six weeks, were group-housed with four mice per cage and allowed to acclimatize for one week before being included in the study. The mice were then exposed to 5% (w/v) DSS in their drinking water for 7 days, after which they were returned to normal water. Healthy control mice were given only normal water. Then 30, 150 mg kg^−1^ of DHKNase-*6* and 30 mg kg^−1^ of catechin or PBS were given orally to mice on scheduled days. Body weight variations were recorded daily throughout the 16-day experimental duration. Fecal samples were gathered for microbiome analysis on the ninth day. On the concluding day, the mice were euthanized, and the entire colon was collected. The length of the colon was then measured and gently rinsed with physiological saline. A 0.5 cm length of the colon was taken for determining CAT, SOD, and MPO activities and the content of cytokines. The remaining colon tissue sample was preserved for sequencing analysis.

#### Histology staining

For histological assessments, H&E and Masson’s trichrome staining tissue sections were prepared by the Wuhan Pinovi Biotechnology Co., Ltd. For *in vivo* animal physiological examinations, a 1 cm tissue segment was initially fixed by immersing in 4% (v/v) buffered formalin and 70% (v/v) alcohol before being embedded in paraffin. Tissue was then stained with H&E and Masson-staining solution, and subsequently analyzed by CaseViewer 2.4. The number of inflammatory cell infiltrates and the collagen deposition in tissues were analyzed by software Image J. The degree of colonic histological injury was assessed in a blinded manner to avoid observer bias, following established protocols [35]. In brief, colonic damage was gradient scored as follows: 0 for normal; 1 for hyperproliferation, irregular crypts, and goblet cell loss; 2 for mild to moderate crypt loss (10–50%); 3 for severe crypt loss (50-90%); 4 for complete crypt loss with intact surface epithelium; 5 for small- to medium-sized ulcers (<10 crypt widths); 6 for large ulcers (≥10 crypt widths). Inflammatory cell infiltration was separately assessed for the mucosa (0 for normal, 1 for mild, 2 for moderate, 3 for severe), submucosa (0 for normal, 1 for mild to moderate, 2 for severe), and muscle/serosa (0 for normal, 1 for moderate to severe). The scores were combined to deliver a total score ranging from 0 to 12.

#### Enzyme-linked immunosorbent assay (ELISA) analysis

For determining the cytokine concentrations in the biological samples, the skin and colon segments were homogenized (1:10 w/v) in 10 mM phosphate buffer (pH 7.4) using a fast sample grinder (JXFSTPRP-48, Shanghai Jingxin Industrial Development Co., Ltd.). Homogenized samples were centrifuged at 10,000 g (CT15RE, HITACHI) and 4 °C for 10 minutes. The cytokine levels in the obtained supernatants were quantified using ELISA kits and followed the instructions with no further actions. ELISA results were measured by a microplate reader (BioTek Epoch) at the corresponding wavelengths.

#### RNA analysis

RNA analysis. For 16S sequencing, fresh mouse intestinal contents and colon segments were collected, deposited in sterile 1.5 ml test tubes and flash frozen using liquid nitrogen. The tubes were properly packaged and shipped to the Biomarker Co., Ltd. (Beijing, China) for microbiome analyses. The total RNA in mice feces was extracted by standard SDS method, and then sequenced by Biomarker Co., Ltd. Using FLASH (v1.2.11) software, the reads from each sample were spliced to generate the raw Tags sequence data, ensuring a minimum overlap length of 10 bp and a maximum allowable mismatch ratio of 0.2 in the overlapping region. Tags were then filtered at Trimmomatic (v0.33) software with a length shorter than 75% of the tag length following quality control to obtain high-quality Clean Tags. Subsequently, chimeras in Clean Tags were removed with UCHIME (v8.1). Using USEARCH (v10.0) to cluster the sequence at a level of 97% similarity to filter OTUs at a threshold of 0.005% of all the sequence numbers. Sequencing results were analyzed on the BMK Cloud data analysis platform.

For transcriptome analysis, BALB/c mice were categorized and tested as Control, DSS, and DHKNase-*6* treated groups with 3 duplicates, respectively. The colitis mice were treated and sacrificed for colon collection on the ninth day after the first treatment. Total RNA in colonic tissue was extracted by standard kits and the RNA concentration and integrity were assessed with Nanodrop2000 and Agient2100. Library construction and purification of the extracted total RNA were conducted by Hieff NGS Ultima Dual-mode mRNA Library Prep Kit for Illumina (Yeasen Biotechnology (Shanghai) Co., Ltd.) and HieffNGS DNA selection Beads (Yeasen Biotechnology (Shanghai) Co., Ltd.), and sequencing was performed at Biomarker Technology Co., Ltd (Beijing, China) using the Illumina NovaSeq 6000 platform (San Diego).

## Data Availability

All data needed to evaluate the conclusions in the paper are present in the article and/or the Supplementary Materials.

## Supplementary Materials

Supplementary 1 Materials and Methods Figs. S1 to S46 Tables S1 and S2 Art work Art work image
