# Supplementary material for: Natural Flavonoid-Derived Enzyme Mimics DHKNase Balance the Two-Edged Reactive Oxygen Species Function for Wound Healing and Inflammatory Bowel Disease Therapy
Source: Research (Wash D C). 2024 Sep 9;7:0464. doi: 10.34133/research.0464 (PMC11381673; doi:10.34133/research.0464)
Supplement: Supplementary 1 — Materials and Methods Figs. S1 to S46 Tables S1 and S2 Art work Art work image [file research.0464.f1.pdf]

Supplementary Materials for

Natural flavonoids-derived enzyme mimics DHKNase balance the two-edged ROS function for wound healing and inflammatory bowel disease therapy

Guangfu Feng *et al.*

*Corresponding authors: J. Fang, fangjun1973@hunau.edu.cn; H. Jiang, jhmndcn@hunau.edu.cn; X. Luo, jamesluoxingyu@163.com; J. Zhang, zhangjiaheng@zzu.edu.cn

This PDF file includes:

Materials and Methods
Figures. S1 to S46
Table S1 and S2
Legends S1 to S46; Legends for Table S1 and S2

MATERIALS AND METHODS

Materials. Unless otherwise stated, all chemicals were bought from commercial suppliers and were used without further purification. Using ultrapure water (resistance >18 MΩ cm) to prepare the experimental solutions. An ultrapure water system from Hunan Kertone Water Treatment Co. was employed. The UV-vis investigations were measured by a UV-vis spectrophotometer (Shimadzu UV-3600Plus, Japan). A thermal imaging camera of FLIR 3C type was purchased from FLIR. The pH measurements were carried out on a Bante 210 pH meter. The DNA extraction assay of the bacterial genome was obtained from Solarbio Co., Ltd. (Beijing, China). Catalase assay kit (A007-1-1), Peroxidase assay kit (A084-2-1), Myeloperoxidase assay kit (A044-1-1), Total Superoxide Dismutase assay kit (A001-1-1), Inhibition and produce superoxide anion assay kit (A052-1-1), and MTT cell proliferation and cytotoxicity assay kit (G020-1-1) were purchased from Nanjing Jiancheng Co., Ltd. (Nanjing, China), and were used directly according to the instructions. Mouse IL-*6* (EK206), IL-10 (EK210), IL-1β (EK201B), TNF-alpha (EK282), TGF-β1 (EK981), and VEGF (EK283) ELISA kits were purchased from Multisciences Co., Ltd. (Hangzhou, China), and were used directly according to the instructions. Agarose was obtained from Tsingke Co., Ltd. (Beijing, China). DNA Ladder (TSJ105-100) was obtained from Tsingke Biotech Co., Ltd. (Beijing, China). Fetal bovine serum (FBS) and Minimum Essential Medium α (MEN-α) were obtained from Gibco. Sodium acetate, TMB, Chrysin (98%), Diosmetin (98%), Isorhamnetin (90%), Catechin (99.9%), 6,7-Dihydroxycoumarin (98%), Hypericin (99.9%), Kaempferol (99.9%), 8-HQ, p-Phthalic acid (PTA), β-carotene, Cytochrome C, Calcium chloride, Ammonium molybdate, Propidium iodide (PI), and Sodium citrate were obtained from Macklin Co. Ltd. (Shanghai, China). 9, 10-Diphenylanthracene (DPA) was obtained from Aladin Ltd. (Shanghai, China). DSS (36-50 kDa) was purchased from Shenzhen Regent Biochemical Technology Co., Ltd. (Shenzhen, China). Hoechse33342 and Wogonin (98%) were obtained from Solarbio Co., Ltd. (Beijing, China). Superoxide dismutase (SOD), Emodin (98%), and D-AA were obtained from Shanghai Yuanye Co., Ltd. (Shanghai, China). Dimethyl sulfoxide (DMSO), Copper chloride, Paraformaldehyde, and 2', 7'-Dichlorodihydrofluorescein diacetate (DCFH-DA) were purchased from Sigma-Aldrich (Shanghai). Apigenin (98%) and thiourea (TU) were purchased from Energy Chemical Co., Ltd. (Shanghai, China). Magnesium sulfate, Potassium chloride, Manganese chloride, Sodium chloride, and Isopropanol were obtained from Sinopharm Chemical Reagent Co., Ltd. (Shanghai, China).

Detection of ·OH, $^1O_2$, and $·O_2^-$. The liberation of ·OH from hydrogen peroxide (H_2O_2) decomposition by DHKNase-*6* was investigated as follows: 1) Measuring the change of the electron spin resonance intensity of the hydroxyl radical scavenger DMPO in aqueous solution; 2) Monitoring the disturbance of the oxidized TMB (oxTMB) absorption after adding the hydroxyl radical scavenger 8-HQ, TU, AA, p-phthalic acid (PTA), and isopropanol. After DMPO is combined with the hydroxyl radical, the peak of its electron spin resonance is greatly enhanced due to the increase in the number of unpaired electrons. AA, isopropanol, and TU will preferentially react with the hydroxyl radicals, resulting in a concentration decrease of oxTMB in the solution and leading to an absorption decline at 652 nm. For the electron spin resonance (ESR) experiment, 50.0 mM DMPO was employed in the reaction solution, and immediately monitored with an ESR system (BURUKE EMXPLUS). To detect the presence of $^1O_2$, the $^1O_2$-sensitive reagent DPA and β-carotene were added to the reaction solution, and the solutions were monitored at 355 nm and 652 nm after the reaction, respectively. In addition, the $·O_2^-$-trapping reagent Cytochrome C set to a predetermined gradient concentration was adopted to detect the presence of $·O_2^-$ groups. The final working concentrations were 60.0 µM, 0.3 mM, and 5.0 mM for the DHKNase-*6*, TMB, and H_2O_2, respectively. The agents were mixed with HAc-NaAc buffer (0.26 M) and then the mixture was gently shaken and dark bathed in water at 35 °C for 1.5 hours. The absorption peak of the solution was recorded with a UV-vis spectrophotometer (UV 3600, SHIMADZU).

Peroxidase-like Catalytic Activity of DHKNase-*6*. The color change of the solution was photographed at room temperature after 2.0 h of reaction. The steady-state kinetic assay of the DHKNase-*6* toward TMB was carried out at 35 °C. The working concentrations of DHKNase-*6,* H_2O_2, and TMB were 60.0 µM, 4.0/5.0 mM, and 0.2/0.3 mM respectively. The reaction time was set to 2.0 h. The 0.26 M of NaAc-HAc (pH 4.0) solution was used as a buffer solution. The UV-vis spectrophotometer was employed to monitor the density of the solutions at 652 nm.
The concentration (*c*) of oxTMB in the solution after the reaction was calculated with the *Ramburger* equation — (1):
$Abs = k \cdot b \cdot c$ — (1) [1];
Where the *Abs* represents the measured absorbance value of the solution, *κ* represents the molar absorbance coefficient of oxTMB at 652 nm (39,000 L M^{-1} cm^{-1}), and **c** represents the concentration of oxTMB.
Subsequently, the enzyme activity was calculated with the equation — (2):
$U \cdot t = c$ — (2);

Where the *U* represents the enzyme activity, which was defined as converting 1μmol per minute, and *t* represents the time, the results were then used for plotting.

ICP-MS. Accurately weigh about 0.1 g DHKNase-*6* in a 50 ml PTFE digestion tube to dissolve with 5 ml concentrated nitric acid and 1 ml hydrofluoric acid. The solution was poured into a stainless-steel reactor, heating at 190 °C for 10 hours. Then customized the mixture with deionized water to a volume of 25 ml. The standard concentration curve was pointed at 0, 0.5, 1.0, 2.0, and 5.0 mg L^{-1} using national standard material. Subsequently, the standard solution calibration curve was measured by the ICP-OES instrument, and the test results were calculated.

Hydrogen peroxide removal. The residual H_2O_2 content in the solution was measured by molybdenic acid chromogenic strategy. Briefly, DHKNase-*6* was first added to the solution containing H_2O_2 and reacted for half an hour at 35 °C. The final reaction concentrations for DHKNase-*6* were 10, 20, 40, 60, 80, 100 μM, and 10 mM for H_2O_2, respectively. For reactions under acidic conditions, HAc-NaAc buffer (0.26 M, pH 4.0) was selected, while phosphate buffer (10 mM, pH 7.4) was selected for experiments under neutral conditions. After the reaction, the solution was mixed with an equal volume of ammonium molybdenum sulfate (2.4 M) solution and shaken evenly through a vortex oscillator. Then, absorption at 330 nm was measured by a UV spectrophotometer within half an hour.

$\cdot O_2^-$ scavenging. The $\cdot O_2^-$ was produced by xanthine oxidase in the process of oxidizing xanthine, which subsequently oxidized hydroxylamine in solution to nitrite, and then nitrite reacted with the color developer for color development. When there was $\cdot O_2^-$ scavenger in the solution, the degree of oxidation of hydroxylamine to nitrite reduced due to the removal of the $\cdot O_2^-$, thereby reducing the degree of color development of the solution. The $\cdot O_2^-$-scavenging capacity of DHKNase-*6* was studied using the Inhibition and production superoxide anion assay kit. All procedures were performed according to the instructions and repeated three times.

·OH removal. The Fenton reaction was adopted to liberate ·OH, which further oxidized TMB to render a blue color in the solution. When there was ·OH scavenger in the solution, the amount of oxTMB produced in the solution decreased. In the experiment, TMB and DHKNase-*6* were added to the HAc-NaAc buffer (0.26 M, pH 6.0) containing Cu^{2+}, then H_2O_2 was quickly added after shaking evenly, and the absorbance value was measured after 0.5 h incubation at 35 ℃. The working concentrations for Cu^{2+}, TMB, and H_2O_2 were 1.2 mM, 0.3 mM, and 3.0 mM, respectively.

RNS removal. DPPH had been reported as a stable nitrogen radical with stable odd electron nitrogen atoms in its structure, which was commonly used in RNS scavenging studies. In the experiment, DHKNase-*6* was incubated with DPPH, and then water bath-reacted at 35 °C for 0.5 h, and then the absorption value was measured using an ultraviolet spectrophotometer. The monitoring wavelength range was 420 - 620 nm. The final working concentration for DPPH was 114 mM, and 0, 2, 4, 8, 12, 16, and 20 mM for DHKNase-*6*, respectively.

Temperature, salt, and pH tolerance. To evaluate DHKNase-*6*'s resistance to extreme environments, the natural catalytic enzyme HRP was selected for comparison. For temperature resistance, DHKNase-*6* and HRP were incubated at different temperatures for half an hour. After the temperature recovered to room temperature, DHKNase-*6* and HRP were added to the TMB-oxTMB colorific system. For salt resistance, DHKNase-*6* and HRP were mixed with different concentrations of Na^+-containing solutions and incubated at room temperature for half an hour, followed by the catalysis experiment. For pH resistance, DHKNase-*6* and HRP were incubated for 0.5 h at room temperature in different concentrations of HAc-NaAc buffer; then, the pH value was adjusted to be the same as the reaction buffer and added to the reaction solution. The final working concentrations for DHKNase-*6* and HRP were 60 μM and 2 U ml^{-1}, respectively.

Michaelis-Menten kinetic study. DHKNase-*6*, H_2O_2 and TMB were mixed in HAc-NaAc buffer and bathed at 35 °C for 1.5 h. The concentration of DHKNase-*6* was fixed at 60 μM while the concentration of one of the substrates, H_2O_2 or TMB was varied, the solution was monitored at 652 nm after the reaction. Calculations of velocity and enzyme activity were conducted as:
The reaction velocity was calculated with the equation — (3):

$$v \cdot t = c \qquad \text{— (3);}$$

Where *t* represents the time, and *c* represents the concentration of oxTMB. The calculated reaction rate *v* and TMB concentration were plotted in the rectangular coordinate system.
The kinetic parameters were derived by fitting a linear plot using the double-reciprocal equation:

$$\frac{1}{v} = \frac{Km}{Vmax[S]} + \frac{1}{Vmax} \quad — (4);$$

Here, *v* denotes the corresponding reaction rate, *Vmax* is the maximum reaction rate, *[S]* stands for the substrate concentration, and Km signifies the Michaelis-Menten constant.

Preparation of Bacterial Solutions. A single colony of Gram-negative *Escherichia coli* (*E. coli*) or Gram-positive *Staphylococcus aureus* (*S. aureus*) cultivated on LB agar was inoculated into 100 mL of LB broth and incubated with shaking at 35 °C for 24 hours. Following this, the bacteria were collected through centrifugation (6000 rpm, 5 min), and then rinsed three times. After discarding the supernatant, the bacteria were re-suspended in a PBS solution and diluted to an optical density of 1.0 at 600 nm (OD_{600} = 1.0).

***In Vitro* Antibacterial Ability of DHKNase-*6*.** A colony counting strategy determining the alive CFU numbers was employed to evaluate the anti-bacterial effect of the DHKNase-*6*. The plates were set into five segments: (Ⅰ) PBS; (Ⅱ) Buffer; (Ⅲ) H_2O_2; (Ⅳ) DHKNase-*6* + buffer (DHKNase-*6* (-)); (Ⅴ) DHKNase-*6* + Buffer + H_2O_2 (DHKNase-*6* (+)). For all the agents, the *S. aureus* suspension had an OD_{600} value of 1.0, the *E. coli* suspension had an OD_{600} value of 0.1, the NaAc-HAc buffer for *S. aureus* was 1 mM, and for *E. coli* was 4 mM at pH 4.0, the PBS solution occupied a concentration of 10 mM and a pH of 7.4. The final working concentrations of DHKNase-*6* and H_2O_2 were 10 μM and 400 μM. The solution was dark bathed at 35 °C for 0.5 h, and then the mixture was spread on the agar LB culture plate in a UV ultra-clean workbench. After 18 hours of culture, photographs and colony counts were taken. All experiments were repeated three times.

Bacterial DNA degradation. Bacterial disruption exposes DNA to an environment containing high-level free radicals, resulting in DNA degradation. Bacterial DNA was extracted and subjected to gel electrophoresis after the treatment. There were three groups: bacteria + H_2O_2 + buffer, bacteria + H_2O_2 + buffer + DHKNase-*6* (60 μM), bacteria + H_2O_2 + buffer + DHKNase-*6* (300 μM). Operations were as follows: Firstly, bacteria were mixed with H_2O_2 and DHKNase-*6* in NaAc-HAc buffer (pH 4.0, 0.26 M) and left to incubate at 35 °C for 5 hours. Secondly, the suspensions were centrifuged at 3000 rpm to remove the supernatant and then washed with PBS solution twice. The resulting bacterial pellet was performed with the Bacterial DNA Extraction Kit for DNA extraction according to the instructions. Thirdly, the DNA-containing extract was premixed with Loading buffer, and subsequently spotted in an SDS gel and electrophoresis for half an hour at a fixed voltage of 120 V. Finally, the gel after electrophoresis was developed with a gel-imaging system (ChampGel 5000 plus) and photographed. The final working concentration for H_2O_2 was 10 mM and for bacteria suspensions were OD_{600} = 0.4, respectively.

Fluorescence staining of live and dead bacteria. Two antibacterial groups were investigated: (a) PBS and (b) DHKNase-*6* + H_2O_2 + buffer. Briefly, 1.0 ml cultured bacteria (OD_{600} = 1.0) were centrifuged at 5,000 rpm for 5.0 minutes, then washed three times. The precipitate was subsequently mixed with 200 μL saline, then add the PBS (10 mM, pH = 7.4)/DHKNase-*6*, H_2O_2, and NaAc-HAc buffer (1 mM, pH 4.0). The working concentrations for DHKNase-*6* and H_2O_2 were 10 μM and 400 μM, respectively. The solution was thoroughly mixed and then incubated in the dark at 35 °C for half an hour, then underwent a 5-min centrifugation at 5,000 rpm, and the precipitate was collected and rinsed three times. The precipitate was next diluted with Hochest 33342 (200 μg ml^{-1}) and Propidium Iodide (PI) (200 μg ml^{-1}), dark bathed in the ice water for 0.5 h, then washed three times with saline, eventually diluted to a volume of 500 μl. Fluorescence images were captured using a microscope equipped with a fluorescence module.

Bacteria morphology. After 0.5 h incubation with DHKNase-*6* and H_2O_2 at HAc-NaAc buffer (for *S. aureus*: 1 mM, pH 4.0; for *E. coli*: 4 mM, pH 4.0), the bacteria were deposited onto silicon wafers and immobilized using 4% paraformaldehyde for a duration of four hours at 4 °C. Subsequently, the bacteria underwent gradient ethanol dehydration of 30%,50%,70%,80%,90%, and 100%, with each step lasting 10 minutes. Additional freeze-drying operations were performed to adequately remove intracellular water while preserving intact cell morphology. The dried bacteria were coated with a layer of gold and subsequently visualized using field emission scanning electron microscopy (JSM-IT700HR, Jeol) with an energy spectrum instrument (Xplore 30, Oxford).

Bacterial infection on the wound surface. All animal research conducted in this study was approved by the Ethics Committee for Animal Experiments at HUNAN SJALABORATORY ANIMAL CO., LTD (Approval No. SCXK 2021-0002). The number of bacterial residues at the wound center tissue was recorded on LB agar medium. The dish was sealed and cultured at 35 °C for 36 hours. After cultivation, the colony growth was photographed.

Wound temperature. Infrared imaging of the wound temperature was taken with an infrared camera every 12 hours. Grasped the mouse's tail while taking pictures to maintain it in a natural forward position. The camera was suspended 5 cm above the wound and parallel to the wound site. Made sure the wound site was in the center of the camera and took pictures later. The infrared imaging results were analyzed using FLIR Thermal Studio Suite software ©2020.

Cytotoxicity. The *Caco-2* cell line was utilized to assess cell viability. The cells were cultured in standard MEM-α medium supplemented with 10% fetal bovine serum (FBS). Then the biotoxicity was estimated by a standard MTT assay. Initially, the cells were seeded in a 96-well plate at a density of approximately 5,000 cells per well, with six parallels for each group. Following a 24-hour incubation in a humidified incubator set at 37 °C with 5% CO_2, and the wells were washed with PBS (0.01 M, pH = 7.4). Subsequently, the DHKNase-*6* solutions in different concentrations (0, 50, 100, and 500 μM) were added and co-incubated for 24 hours. Following that, MTT was introduced into each well, and the plate was maintained in the incubator for an additional 1.5 hours. Ultimately, the assessment of cell viability was conducted by measuring the absorbance at 450 nm using a microplate reader (BioTek Epoch). The cells without DHKNase-*6* solutions were set as the control, and the cell-free medium with MTT served as the background. Cell viability was determined relative to the cell growth in the control group.

Hemolysis assay. The hemolysis test was as follows: after the mice were anesthetized, blood was collected from the orbital venous sinus. The blood was immediately anticoagulated with 3% sodium citrate in a ratio of 4:1 and then centrifuged at 3,000 rpm for five minutes. Subsequently, the precipitate was washed three times and collected. The obtained blood cell solution was mixed with saline in a volume ratio of 4:5. Subsequently, 0.2 ml blood cell was mixed with 5 ml DHKNase-*6* (400 μM), saline, and deionized water at 37 °C for 30 minutes. After another one-hour incubation at 37 °C, the solutions were centrifuged for 5 minutes at a speed of 3,000 rpm. Then the solutions' absorbance at 545 nm was recorded.

Intracellular ROS clearance. The ability of DHKNase-*6* to scavenge ROS within *Caco-2* cells had been studied by exogenous H_2O_2-induced endogenous ROS levels rise. The cells were cultured in a standard MEM-α medium supplemented with 10% fetal bovine serum (FBS). Cells were inoculated and adhered to overnight. Then, the culture medium was removed, and the culture plate was washed twice with PBS solution to remove unadherent cells. Next, DCFH-DA-containing serum-free medium was added to incubate cells for half an hour, followed by two rinses with PBS solution. Another half-hour incubation with H_2O_2 and DHKNase-*6* in a serum-contained medium was then conducted. The intracellular green fluorescence was monitored at a microscope with a fluorescence module. Cells with only H_2O_2 treatment were used as the positive control, while the cells with only serum-contained medium treatment were used as the negative control. The final working concentrations for DHKNase-*6,* H_2O_2, and DCFH-DA were 0.5 mM, 1 mM, and 10 mM, respectively.

In vivo* Anti-infection.** Male BALB/c mice (6 weeks, 22-25 g) were obtained from HUNAN SJALABORATORY ANIMAL CO., LTD and divided into five groups: PBS (10 mM, pH = 7.4); NaAc-HAc buffer (1 mM, pH = 4.0); H_2O_2 (200 μM); DHKNase-*6* (-) (200 μM); and DHKNase-*6* (+) with each group consisted of three mice. After anesthesia, a wound approximately 18.0 mm in diameter was surgically created on the backs of the mice. Subsequently, the wounds were infected with the *S. aureus* suspension with a concentration of 1.0×10^7 CFU mL^{-1}. After 1 h infection, therapy formulations were applied to the wound, for which the group settings were the same as depicted in "In Vitro* Antibacterial Ability of DHKNase-*6***", for a total of three treatments every other day. The wounds were infrared and visual light photographed every another 12 hours. Wound size over time was analyzed with the software Image J. In addition, the mice's body weight was also daily recorded. After the dedication of the mice, wound tissues were collected for analysis. All mice received treatment in accordance with the guidelines set by the Institutional Animal Care and Use Committee. Mouse body tissues that were not used for experiments were discarded in compliance with the approved protocol. Prior to histological analysis, skin tissues were fixed using a 4% paraformaldehyde solution. Histological analysis was performed through H&E staining, while collagen formation assessment was conducted using Masson's trichrome staining. The wound tissues' inflammatory factors IL-1β and TNF-α were evaluated using enzyme-linked immunosorbent assay kits strictly following the instructions in the manual.

DSS-induced acute colitis. Male BALB/c mice, aged six weeks, were group-housed with four mice per cage and allowed to acclimatize for one week before being included in the study. The mice were then exposed to 5% (w/v) DSS in their drinking water for 7 days, after which they were returned to normal water. Healthy control mice were given only normal water. Then 30, 150 mg kg^{-1} of DHKNase-*6* and 30 mg kg^{-1} of catechin or PBS were given orally to mice on scheduled days. Body weight variations were recorded daily throughout the 16-day experimental duration. Fecal

samples were gathered for microbiome analysis on the ninth day. On the concluding day, the mice were euthanized, and the entire colon was collected. The length of the colon was then measured and gently rinsed with physiological saline. A 0.5 cm length of the colon was taken for determining CAT, SOD, and MPO activities and the content of cytokines. The remaining colon tissue sample was preserved for sequencing analysis.

Histology staining. For histological assessments, H&E and Masson's trichrome staining tissue sections were prepared by the Wuhan Pinovi Biotechnology Co., Ltd. For in vivo animal physiological examinations, a 1 cm tissue segment was initially fixed by immersing in 4% (v/v) buffered formalin and 70% (v/v) alcohol before being embedded in paraffin. Tissue was then stained with H&E and Masson-staining solution, and subsequently analyzed by CaseViewer 2.4. The number of inflammatory cell infiltrates and the collagen deposition in tissues were analyzed by software Image J. The degree of colonic histological injury was assessed in a blinded manner to avoid observer bias, following established protocols [35]. In brief, colonic damage was gradient scored as follows: 0 for normal; 1 for hyperproliferation, irregular crypts, and goblet cell loss; 2 for mild to moderate crypt loss (10–50%); 3 for severe crypt loss (50-90%); 4 for complete crypt loss with intact surface epithelium; 5 for small- to medium-sized ulcers (<10 crypt widths); 6 for large ulcers (⩾10 crypt widths). Inflammatory cell infiltration was separately assessed for the mucosa (0 for normal, 1 for mild, 2 for moderate, 3 for severe), submucosa (0 for normal, 1 for mild to moderate, 2 for severe), and muscle/serosa (0 for normal, 1 for moderate to severe). The scores were combined to deliver a total score ranging from 0 to 12.

Enzyme-linked immunosorbent assay (ELISA) analysis. For determining the cytokine concentrations in the biological samples, the skin and colon segments were homogenized (1:10 w/v) in 10 mM phosphate buffer (pH 7.4) using a fast sample grinder (JXFSTPRP-48, Shanghai Jingxin Industrial Development Co., Ltd.). Homogenized samples were centrifuged at 10,000 g (CT15RE, HITACHI) and 4 °C for 10 minutes. The cytokine levels in the obtained supernatants were quantified using ELISA kits and followed the instructions with no further actions. ELISA results were measured by a microplate reader (BioTek Epoch) at the corresponding wavelengths.

RNA analysis. For 16S sequencing, fresh mouse intestinal contents and colon segments were collected, deposited in sterile 1.5 ml test tubes and flash frozen using liquid nitrogen. The tubes were properly packaged and shipped to the Biomarker Co., Ltd. (Beijing, China) for microbiome analyses. The total RNA in mice feces was extracted by standard SDS method, and then sequenced by Biomarker Co., Ltd. Using FLASH (v1.2.11) software, the reads from each sample were spliced to generate the raw Tags sequence data, ensuring a minimum overlap length of 10 bp and a maximum allowable mismatch ratio of 0.2 in the overlapping region. Tags were then filtered at Trimmomatic (v0.33) software with a length shorter than 75% of the tag length following quality control to obtain high-quality Clean Tags. Subsequently, chimeras in Clean Tags were removed with UCHIME (v8.1). Using USEARCH (v10.0) to cluster the sequence at a level of 97% similarity to filter OTUs at a threshold of 0.005% of all the sequence numbers. Sequencing results were analyzed on the BMK Cloud data analysis platform.
For transcriptome analysis, BALB/c mice were categorized and tested as Control, DSS, and DHKNase-*6* treated groups with 3 duplicates, respectively. The colitis mice were treated and sacrificed for colon collection on the ninth day after the first treatment. Total RNA in colonic tissue was extracted by standard kits and the RNA concentration and integrity were assessed with Nanodrop2000 and Agient2100. Library construction and purification of the extracted total RNA were conducted by Hieff NGS Ultima Dual-mode mRNA Library Prep Kit for Illumina (Yeasen Biotechnology (Shanghai) Co., Ltd.) and HieffNGS DNA selection Beads (Yeasen Biotechnology (Shanghai) Co., Ltd.), and sequencing was performed at Biomarker Technology Co., Ltd (Beijing, China) using the Illumina NovaSeq 6000 platform (San Diego).

Statistical analysis. All statistical results were shown as mean ± Standard Error. Statistical analysis of experimental results was carried out by blind counter. One-way ANOVA and Tukey's post-test were used to analyze the statistical significance of differences between groups.

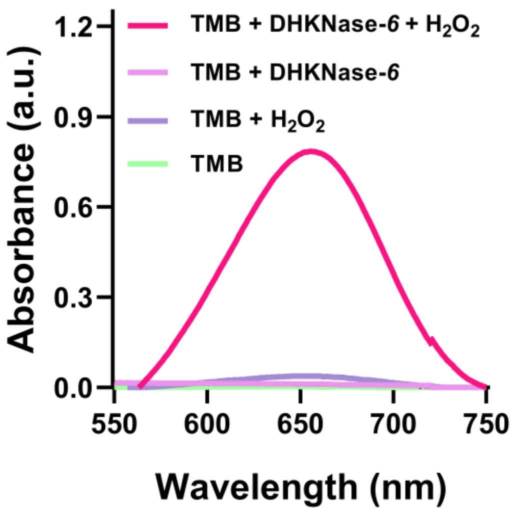

Figure S1. DHKNase-*6* to mimic enzyme catalysis through the decomposition of H_2O_2 and the consequent oxidation of TMB. TMB, 0.3 mM; DHKNase-*6*, 60 μM; H_2O_2, 5 mM; 35 °C incubation for 1.5 h in the NaAc-HAc buffer (pH 4.0), UV-vis spectrophotometer for the absorption measurement in the wavelength range of 550-750 nm. Results were from three independent experiments, mean.

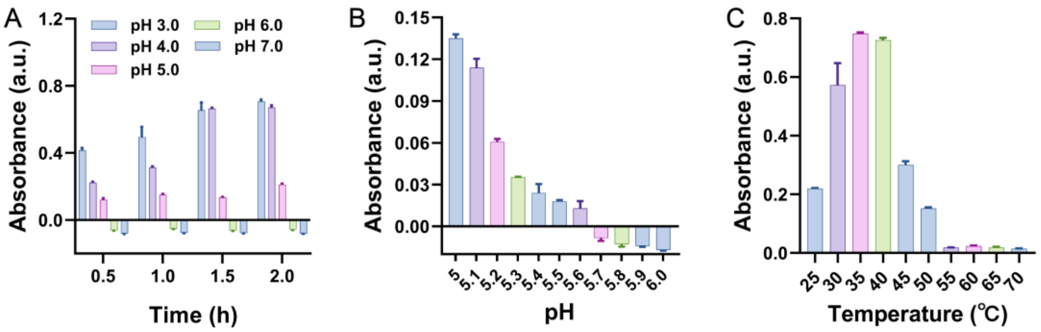

Figure S2. Optimization of catalytic conditions. **(A and B)** Optimized reaction time and pH-responsive threshold for DHKNase-*6*, focusing on reaction time of 1.5 h and threshold of pH 5.6. Once the environment pH exceeded 5.6, DHKNase-*6* showed the opposite ROS removal ability, rather than the ROS supplement. The monitored absorbance subtracted the spontaneous TMB oxidation value due to H_2O_2 decomposition, then the data were used for plotting. **(C)** The optimal reaction temperature for DHKNase-*6* catalytic reaction was defined as 35 °C. TMB, 0.3 mM; DHKNase-*6*, 60 μM; H_2O_2, 5 mM; NaAc-HAc buffer (pH 4.0); absorption at 652 nm was monitored. Results were from three independent experiments; mean ± SEM.

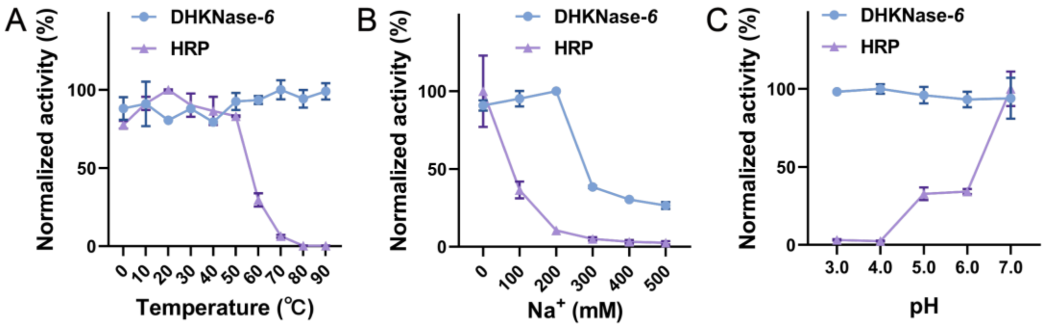

Figure S3. Better environmental resistance of DHKNase-*6* compared to HRP. **(A)** After HRP and DHKNase-*6* were beforehand incubated at different temperatures for half an hour, it was found that the catalytic efficiency of DHKNase-*6* would not lose significantly, whereas the HRP's catalytic ability was greatly lost when the temperature exceeded 50 °C. **(B)** Compared to HRP, the DHKNase-*6* could maintain catalytic ability even in solution with high-concentration Na^+, indicating its better salt tolerance capacity. **(C)** The unchanged oxTMB absorption intensity suggested that the DHKNase-*6* had better acid resistance than HRP. DHKNase-*6*, 60 μM; HRP, 0.1 U; NaAc-HAc buffer (pH 4.0) for DHKNase-*6*; PBS buffer (pH 7.4) for HRP; reaction time for 1.5 h. Results were from three independent experiments; mean ± SEM.

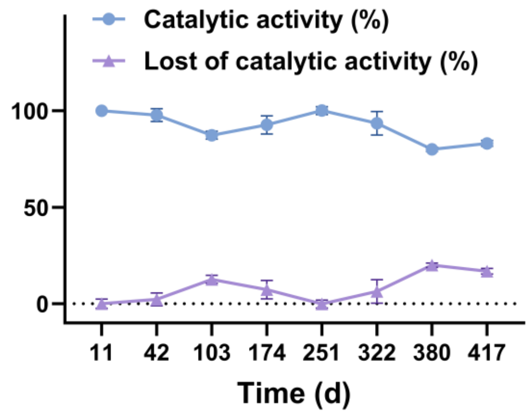

Figure S4. The activity loss of DHKNase-*6* against storage time. After 417 days, the activity of DHKNase-*6* could be maintained at a level over 80%. TMB, 0.3 mM; DHKNase-*6*, 60 μM; H_2O_2, 5 mM; 35 °C incubation for 1.5 h in the NaAc-HAc buffer (pH 4.0). Results were from three independent experiments; mean ± SEM.

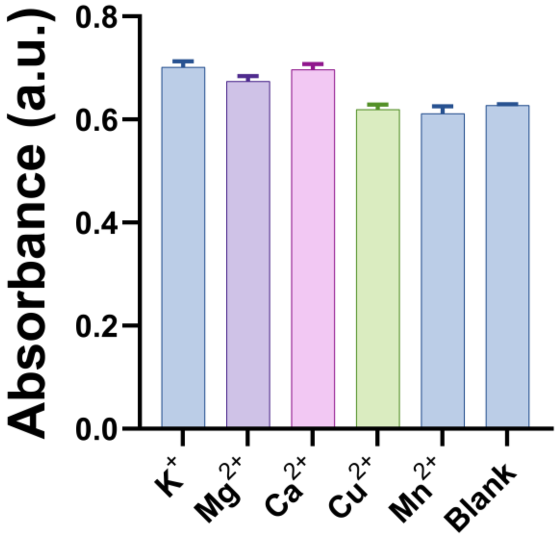

Figure S5. After the addition of the equivalent K^+, Mg^{2+}, Ca^{2+}, Cu^{2+}, and Mn^{2+} content in **Table. S1** to the reaction solution, the absorbance was barely changed, indicating that enzyme mimic ability came from DHKNase-*6* rather than the metal irons. K^+, 0.0005 w/w(%); Mg^{2+}, 0.0005 w/w(%); Ca^{2+}, 0.0017 w/w(%); Cu^{2+}, 0.0005 w/w(%); Mn^{2+}, 0.0005 w/w(%). TMB, 0.3 mM; DHKNase-*6*, 60 μM; H_2O_2, 5 mM; 35 °C, 1.5 h, (pH 4.0). Results were from three independent experiments; mean ± SEM.

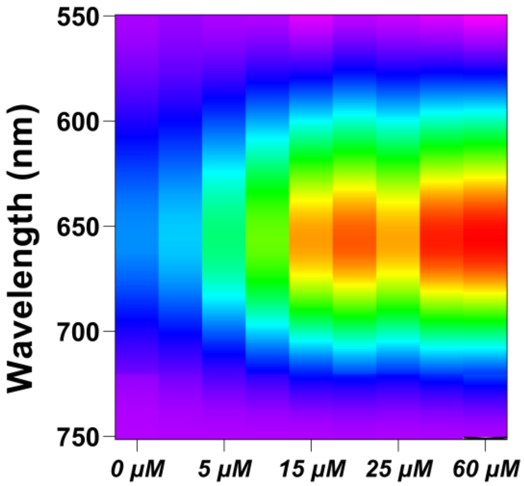

Figure S6. The oxTMB absorption increased with increasing DHKNase-*6* concentration. The absorbance of the reaction solution after addition of DHKNase-*6* (0 μM, 2.5 μM, 5 μM, 10 μM, 15 μM, 20 μM, 25 μM, 40 μM, and 60 μM) demonstrated that the total amount of catalytic product oxTMB increased with the elevated DHKNase-*6* concentration, indicating the catalytic ability was positively correlated with the DHKNase-*6*. H_2O_2, 5 mM; TMB, 0.3 mM; pH 4.0; 1.5 h; 35 °C. Results were from three independent experiments, mean.

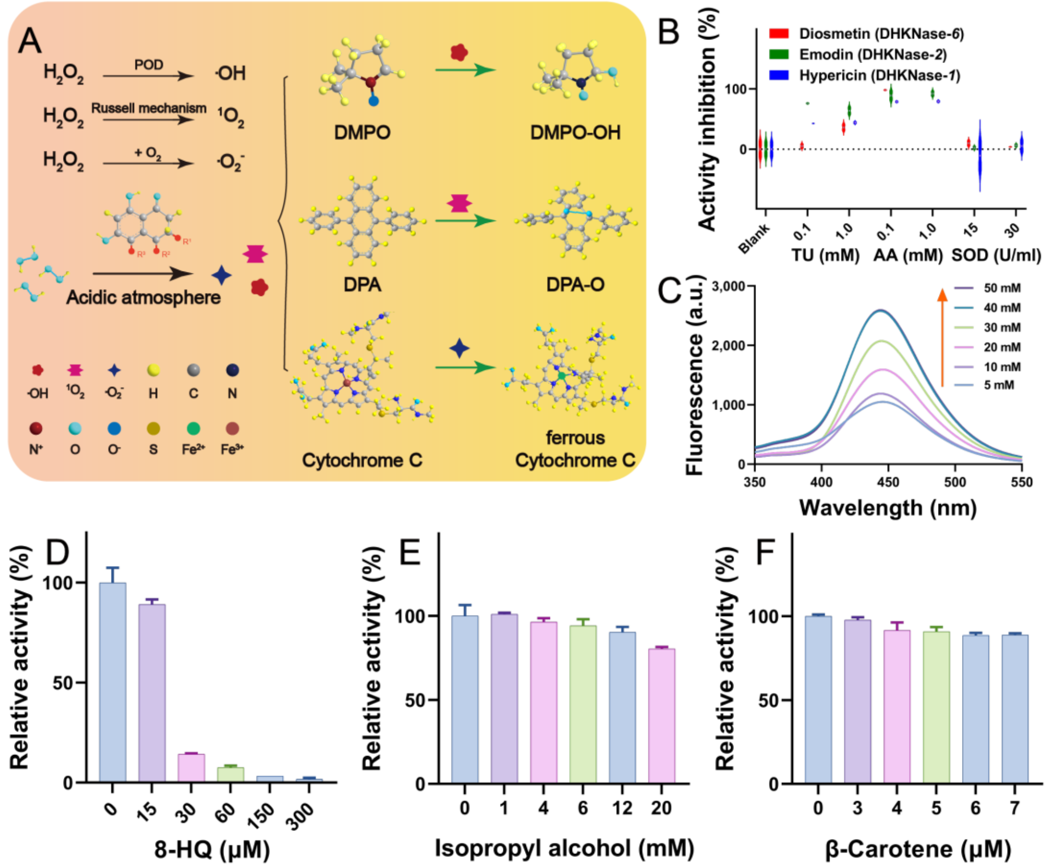

Figure S7. DHKNase to mimic enzyme catalysis and liberate high toxic free radicals. (A) Carton illustration of the enzyme mimic capability for free radicals' conversion of DHKNase (left), and the free radical's detection (right). **(B)** Effects of free radical scavengers TU, AA, and SOD on the TMB-H_2O_2-DHKNase chromogenic reaction, implying the large amount of ·OH and a small amount of $·O_2^-$ in the DHKNase-*6* catalysis. TU and AA were acknowledged as ·OH scavengers. **(C-E)** The increased PTA-OH fluorescence intensity and the decreased oxTMB absorbance after the addition of 8-HQ and isopropyl alcohol verified the ·OH liberation during the reaction, respectively. PTA, 8-HQ, and isopropyl alcohol were known as ·OH scavengers. **(F)** The declined oxTMB absorbance after the addition of the $^1O_2$ scavenger β-Carotene verified the $^1O_2$ liberation during the reaction. H_2O_2, 5 mM; TMB, 0.3 mM; DHKNase-*6*, 60 μM; pH 4.0; 1.5 h; 35 °C. Results were from three independent experiments; mean ± SEM.

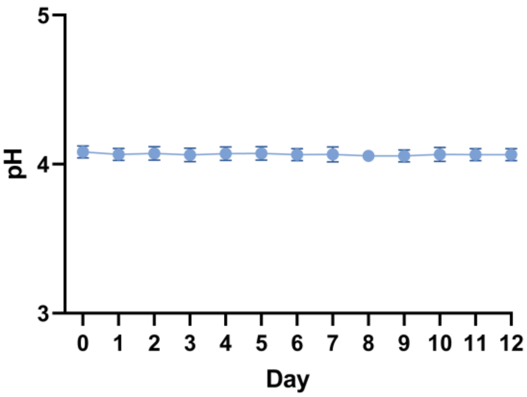

Figure S8. After twelve days and three doses of continuous catalytic, DHKNase-6 demonstrated no impact on environment pH. H_2O_2, 5 mM; DHKNase-*6*, 60 μM; pH 4.0; room temperature. Results were from three independent experiments; mean ± SEM.

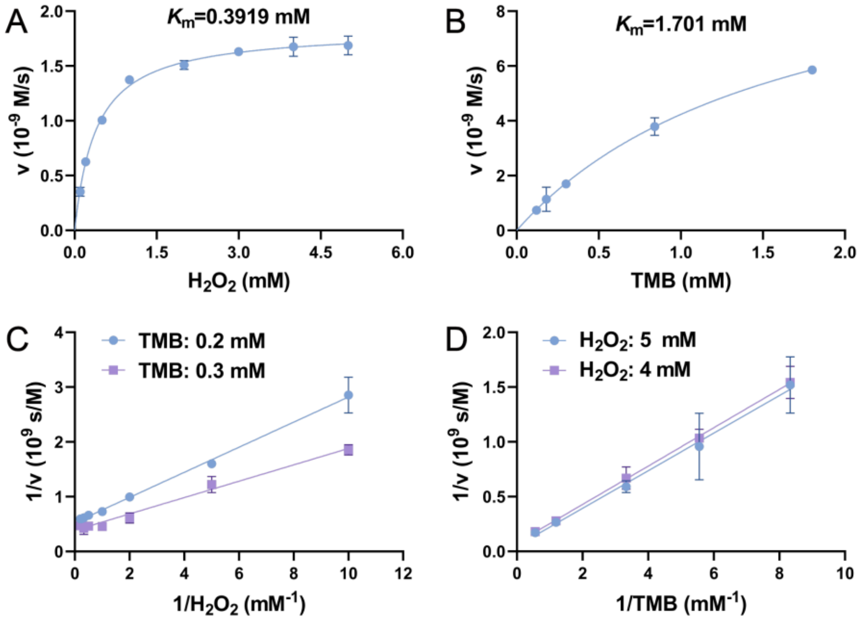

Figure S9. Steady-state kinetic assay of DHKNase-*6*. (A-D) The velocity (*v*) of the reaction was measured using 60 μM DHKNase-*6* in HAc-NaAc buffer (pH 4.0), 35 °C with controlled TMB concentration or H_2O_2 concentration. Error bars indicate the standard deviations of three independent measurements, mean ± SEM. The concentration of TMB was 0.2 or 0.3 mM (**a**, **b**), and the H_2O_2 concentration was varied. The concentration of H_2O_2 was 4.0 or 5.0 mM (**c**, **d**), and TMB concentration was varied. Double reciprocal plots were calculated and plotted between the reaction velocity and the substrate (TMB or H_2O_2) concentration.

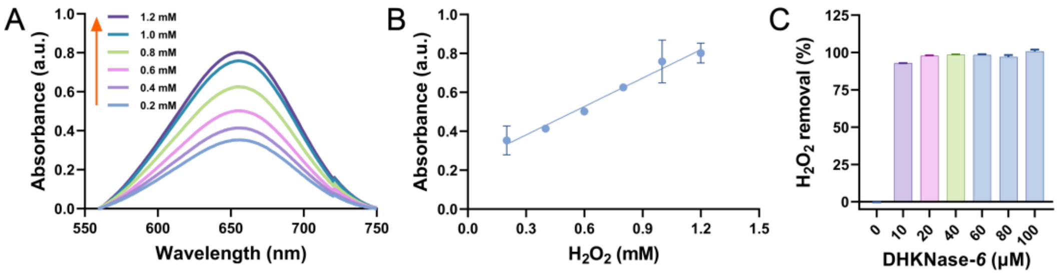

Figure S10. H_2O_2 sensing by DHKNase-*6*. (A and B) The oxTMB absorbance in the solution, which increased linearly with H_2O_2 concentration, could be used to detect H_2O_2 in the environment quantitatively. TMB, 0.3 mM; DHKNase-*6*, 60 μM; pH 4.0; 1.5 h; 35 °C. **(C)** The efficient H_2O_2 decomposition by DHKNase-*6* after the catalysis. The residual H_2O_2 content in the solution was measured by molybdenic acid chromogenic strategy, in which the solution was measured at 330 nm by a UV spectrophotometer. Ammonium molybdenum sulfate, 2.4 M; TMB, 0.3 mM; pH 4.0; 1.5 h; 35 °C. Results were from three independent experiments; mean ± SEM.

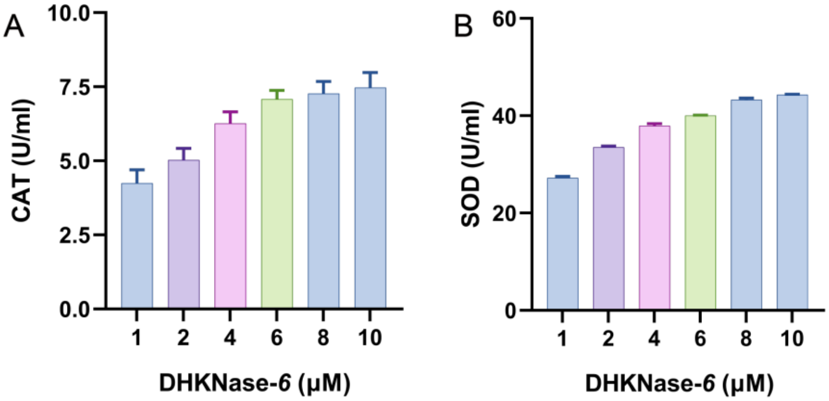

Figure S11. (A and B) The CAT- and SOD-like activity of DHKNase-*6* measured by the assay kit. Results were from three independent experiments; mean ± SEM. Graphs were from three independent results.

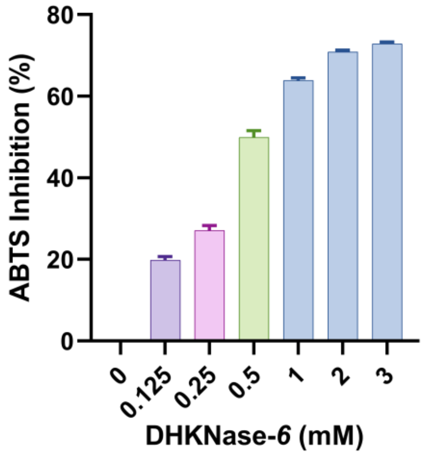

Figure S12. ABTS inhibition by DHKNase-*6.* Briefly, 0.2 ml of ABTS (7.4 mM) was mixed with 0.2 ml of potassium persulfate (2.6 mM) and incubated for 4 h at room temperature in the dark to prepare the working solution. DHKNase-*6* was subsequently mixed with the working solution and incubated at 35 °C for 30 minutes. Then the 734 nm absorbance was monitored.

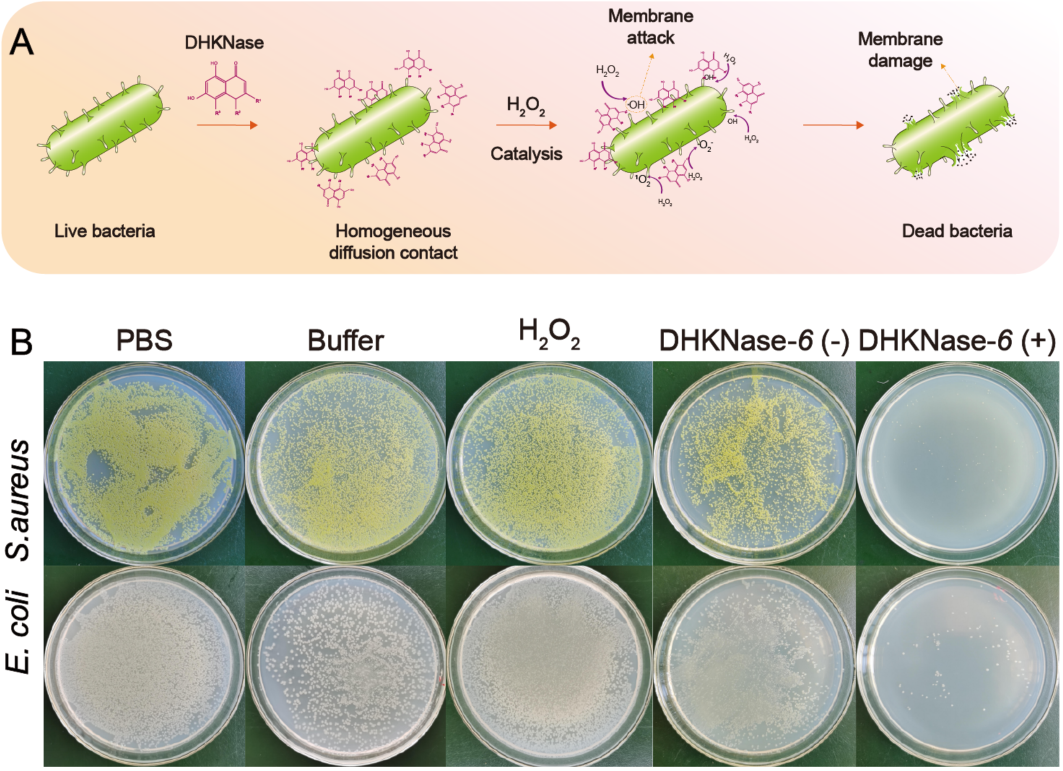

Figure S13. DHKNase-*6* supplied free radicals to wipe out pathogenic bacteria in a low H_2O_2-concentration atmosphere. (A) Scheme of the dynamic sterilization by DHKNase-*6*-mediated membrane disruption. **(B)** The plate counting strategy determined the anti-bacterial capacity of DHKNase-*6* (+). DHKNase-*6* (-): DHKNase-*6* + buffer; DHKNase-*6* (+): DHKNase-*6* + buffer + H_2O_2. DHKNase-*6*, 10 μM; H_2O_2, 400 μM; pH 4.0; 35 °C; 0.5 h. The NaAc-HAc buffer for *S. aureus* was 1 mM, and for *E. coli* was 4 mM. The volume ratio of the total reaction solution to the *S. aureus* (OD_{600} = 1.0) or *E. coli* (OD_{600} = 0.1) suspension was 20:1. Photographs were taken after 18 hours of culture. All experiments were repeated three times.

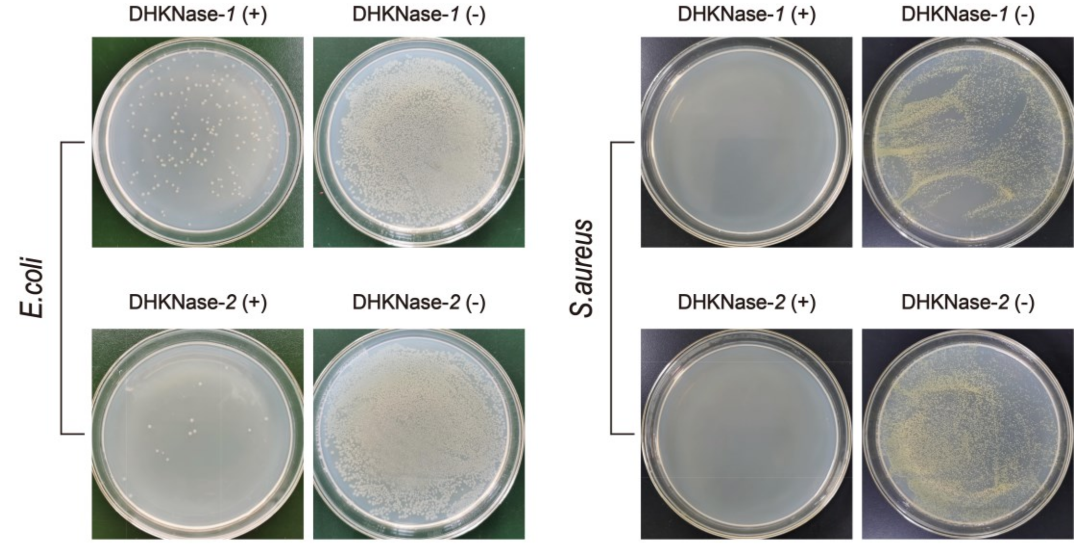

Figure S14. Bactericidal capacity evaluation of the hypericin (DHKNase-*1*) or emodin (DHKNase-*2*). In the experiment, it was identified the excellent killing abilities of the DHKNase-*1*- or DHKNase-*2*-mediated catalytic system against both *E. coli* and *S. aureus* (DHKNase-*1* (+) or DHKNase-*2* (+)), whereas bacteria can maintain a large growth amount with hypericin or emodin alone incubation (DHKNase-*1* (-) or DHKNase-*2* (-)). DHKNase-*1* (-): DHKNase-*1* + buffer; DHKNase-*1* (+): DHKNase-*1* + buffer + H_2O_2; DHKNase-*2* (-): DHKNase-*2* + buffer; DHKNase-*2* (+): DHKNase-*2* + buffer + H_2O_2. DHKNase-*1*/ DHKNase-*2*, 10 μM; H_2O_2, 400 μM; pH 4.0; *S. aureus*, OD_{600} = 1.0; *E. coli*, OD_{600} = 0.1; 35 °C; 0.5 h. The NaAc-HAc buffer for *S. aureus* was 1 mM, and for *E. coli* was 4 mM. The volume ratio of the total reaction solution to the *S. aureus*/*E. coli* suspension was 20:1. Photographs were taken after 18 hours of culture. All experiments were repeated three times.

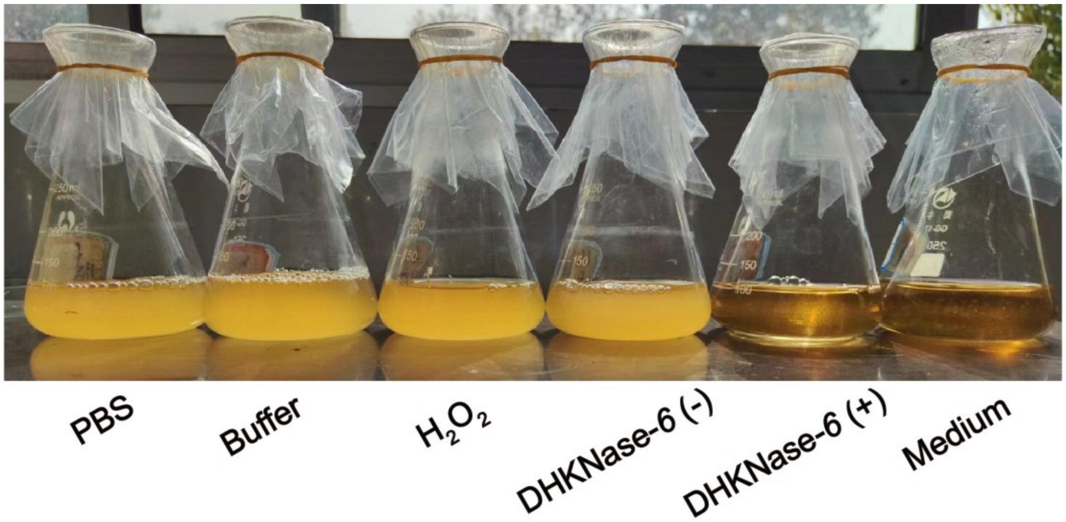

Figure S15. The bacterial suspension culture strategy showed almost no *S. aureus* growth after DHKNase-*6* (+) treatment, indicating efficient bacteria sterilization. DHKNase-*6*, 10 μM; H_2O_2, 400 μM; buffer, pH 4.0 (1 mM); *S. aureus*, OD_{600} = 1.0; 35 °C; 0.5 h. The volume ratio of the total reaction solution to the *S. aureus*/*E. coli* suspension was 20:1. After the incubation bath, the mixture containing the bacteria was inoculated in a sterilized LB liquid medium and cultured. Photographs were taken after 18 hours of culture.

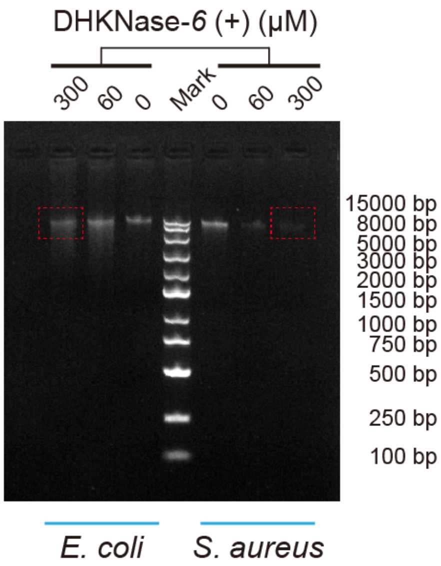

Figure S16. Bacterial DNA degradation after DHKNase-*6* (+) treatment. *E. coli*/*S. aureus*, OD_{600} = 0.4; H_2O_2, 10 mM; NaAc-HAc buffer, pH 4.0 (0.26 M); 35 °C; 5 h. After incubation, the supernatant was removed by centrifugation, the precipitate was washed three times, then DNA was extracted with a standard bacterial DNA extraction kit, and then the extract was directly used for electrophoresis.

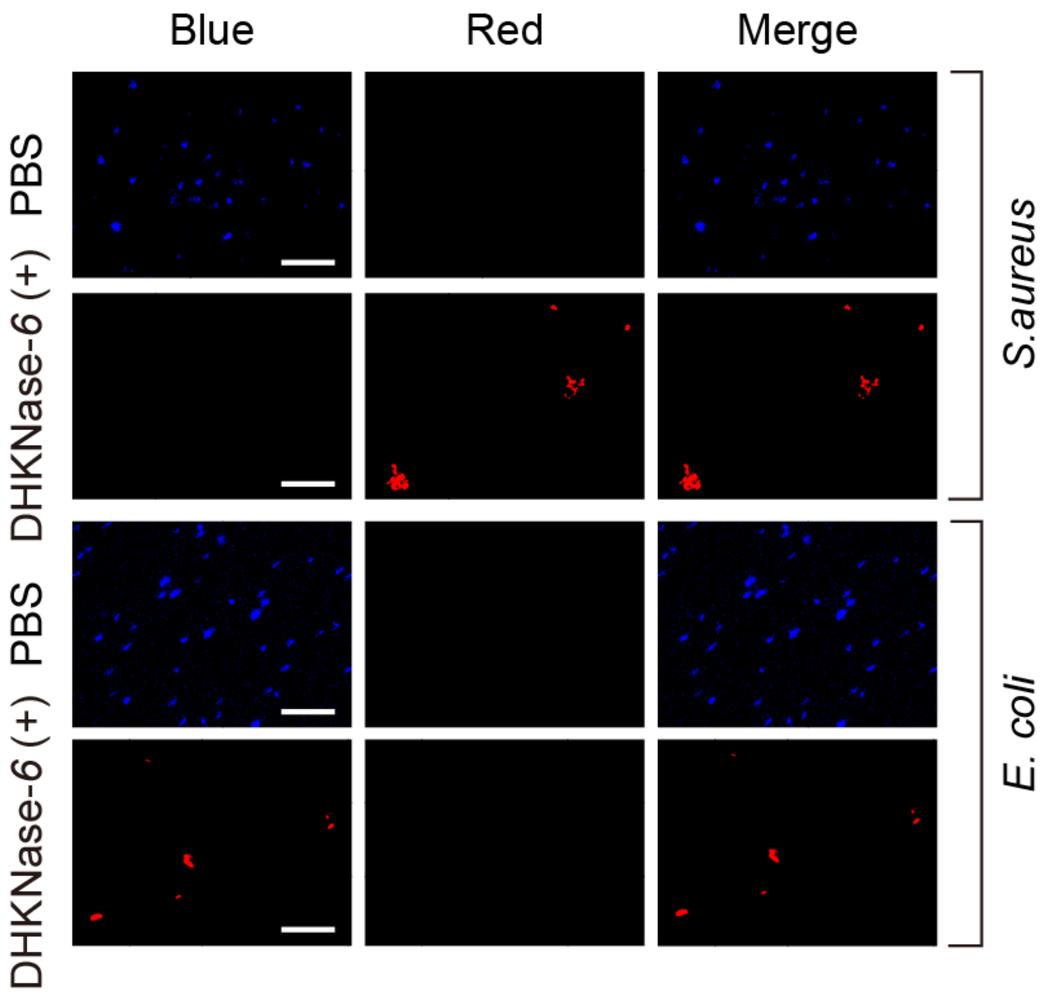

Figure S17. Fluorescence staining images of the *S. aureus* and *E. coli* after the PBS or DHKNase-*6* (+) incubation, in which the PBS group showed more live bacteria (blue) while the group with DHKNase-*6* (+) treatment showed more dead bacteria (red), scale bar: 20 μm. The working concentrations for DHKNase-*6* and H_2O_2 were 10 μM and 400 μM, respectively. After the incubation bath, the mixture was centrifuged to remove the supernatant. Hochest 33342 (200 μg ml^{-1}) and Propidium Iodide (PI) (200 μg ml^{-1}) were used to dark bath the precipitate in the ice water for 0.5 h. Fluorescence images were taken at a microscope with a fluorescence module.

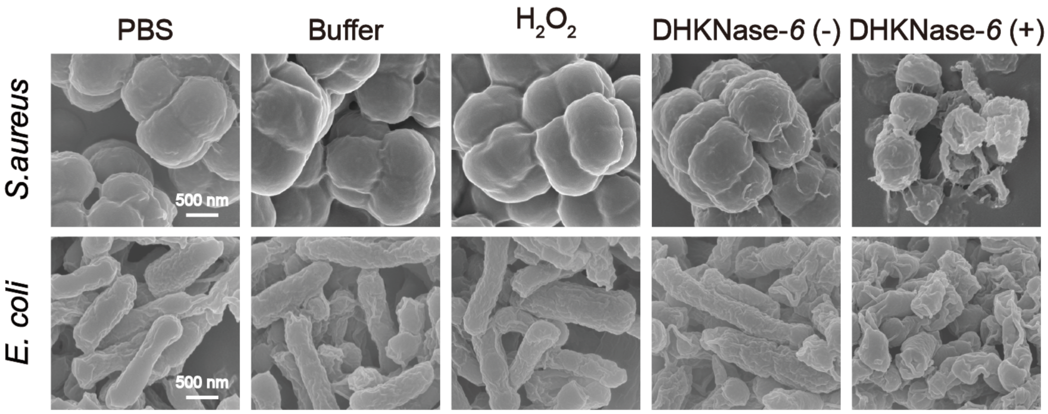

Figure S18. SEM images of *S. aureus* or *E. coli* after PBS, buffer, H_2O_2, DHKNase-*6* (-) or the DHKNase-*6* (+) treatment, in which the bacteria membrane was broken and missing. Scale bar, 500 nm. DHKNase-*6*, 10 μM; H_2O_2, 400 μM; buffer, pH 4.0 (for *S. aureus*: 1 mM; for *E. coli*: 4 mM); 35 °C; 0.5 h. After the reaction, the bacteria were fixed with 4% paraformaldehyde, then dehydrated by a sequential treatment, freeze-dried. The dried bacteria were sputter-coated with gold and then imaged at a field emission scanning electron microscopy (JSM-IT700HR, Jeol) with an energy spectrum instrument (Xplore 30, Oxford).

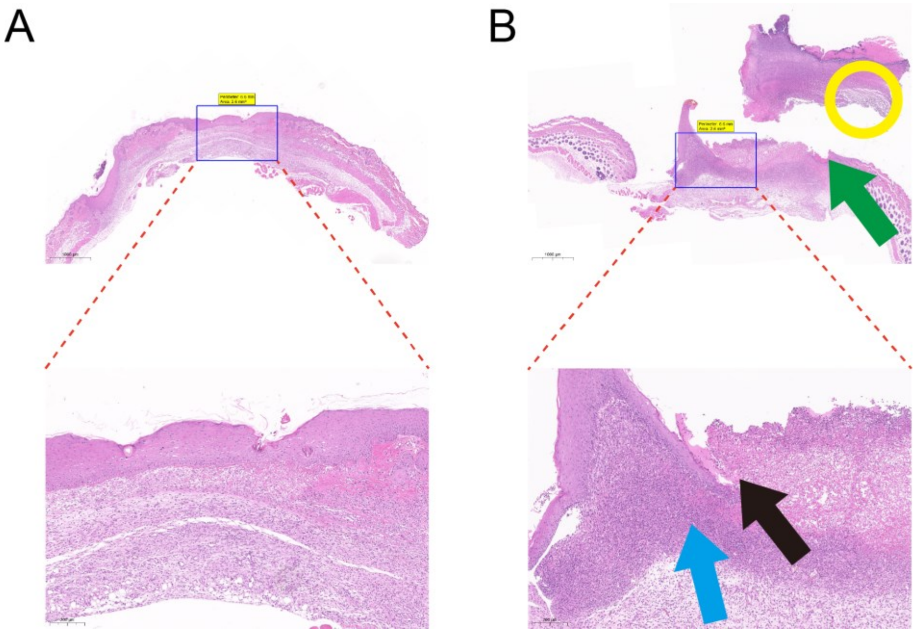

Figure S19. Establishment of infection as well as the inflammation in mouse superficial wound. H&E staining of **A)** *S. aureus*-free wound and **B)** *S. aureus*-infected wound at the fourth day, in which the bacterial infection significantly increased the number of infiltrating inflammatory cells (blue arrow), local edema (black arrow), wound tear (green arrow), and disintegration of skin and collagen (yellow circle) in the wound compared with the wound without infection, demonstrating the successful establishment of wound inflammation.

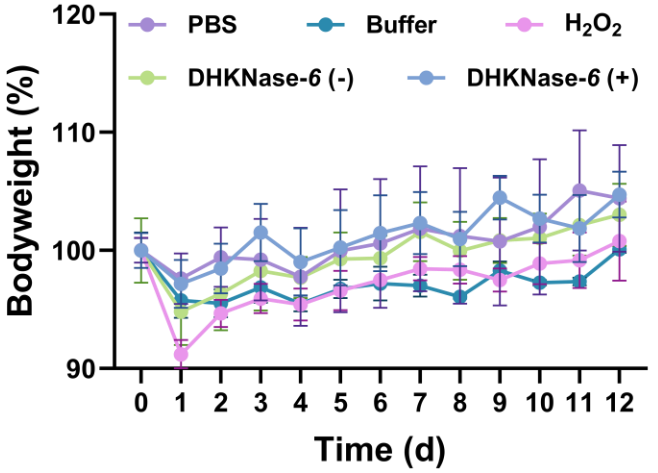

Figure S20. Body weight measurement of mice during the wound healing period. On the first day, mice (n = 3) in all groups experienced a significant weight loss, which was related to the epidermal trauma on the back (n, numbers of mice, mean ± SEM). After the mice adjusted to the wounds on their backs, they slowly regained weight. PBS, 10 mM (pH = 7.4); NaAc-HAc buffer, 1 mM (pH = 4.0); H_2O_2, 200 μM; DHKNase-*6*, 200 μM; *S. aures* suspensions, 1.0×10^7 CFU mL^{-1}. Once the dorsal wound was obtained, *S. aures* suspensions were applied to the wound to establish an infection model. Applied different treatments after one hour of infection.

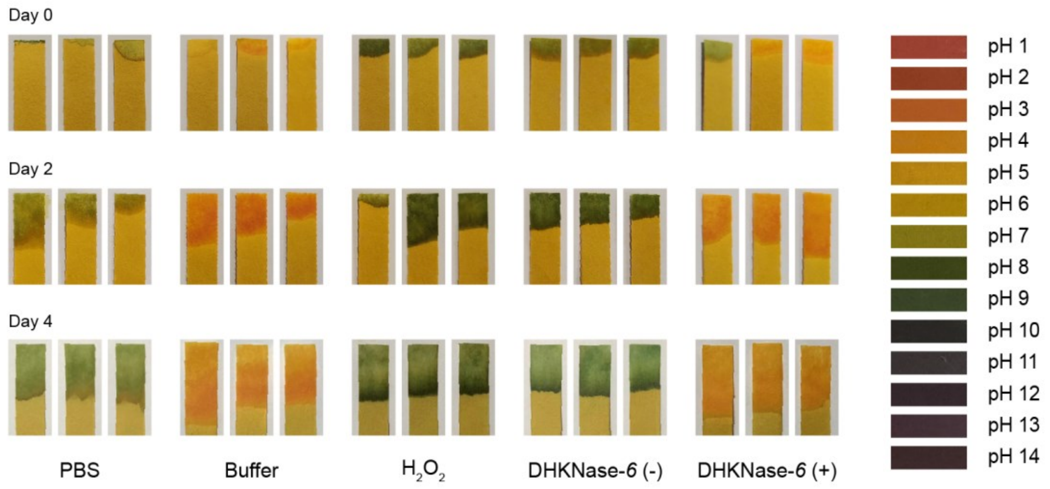

Figure S21. Direct pH measurement of wound exudates using pH dipsticks after the three doses of DHKNase-*6* treatment, showing a guaranteed working environment for DHKNase-*6* to exercise its catalytic function to provide free radicals for bacterial killing.

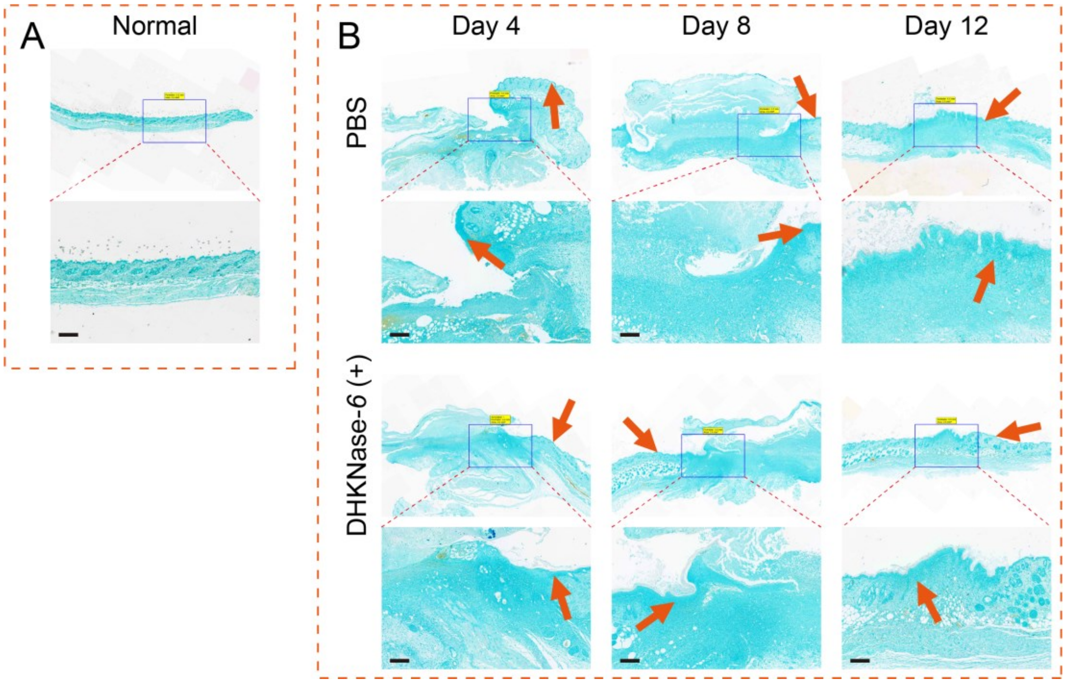

Figure S22. The acidic environment of skin tissue. Alcian staining results showed that **A)** the normal skin tissue and **B)** the stability of acidic wound microenvironment even after 12 days of three doses treatment of DHKNase-*6*. Orange arrows: distinctly acidic tissue. Scar bar: 200 μm. As a cationic dye, alcian blue is the most specific dye for displaying acidic mucoid substances. It can form insoluble complexes with anionic groups (such as carboxyl and sulfate) contained in tissues, thereby showing blue.

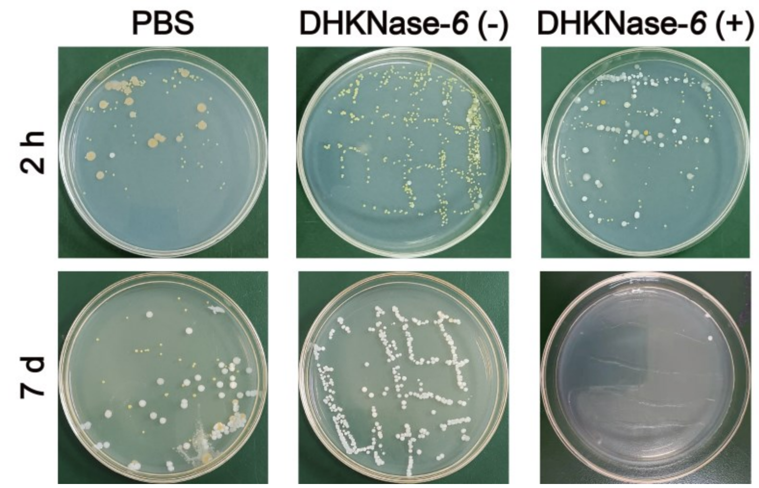

Figure S23. The LB agar medium scribing culture of bacteria on the mice (*n* = 3) wound after treatments as depicted in Figure S17 (*n*, numbers of mice). A significant reduction of bacterial number was observed in the DHKNase-*6* (+) group on day 7, indicating that DHKNase-*6* (+) treatment effectively eliminated the harmful bacteria. Photographs were taken after 36 hours of culture.

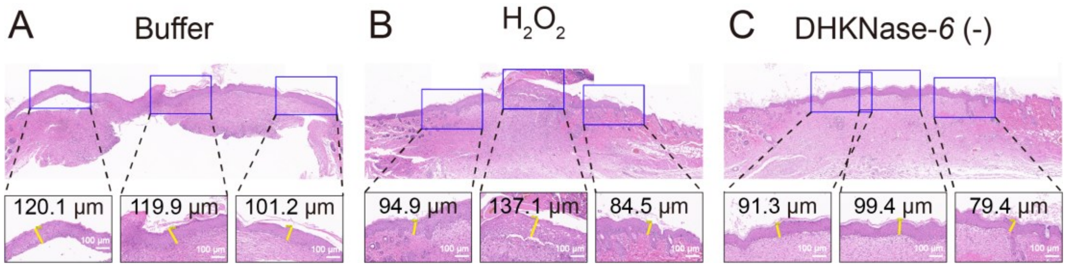

Figure S24. Evaluation of scars' thickness through H&E staining results. (A-C) Buffer, H_2O_2, and DHKNase-*6* (-) treated mice (*n* = 3) wound tissue, showing the slightly reduced scar thickness after DHKNase-*6* (-) treatment (*n*, numbers of biologically independent samples). Scale bar, 100 μm.

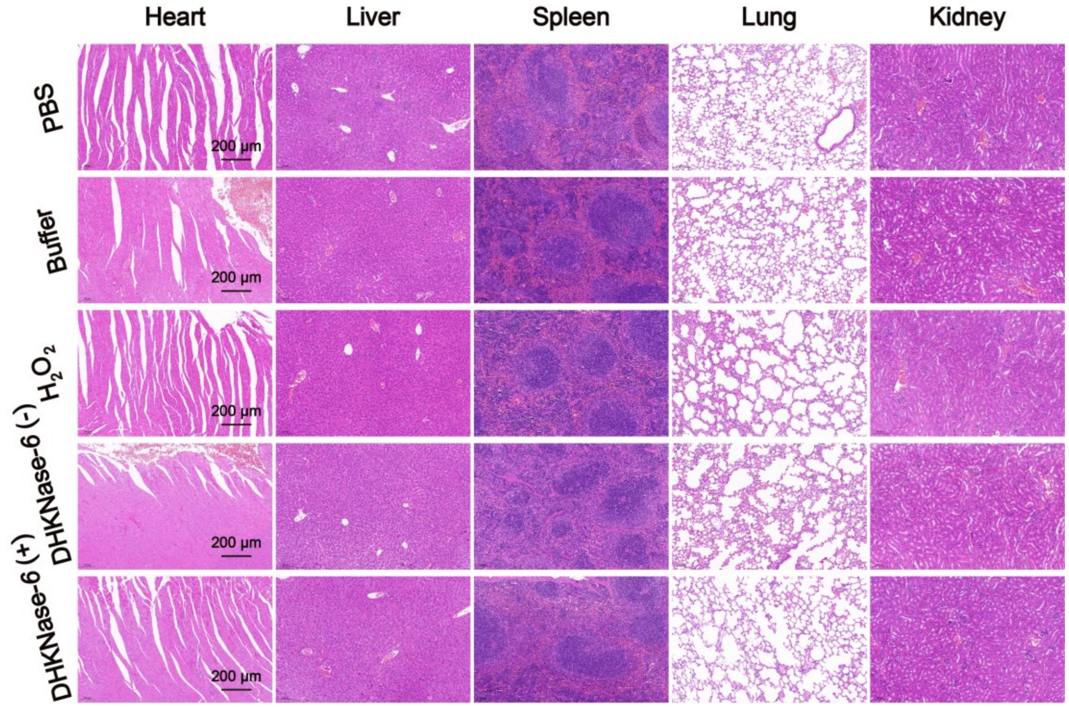

Figure S25. H&E-stained major organ tissue slices after treatments, as depicted in Figure S17, mice were sacrificed, and samples were collected on the 14th day after the initial treatment. Results (n = 3) showed that the instillation of DHKNase-*6* (+) treatment at the infection wound did not pose a health threat to the host mice (n, numbers of mice). Scale bar, 200 μm.

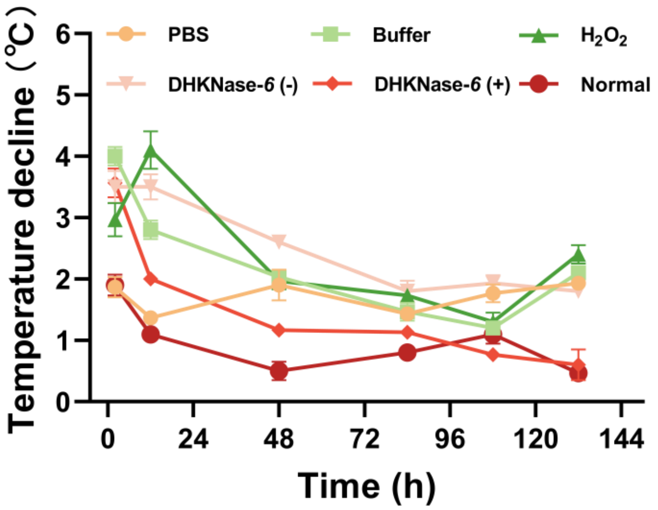

Figure S26. Wound temperature declines in mice of Figure S17. The 'Normal' represented the mice back trauma with no *S. aureus* infection. The temperature variation (n = 3) of the DHKNase-*6* (+) group was similar to that of normal non-infected wounds, implying that after the treatment of DHKNase-*6* (+), the wound gained healthy physiology and obtained a better healing process that was not affected by bacteria infection. Accordingly, the scab in the wound formed faster, guaranteeing a quicker temperature recovery (n, numbers of mice, mean ± SEM).

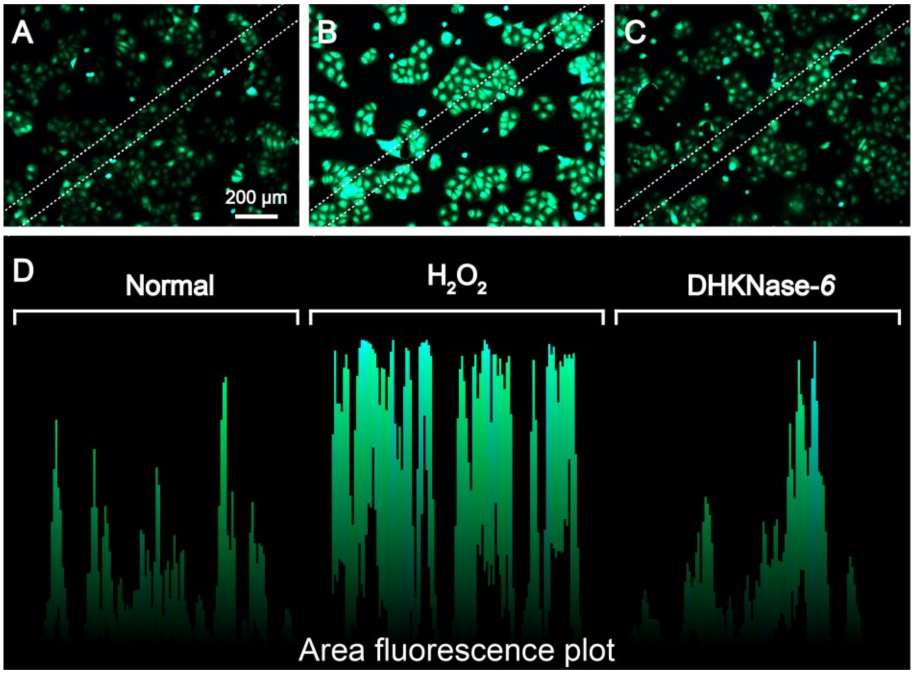

Figure S27. The intracellular ROS-scavenging ability of DHKNase-*6*. *Caco-2* cells (5×10^5 cells/well) were seeded in a 12-well plate and incubated for 12 h. Cells were first treated with DCFH-DA (1 mL, 10 μM) in a serum-free medium and incubated for 30 min. Then, the cells were disposed with MEM-α (10% FBS) solutions containing DHKNase-*6* (0.5 mM) with H_2O_2 (1 mM) for 30 min. **(A-C)** Cells with no treatment were used as negative control (Normal), and cells with only H_2O_2 treatment were used as the positive control (H_2O_2) compared with the experimental assay (DHKNase-*6*). Afterward, the cells were imaged by a microscope camera (MSX2-H) with a fluorescence module (MF-OBGU-LED). The images showed that the ROS level in the cells was significantly reduced after DHKNase-*6* treatment. **(D)** According to the area fluorescence plot map, the DHKNase-*6*-treatment group exhibited a lower mean fluorescence intensity that was near to the normal group, indicating that DHKNase-*6* could effectively alleviate the ROS level in the cells. Image J software was used to draw the area fluorescence plot map.

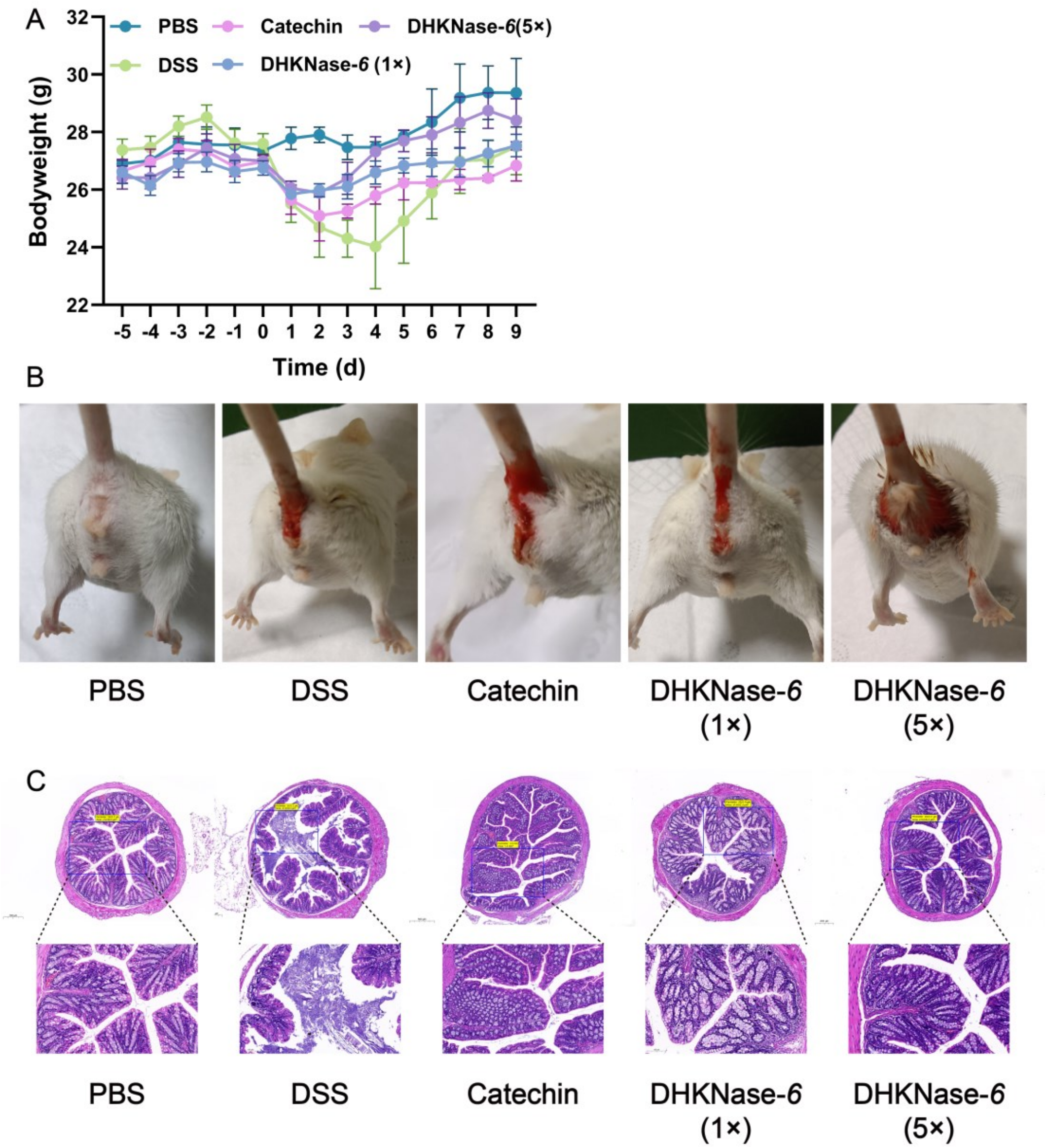

Figure S28. DSS-induced colonic inflammation. A) The body weight of the mice was recorded from days -5 to 9. It was found that the mice fed with 5%DSS in drinking water showed a continuous loss of body weight from the -3 day, while the mice fed with PBS showed a slight increase. **B)** After DSS-induction, hematochezia was observed in the anus of DSS-treated mice at day 0 in the figure of **A)**, but normal in the PBS-treated mice anus. **C)** Morphological staining of colon sections. Among them, even after 9 days of self-healing, the colon of DSS group still showed severe damage.

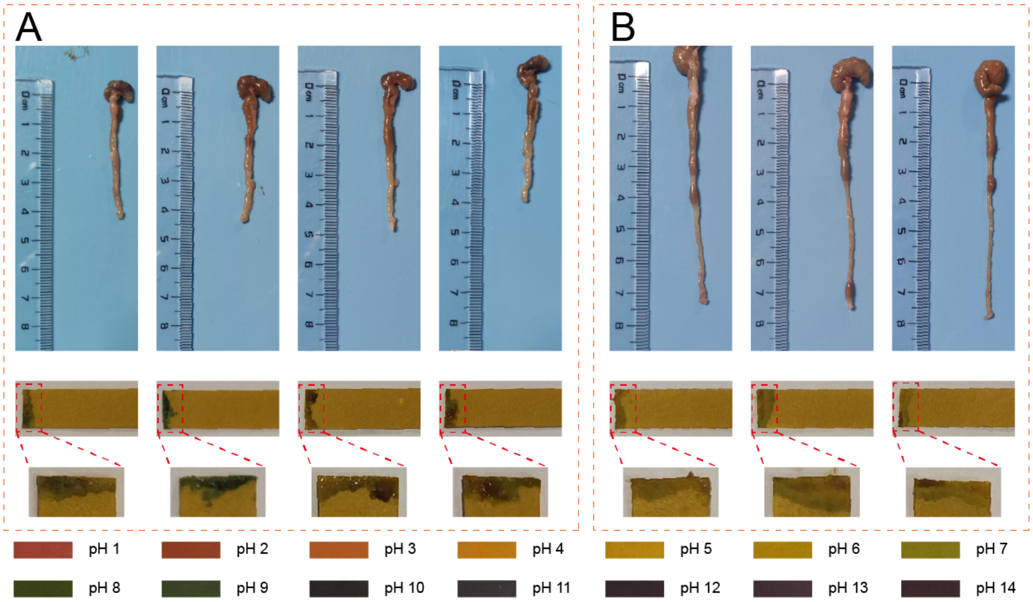

Figure S29. Colon microenvironment pH measurement using pH test tips. A) DSS-induced and **B)** non-induced colon both exhibited pH values greater than 7.

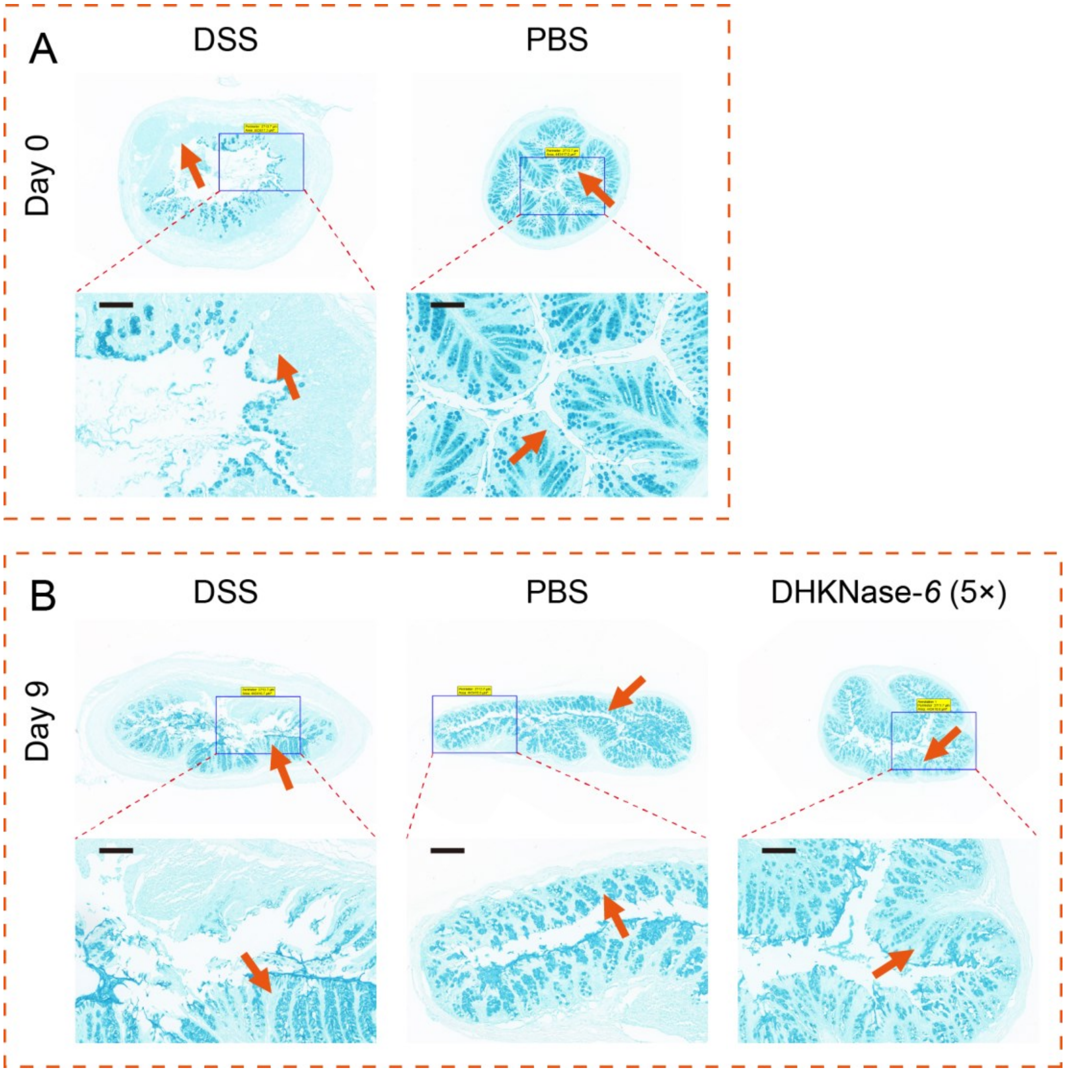

Figure S30. The nonacidic environment in the colonic lumen. A) Even though the colon was induced for 7 days of DSS treatment and showed severe loss of intestinal epithelial layer, there still showed non-acidic intestinal lumen microenvironment. **B)** Alcian staining showed a stabilized neutral intestinal tissue microenvironment and acidic goblet cells with either five doses of the DHKNase-6 treatment or no treatment on day 9. Scar bar: 100 μm.

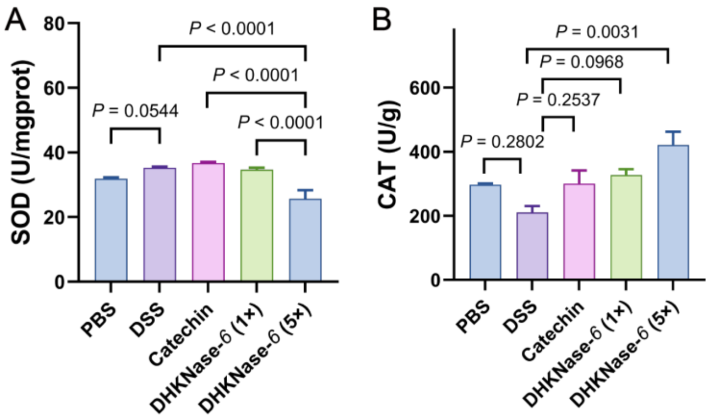

Figure S31. Measurement of SOD and CAT activity in colon tissue of mice in Figure 5d. (A and B) The results indicated that the SOD activity declined while CAT activity increased the in the mice colon (*n* = 3) after DHKNase-*6* treatment, which might thanks to the result of ROS elimination-related the inflammation suppression, indicating the admirable therapeutic effect of DHKNase-*6* on colon inflammation (*n*, numbers of mice, mean ± SEM). After nine days of treatment, mice were sacrificed, and the colon was collected and homogenized, centrifuge to remove sediment, and supernatant for assay kits studies. One-way ANOVA test.

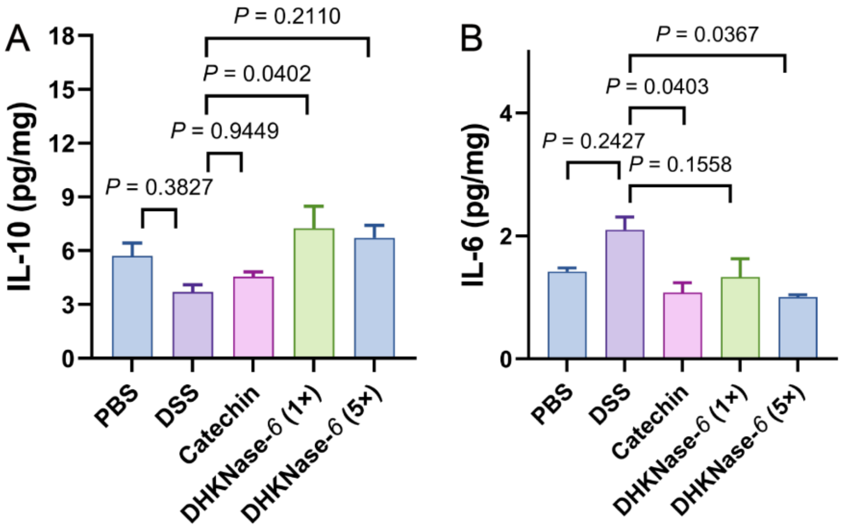

Figure S32. Regulated cytokines in the mice gut after DHKNase-*6* treatment. (A and B) The anti-inflammatory factor IL-10 and pro-inflammatory factor IL-6 (n = 4) were upregulated and downregulated in the DHKNase-*6* (5×) mice colon (n, numbers of mice, mean ± SEM), respectively. One-way ANOVA test.

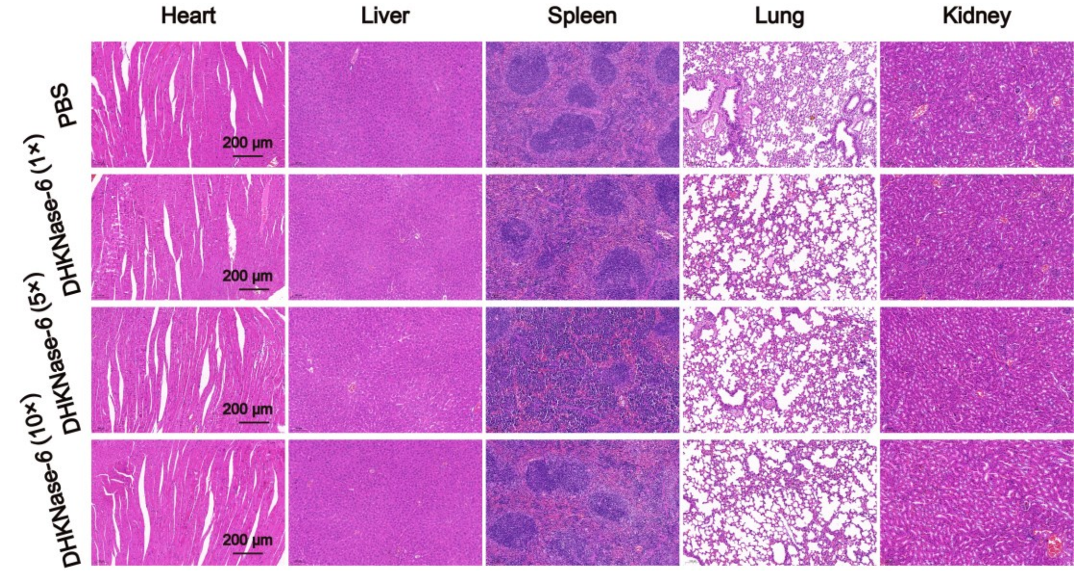

Figure S33. H&E-stained major organ tissue slices from different tail vein injection groups. Healthy male BALB/c mice were acclimated for a week, followed by tail intravenous injections of 100 microliters of PBS, or DHKNase-*6* (DHKNase-*6* (1×), 30 mg kg^{-1}; DHKNase-*6* (5×), 150 mg kg^{-1}; DHKNase-*6* (10×), 300 mg kg^{-1}). Mice (n = 3) were dedicated on the fourteenth day, and samples were then collected and soaked in tissue fixation solution for the next staining experiment (n, numbers of mice). Results demonstrated that even after 10× (300 mg kg^{-1}) DHKNase-*6* treatment, the mouse physiology was indistinguishable from healthy mice, indicating the excellent biocompatibility of DHKNase-*6,* posing no harm to the health of the host mice. Scale bar, 200 μm.

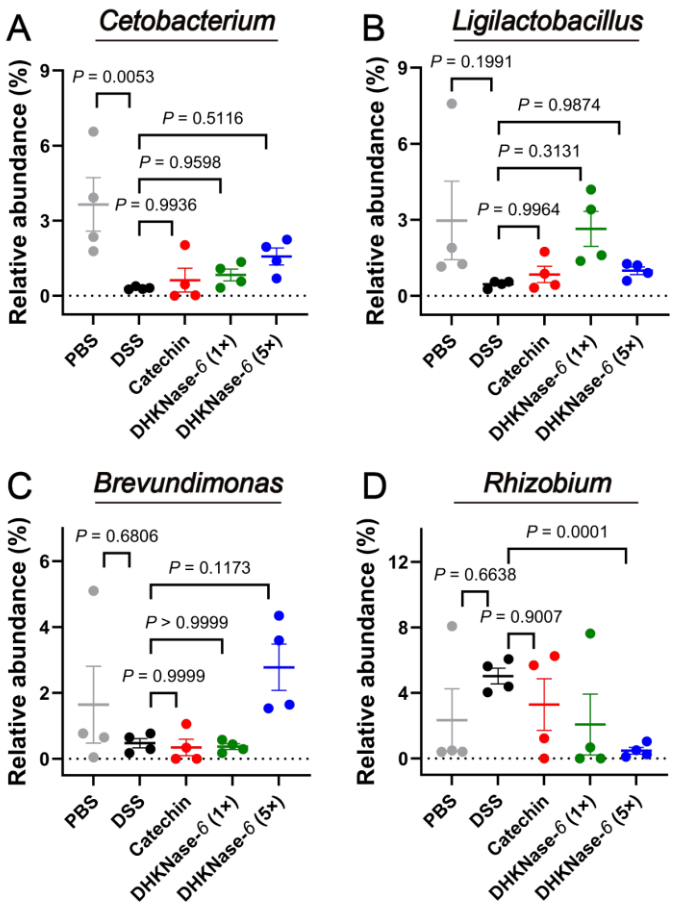

Figure S34. DHKNase-*6* regulated the host intestinal flora. (A-D) Relative abundance of taxa *Cetobacterium* (known to produce acetate and promote glucose homeostasis in the intestine), *Ligilactobacillus* (known to produce lactate and promote intestinal homeostasis), *Brevundimonas* (known to be almost absent in the enteritis model), and *Rhizobium* (known as IBD characteristic flora) (n = 4) in different treatment groups (n, numbers of mice, mean ± SEM). The results showed that the proportion of beneficial bacteria (*Cetobacterium*, *Ligilactobacillus*) increased while the proportion of harmful bacteria (*Rhizobium*) decreased after DHKNase-*6* treatment. On the ninth day after the first treatment, mice were sacrificed, feces were collected and -80 degrees saved, dry ice transported to the testing company for RNA extraction and analysis. Each dot represented a treatment mouse. One-way ANOVA test.

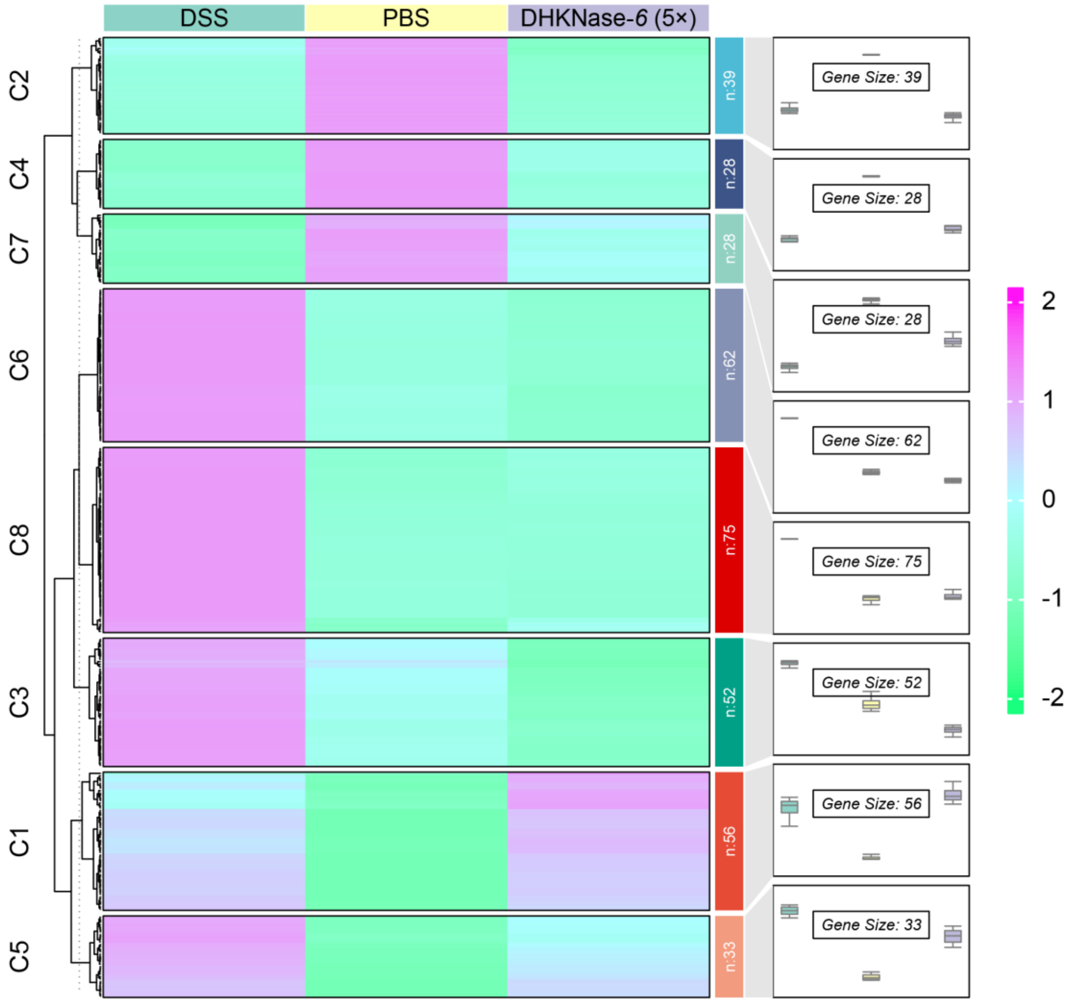

Figure S35. Weighted gene co-expression network analysis map of the PBS, DSS, and DHKNase-*6* (5×) group mice. It can be seen that after DHKNase-*6* (5×) treatment, the gene expression in the gut (n = 3) was significantly reversed to be more similar to the expression of the PBS group, based on 189 genes in the eight modules (right) in the cluster analysis (left) (n, numbers of mice, mean). Each node was defined as a gene, and the genes with shared expression in different samples were in the same gene network, and a hierarchical clustering tree was constructed accordingly. The co-expression degree of genes in the same module was high, while the co-expression degree of genes belonging to different modules was low. Mice colon tissues were collected on the ninth day, rinsed off the contents, -80 degrees preserved, dry ice transported to the testing company for extraction and analysis.

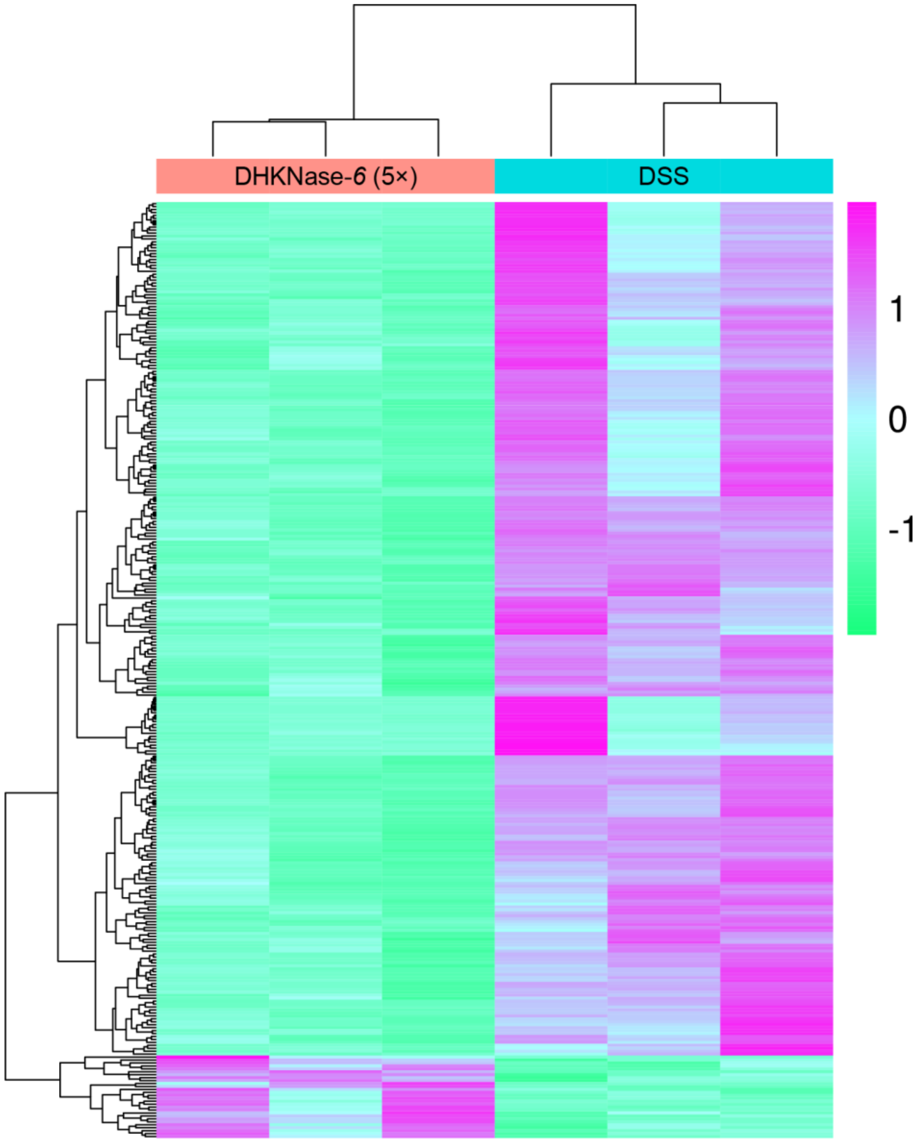

Figure S36. Analysis of gene co-expression in the colon of DSS-induction and DHKNase-*6*-treated mice colon (*n* = 3) based on the 350 characterized genes, differentiated gene expression in the gut was observed, demonstrating that DHKNase-*6* treatment regulated the host gut gene expression (*n*, numbers of mice). Each column represented a treatment mouse.

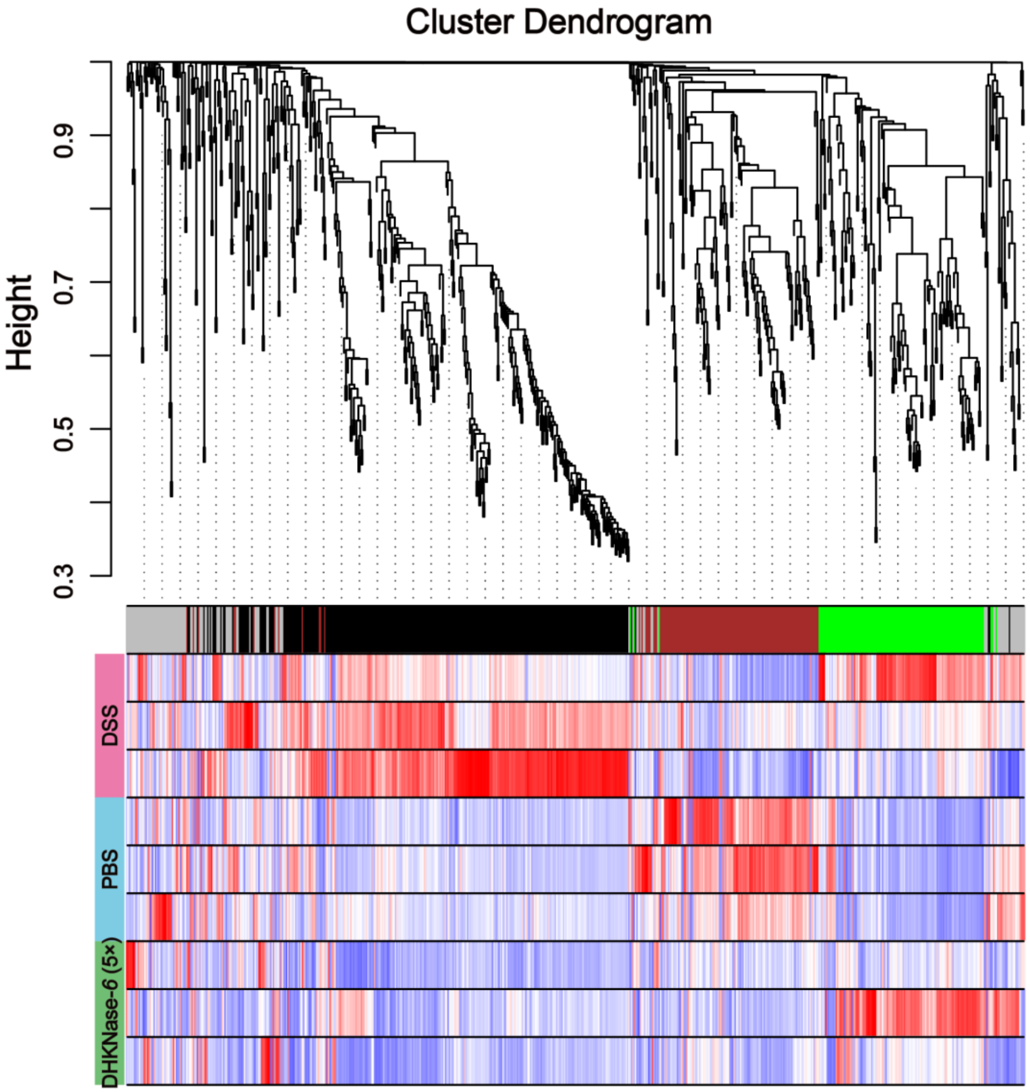

Figure S37. Gene co-expression map and cluster tree of DSS, PBS, and DHKNase-*6* (5×) group mice (*n* = 3) colon based on the 424 characterized genes (*n*, numbers of mice). The analysis was used to identify the common expression patterns of differentially expressed genes, analyze the different change patterns of mRNA expression abundance among samples, divide the mRNA with the same expression trend into a dataset, and make the expression pattern map of the dataset. Euclidean distance, hierarchical clustering. Taking the leaf node as the starting point, the tree height ratio was calculated, the clustering tree was cut according to the specified percentage, and the subtree corresponding to the cutting position was taken as the final clustering class. Each row represented a treatment mouse.

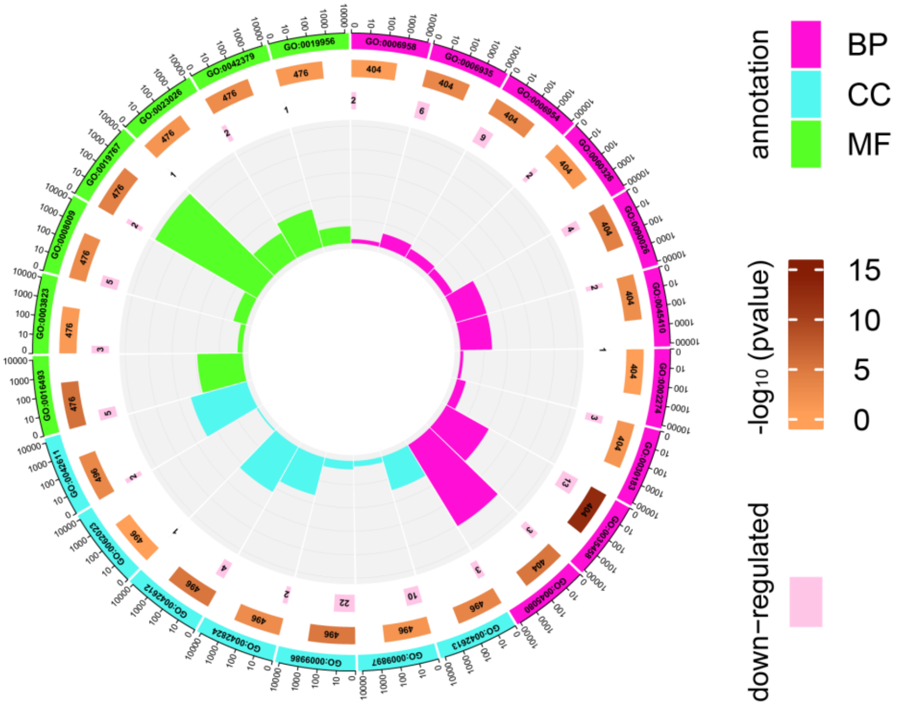

Figure S38. GO enrichment analysis between DSS and PBS group mice (n = 3) based on the biological process (BP), cellular component (CC), and molecular function (MF) annotation (n, numbers of mice; mean), in which the cellular pathway expression of monocyte chemotaxis (GO:0090026), interleukin-6 biosynthetic process (GO:0045410), cellular response to interferon-beta (GO:0035458), chemokine biosynthetic process (GO:0045080), MHC class II protein complex (GO:0042613), MHC class I peptide loading complex (GO:0042824), MHC class I protein complex (GO:0042612), MHC protein complex (GO:0042611), C-C chemokine receptor activity (GO:0016493), IgE receptor activity (GO:0019767), and chemokine receptor binding (GO:0042379) were significantly upregulated in DSS group mice colon. From the outside to the inside, it is the following: classification (the same color is the same classification), the total number of genes in term (the number of genes contained in the term, the color indicates the -$\log_{10}$ p value of the term), the number of genes upregulated, and rich factor.

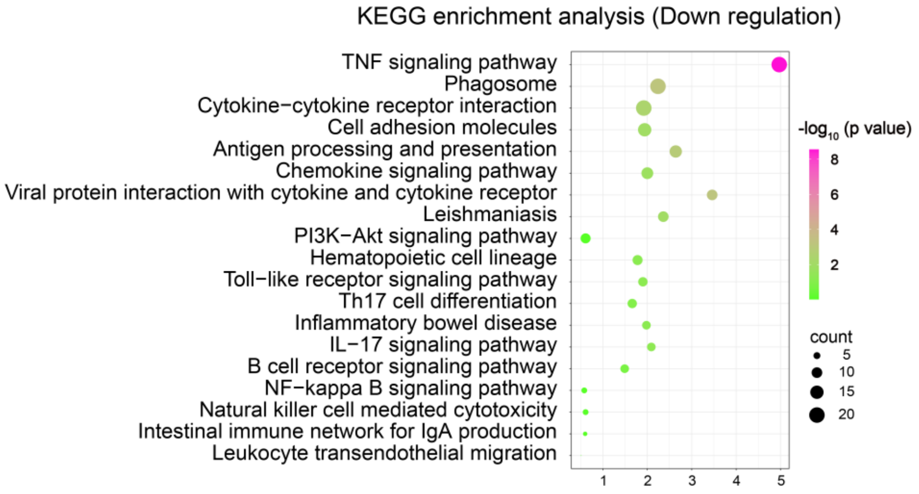

Figure S39. KEGG enrichment analysis (n = 3) of the down-expressed cell pathway in PBS mice compared to the DSS-induced mice (n, numbers of mice; mean), in which the TNF signaling pathway, Th17 cell differentiation, NF-kappa B signaling pathway, and IL-17 signaling pathway were significantly upregulated after DSS induction. FDR=0.01.

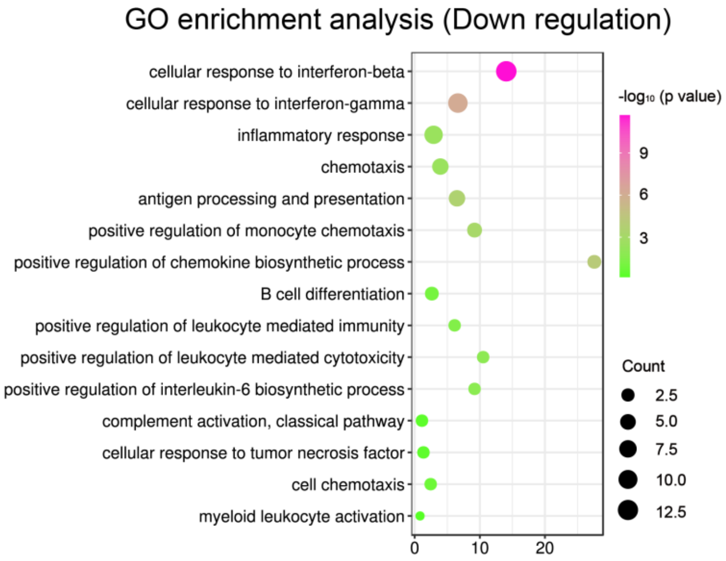

Figure S40. GO enrichment analysis (n = 3) of the down-expressed cell pathway in PBS mice compared to the DSS-induced mice (n, numbers of mice; mean). The typical inflammatory response, chemotaxis, and myeloid leukocyte activation signaling pathways related to cellular inflammation were significantly upregulated after DSS treatment. Results were from three independent mice, FDR=0.01, mean.

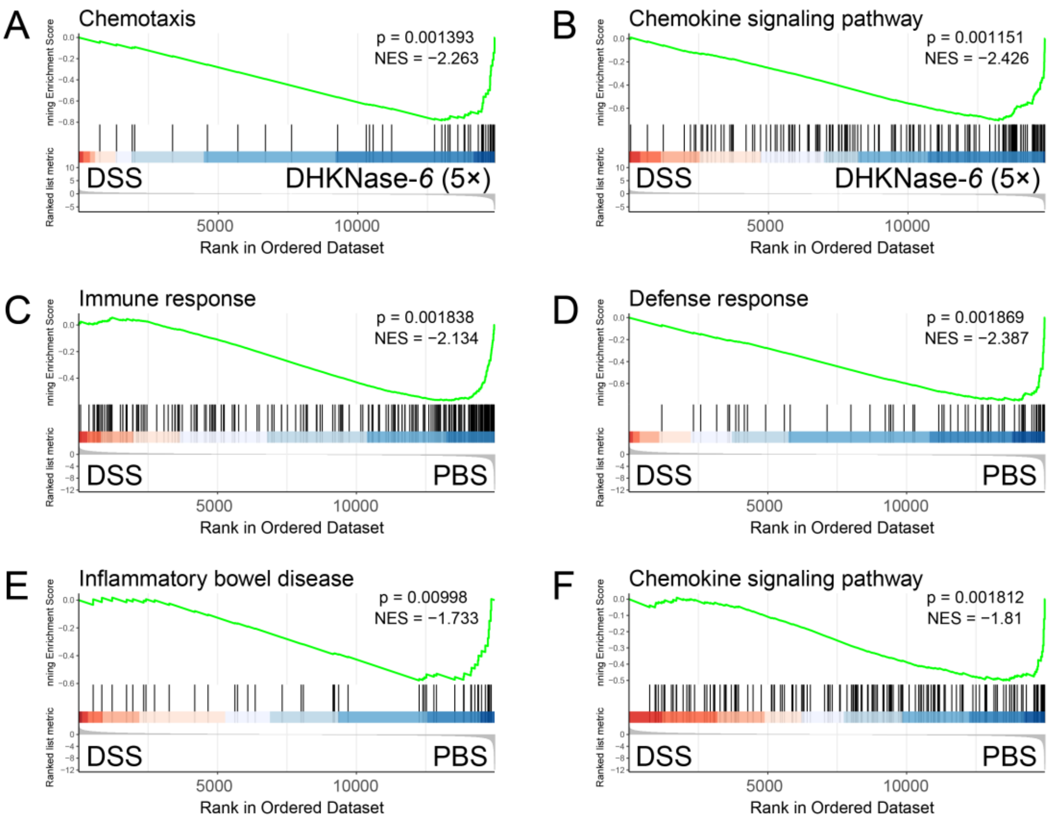

Figure S41. GSEA analysis in the mice colon. (A and B) The downregulated chemotaxis and chemokine signaling pathway in DHKNase-*6*-treated mice (n = 3) colon (n, numbers of mice). **(C-F)** The lower-expressed immune response, defense response, inflammatory bowel disease, and chemokine signaling pathway (n = 3) in PBS mice colon (n, numbers of mice). For each map, on the top was the ES value of the gene set. A peak in the front of the sequence gene set (ES value greater than 0) indicates up-regulation of the pathway, and a peak in the back (ES value less than 0) indicates down-regulation of the pathway. In the middle, if vertical lines were concentrated in the front or back of the gene sequencing list, it indicates that the gene set pathway was upregulated or down-regulated. The gene corresponding to the red part was highly expressed in the DSS group, and the gene corresponding to the blue part was highly expressed in the DHKNase-*6* (5×)/PBS group. The Signal noise ratio (log2FC) corresponding to each gene was shown in the gray area plot (bottom). Results were from three independent mice, mean.

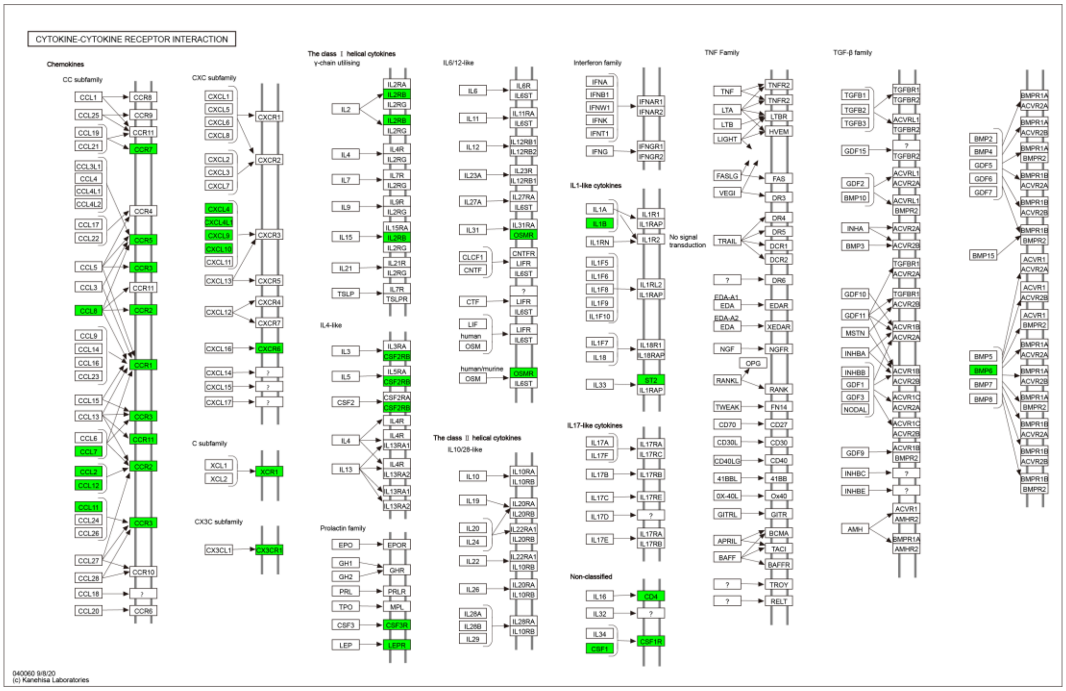

Figure S42. KEGG annotation revealed the significantly downregulated genes (green background) in CYTOKINE-CYTOKINE RECEPTOR INTERACTION of the DHKNase-*6* (5×) treated mice (n = 3) colon (n, numbers of mice).

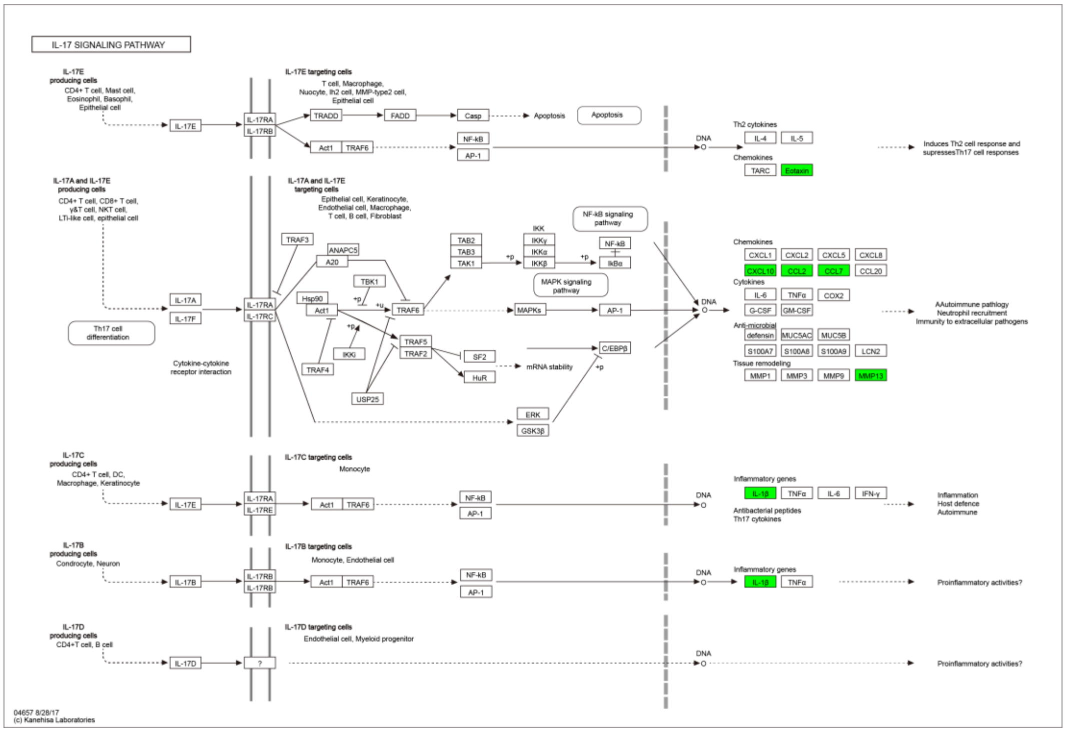

Figure S43. In the DHKNase-*6* (5×) treated mice (*n* = 3) colon (*n*, numbers of mice), KEGG annotation revealed significant downregulated genes (green background) in IL-17 SIGNALING PATHWAY.

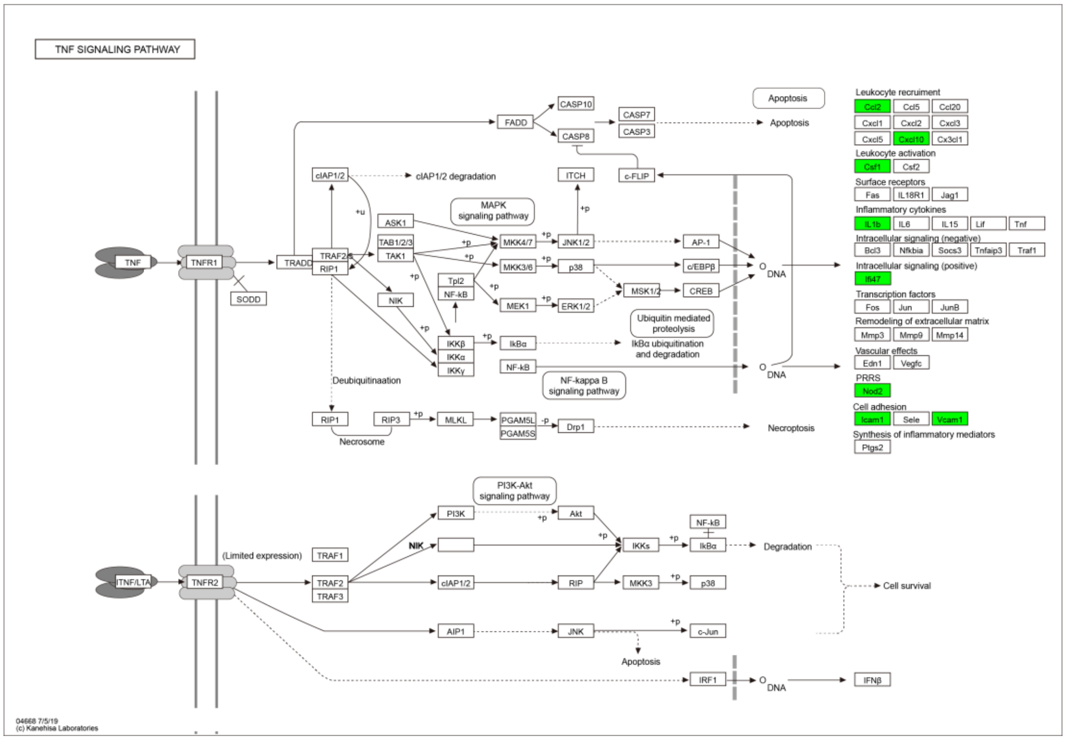

Figure S44. KEGG annotation revealed the significantly downregulated genes (green background) in TNF SIGNALING PATHWAY in the mice (n = 3) colon (n, numbers of mice) with DHKNase-*6* (5×) treatment.

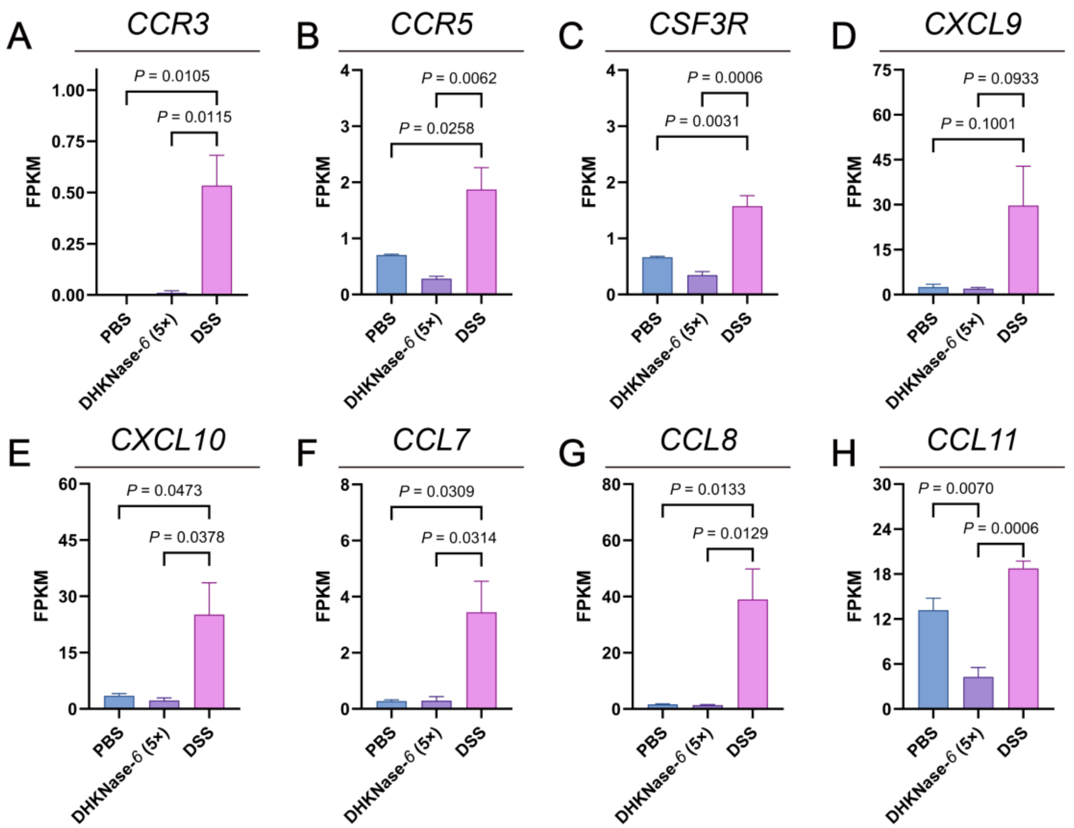

Figure S45. The downregulated cytokines, pro-inflammatory, and IL-17 signaling genes in the DHKNase-*6* (5×) group mice. **(A-H)** The decreased expression of *CCR3*, *CCR5*, *CSF3R*, *CXCL9*, *CXCL10*, *CCL7*, *CCL8*, and *CCL11* genes in mice (n = 3) colon (n, numbers of mice, mean ± SEM) after DHKNase-*6* (5×) treatment. One-way ANOVA test.

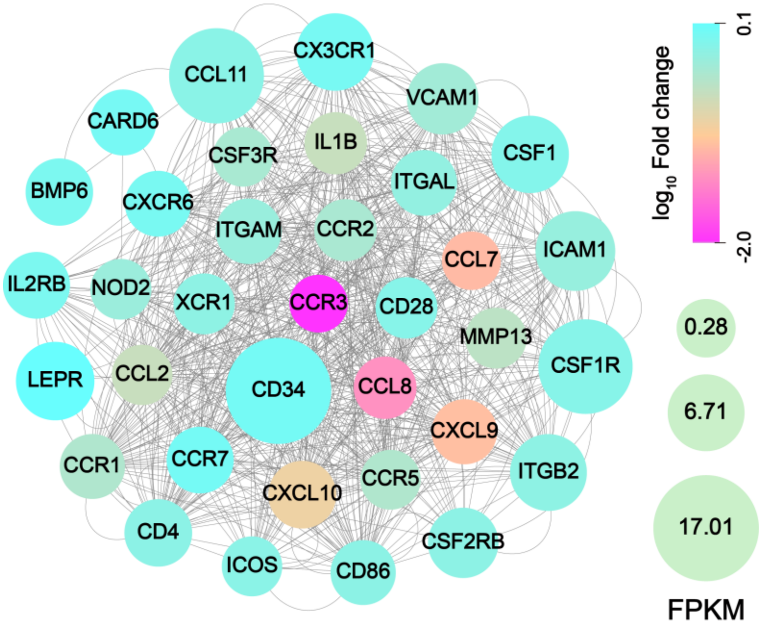

Figure S46. Protein-protein interaction network of DEGs between the DSS-induced and PBS-feeding mice (*n* = 3) revealed that the association of high *CCR3* gene expression with DSS-induced development of colon enteritis (*n*, numbers of mice). Cystoscape software (3.9.1) was adopted to draw the protein-protein interaction network.

Table S1. Elementary composition of the DHKNase-*6* standard measured by ICP-MS

Content	w/w (%)	Content	w/w (%)	Content	w/w (%)	Content	w/w (%)
Li	<0.0001	Ga	<0.0001	Cs	<0.0001	Hf	<0.0001
Na	0.0050	Ge	<0.0001	Ba	<0.0001	Ta	<0.0001
Mg	<0.0001	Rb	<0.0001	La	<0.0001	W	<0.0001
Al	<0.0001	Sr	<0.0001	Ce	<0.0001	Re	<0.0001
K	0.0005	Y	<0.0001	Pr	<0.0001	Os	<0.0001
Ca	0.0017	Zr	<0.0001	Nd	<0.0001	Ir	<0.0001
Sc	<0.0001	Nb	<0.0001	Sm	<0.0001	Pt	<0.0001
Ti	<0.0001	Mo	<0.0001	Eu	<0.0001	Au	<0.0001
V	<0.0001	Ru	<0.0001	Gd	<0.0001	Hg	<0.0001
Cr	<0.0001	Rh	<0.0001	Tb	<0.0001	Tl	<0.0001
Mn	<0.0001	Pd	<0.0001	Dy	<0.0001	Pb	<0.0001
Fe	<0.0001	Ag	<0.0001	Ho	<0.0001	Bi	<0.0001
Co	<0.0001	Cd	<0.0001	Er	<0.0001	Th	<0.0001
Ni	<0.0001	In	<0.0001	Tm	<0.0001	U	<0.0001
Cu	<0.0001	Sn	<0.0001	Yb	<0.0001		
Zn	<0.0001	Sb	<0.0001	Lu	<0.0001		

Table S1. The ICP-MS content test proved the shallow impurity content for purchased DHKNase-*6* (diosmetin) standards. For this, an equal concentration of K^+, Mg^{2+}, Ca^{2+}, Cu^{2+}, and Mn^{2+} ions were added to the reaction solution to exclude the effects of metal ions-mediated decomposition of H_2O_2 and oxidation of TMB.

Table S2. The Michaelis-Menten constant (K_m), the maximum reaction rate (V_{max}), and the catalytic constant (K_{cat}) of DHKNase-*6*.

Catalyst	Substance	K_m [mM]	V_{max} [10^{-9} M s^{-1}]	K_{cat} [s^{-1}]
DHKNase-*6*	H_2O_2	1.79 ± 0.545	11.72 ± 2.125	1.953×10^{-4}
	TMB	0.40 ± 0.050	1.84 ± 0.054	3.06×10^{-5}

Table S2. The calculated Michaelis-Menten equation constants for DHKNase-*6* according to Figure S8.

Reference

1. Wang T, Bai Q, Zhu Z, Xiao H, Jiang F, Du F, Yu WW, Liu M, Sui N. Graphdiyne-supported palladium-iron nanosheets: A dual-functional peroxidase mimetic nanozyme for glutathione detection and antibacterial application. *Chem. Eng. J.* 2021;413:127537.
